# Barriers to access and adherence to tuberculosis services, as perceived by patients: A qualitative study in Mozambique

**DOI:** 10.1371/journal.pone.0219470

**Published:** 2019-07-10

**Authors:** Caroline De Schacht, Cláudia Mutaquiha, Felicidade Faria, Georgina Castro, Nélia Manaca, Ivan Manhiça, James Cowan

**Affiliations:** 1 Health Alliance International, Maputo, Mozambique; 2 The Mozambican National Tuberculosis Program, Maputo, Mozambique; 3 The University of Washington Department of Global Health, Seattle, Washington, United States of America; University Lyon 1 Faculty of Dental Medicine, FRANCE

## Abstract

**Introduction:**

Tuberculosis (TB) continues to be a leading cause of death in Sub-Saharan Africa, including Mozambique. While diagnostic methods and total notifications are improving, significant gaps remain between total numbers of TB cases annually, and the number that are notified. The purpose of this study was to elicit Mozambican patients with drug sensitive TB (DS-TB), TB/HIV and Multi drug resistant tuberculosis (MDR-TB) understanding and assessment of the quality of care for DS-TB, HIV/TB and MDR-TB services in Mozambique, along with challenges to effectively preventing, diagnosing and treating TB.

**Materials and methods:**

Qualitative data was collected via separate focus group discussions consisting of patients with DS-TB, TB/HIV and MDR-TB at four health centers in Sofala and Manica Province, Mozambique, to describe knowledge on TB, HIV and MDR-TB, and identify barriers to access and adherence to services and their recommendations for improvement. A total of 51 patients participated in 11 discussions. Content analysis was done and main themes were identified.

**Results:**

Focus groups shared a number of prominent themes. Respondents identified numerous challenges including delays in diagnosis, stigma related with diagnosis and treatment, long waits at health facilities, the absence of nutritional support for patients with TB, the absence of a comprehensive psychosocial support program, and the lack of overall knowledge about TB or multi drug resistant TB in the community.

**Discussion:**

TB patients in central Mozambique identified many challenges to effectively preventing, diagnosing and treating tuberculosis. Awareness strengthening in the community, continuous quality monitoring and in-service training is needed to increase screening, diagnosis and treatment for TB, HIV/TB and MDR-TB.

## Introduction

Tuberculosis continues to be an epidemic in Sub-Saharan Africa. Mozambique is one of the 22 high burden tuberculosis (TB) countries. The World Health Organization (WHO) estimates a TB incidence rate in Mozambique of 551/100,000 in 2017 resulting in 159,000 new cases each year[1]. This has been largely fueled by the HIV epidemic with a national prevalence of 13.2%, but compounded by deep poverty with an estimated average gross national income per capita of only $480 USD [2, 3]. Of the estimated incident TB cases, 86,515 were put on treatment in 2017, resulting in case detection rate (CDR) of only 54% for all forms of TB, and 943 of 8,800 (11%) of the estimated total multi-drug resistant or rifampin resistant TB (MDR/RR-TB) were notified [1, 4]. TB continues to be a leading cause of death in Mozambique, and the primary cause of death and disability in HIV patients [1, 4].

The morbidity and mortality burden of TB and especially drug-resistant tuberculosis are unacceptably high in many countries including Mozambique[5]. With current trends in tuberculosis incidence, reaching the Sustainable Development Goal target to end the tuberculosis epidemic by 2030 will be a major challenge[5].

Globally there is a growing interest not only in the total number of patients diagnosed and treated for TB, but the quality of TB related healthcare service delivery. Recently, The Lancet Global Health published a seminal report entitled “High-quality health systems in the Sustainable Development Goals era: time for a revolution” [6]. The authors “assert that providing health services without guaranteeing a minimum level of quality is ineffective, wasteful, and unethical”. What is needed, the Commission argues, is political commitment to create high-quality health systems that optimize health care in each given context by consistently delivering care that improves or maintains health, by being valued and trusted by all people, and by responding to changing population needs. Throughout the report TB is used as an example to show that we need to move beyond just evaluating countries by total notifications, and also focusing on evaluating the quality of care.

Analyzing data from 137 countries, the authors estimate that 50% of the 900,000 people who do die from TB each die because of poor quality of care, and the other 50% due to non-utilization of healthcare resources[7]. Limited information about the quality of TB care service delivery is available in Mozambique, particularly incorporating the perspective of individual patients.

In Mozambique, TB screening and diagnosis is primarily carried out among patients at health centers complaining of TB-related symptoms using smear microscopy (SS) on sputum samples, or increasingly using GeneXpert MTB/RIF (Xpert) testing as recommended by the WHO [8]. In Mozambique 96% of new TB patients are tested for HIV and 40% of TB patients in Mozambique are co-infected with HIV–of these 95% started antiretroviral therapy in 2017 [1, 4]. The treatment success rate was 90% for new drug-sensitive TB patients that were notified in 2016 [1, 4].

Given the global burden of TB and MDR/RR-TB, improved diagnostics, access to treatment, and high-quality care are priorities for the Mozambican National Tuberculosis Program (NTP) and the global health community. As a result, Health Alliance International (HAI), a global health NGO with over 25 years of experience working in Mozambique, in partnership with the NTP and provincial health departments supported early pilot studies of LED SS microscopy, Xpert, and an innovative mHealth platform called GxAlert at several sites in Manica and Sofala Province, along with quantitative and qualitative assessments of the TB care cascade [9, 10].

Prior to this study there was no data from Mozambique and in these specific provinces about patient’s knowledge and perceptions regarding TB care. In partnership with the provincial health authorities, this qualitative study was conducted to explore TB, TB/HIV and MDR-TB patient’s knowledge about their disease and to elicit their views of the quality of current TB services, and prevention strategies. In particular this study sought to understand barriers as perceived by patients to access care TB services, especially at facilities with a GeneXpert machine.

## Materials and methods

Investigators selected a convenience sample of four urban, semi-urban and rural government health facilities across Manica (Eduardo Mondlane and Gondola health facilities) and Sofala (Nhamatanda, and Ponta Gea health facilities) Province where HAI had ongoing TB related activities. Purposeful sampling was used. Patients attending the TB services were informed by the TB nurse about the objectives of the study. Interested people were referred to the research team for more information and consenting. Eligible subjects were restricted to non-infectious adults with DS-TB, TB/HIV or MDR/RR-TB. Written informed consent was obtained from all subjects prior to data collection.

From February to March 2016, three data collectors (2 female, 1 male), experienced in qualitative research methodology, visited each of the four study sites. Before data collection, they were trained on the protocol by the investigators. Qualitative data were collected via focus group discussions (FGD) with patients. Investigators developed a discussion guide that focused on knowledge of TB, MDR-TB and HIV, barriers for access and adherence to care, and suggestions for improvement. The guide was composed of the following sections: 1) knowledge regarding TB, HIV, MDR-TB; 2) perceived barriers to access TB-care; 3) perceived barriers to adherence; 4) suggestions from participants for improvement. Themes/Sub-themes were based on results of a similar study [11] enriched with program-driven hypothesized sub-themes.

Participants were grouped by diagnosis: DS-TB, MDR/RR-TB, TB/HIV co-infection in order to minimize the potential biasing effects that stigma associated with HIV and MDR-TB might have on a frank discussion and to allow directed discussion of pertinent areas of inquiry. The discussions were done in Portuguese and local language was used when participants showed difficulties in understanding (Siteu, Barué, Sena, Ndau). There was no recording of the discussions per original study design. Notes were taken during the discussions which were then transcribed to MS Word into Portuguese and de-identified.

Data were analyzed using an approach informed by content analysis[12], assisted by the software MAXqda Version 11 (verbi GmbH, Berlin, Germany). Investigators CM, CDS and FF independently read transcripts to identify units of meaning regarding subjects’ beliefs about barriers to TB care. A list of codes was created based on this initial review; the transcripts were then reviewed again to identify patterned responses. Supporting and representative quotations were identified after the synthesis of categories of response.

Investigators obtained ethical approval from the University of Washington Human Subjects Division IRB (Committee E/J)and the Mozambican National Committee for Health Bioethics (Reference 47/CNBS/2015).

## Results

In total, 11 FGDs were done with a total of 51 participants (25 male and 26 female): four FGDs with DS-TB (n = 23), three FGDs with MDR/RR-TB (n = 9) and four FGDs with TB-HIV (n = 19) (Table 1). Six discussions were done in Manica (n = 24) and five in Sofala province (n = 27). The discussions lasted between 60–90 minutes.

**Table 1:** Interview characteristics.

	*Male*	*Female*	*Total*
*Drug Sensitive Tuberculosis*	11	12	23
*Multi-Drug Resistant Tuberculosis*	3	6	9
*TB-HIV co-infection*	11	8	19
*Total*	25	26	51

Results were organized around: 1) knowledge; 2) barriers to access services (including treatment); 3) barriers to treatment adherence; and 4) Suggestions for improvement.

### 1. Knowledge of HIV, TB and MDR-TB

Knowledge of TB was less pronounced among all FGD participants. Most referred to TB as a disease transmitted by air. Some respondents in Sofala did not know what TB is and how it is transmitted despite being in treatment for TB.

“*Tuberculosis is a disease of the lungs when the little animals (“Bicho”) start eating*, *you start coughing*, *you can’t eat*, *you lose strength and get very skinny*. *Only when you go to the hospital is when they discover the disease*”

(FGD Nhamatanda (Sofala), DS-TB)

Regarding MDR-TB, knowledge was also limited, although the fact of abandoning treatment was mentioned as a cause of MDR-TB, and most referred to hemoptysis as a sign of MDR-TB. More respondents in Manica province showed good knowledge of MDR-TB. Respondents reported some perceptions of TB and MDR-TB that were related to myths and local traditions or rituals.

“*It’s hard to understand where this disease comes from*, *lots of the times I know it is because of killing a chicken during a funeral*. *It happened to a man; when he arrived from a travel*, *his daughter prepared chicken*, *and before eating*, *he vomited blood and that is how the disease started*.”

(FGD, Nhamatanda (Sofala), DS-TB)

“*The most difficult in understanding TB and MDR-TB is a cough of somebody who abandoned treatment and a bit later the patient is sicker*, *goes to the hospital and has a relapse*, *that is when it is called resistant TB*”

(FGH, Gondola (Manica), TB-HIV)

Participants from all three groups knew what HIV is and how it is transmitted. Participants from Manica had a good knowledge of HIV, while those in Sofala reported questions regarding what HIV is and how it is transmitted.

### 2. Barriers to access TB treatment

Barriers to accessing TB services noted by study participants were mainly related to three aspects: the process of TB diagnosis, health care workers (HCWs) related factors, and individual reasons (Table 2).

**Table 2:** Summary of themes and sub-themes on barriers to access TB-services.

Theme	Sub-theme	Quote
Diagnostic procedures	Long time to get diagnosis	*"I had difficulties because I did analysis twice and the results were negative*. *The third time they sent to the Provincial Hospital of Chimoio*, *I did analysis and it came out positive" (FGD*, *Gondola (Manica)*, *TB-Sens)*
	Receiving empiric treatment before further analysis (taking long time to diagnosis)	*“It takes a lot of time for diagnosis*. *They give us many medications that do not help*, *while we get worse*. *Later*, *after wasting a lot of time*, *we are diagnosed with TB*.*” (FGD*, *Ponta Gea (Sofala)*, *TB-HIV)*
Health Care Worker related factors	Long waiting times	*"There are other sick people who take medication at home*. *This issue on waiting here until 11h shouldn’t happen for a patient" (FGD*, *Eduardo Mondlane (Manica)*, *TB-HIV)*
	Informal fees to get attendance	*"Sometimes when a person who has money and arrives*, *he is attended first" (FGD*, *Eduardo Mondlane (Manica)*, *TB-HIV)*
Individual factors	Bad habits such as smoking, not drinking (can’t keep the advice and are reasons not to start/continue treatment)	*"The problems that patients facing to start treatment is that thing of bad habits*, *such as sexual relationship*, *they shouldn’t eat salt*, *avoid alcoholic drinks" (FGD*, *Gondola (Manica)*, *TB-HIV)*
	Shame of coming to the health center and the stigma of TB	*“Those who do not start treatment is because of their own fault*. *For many it is because of shame to come and receive the pills*, *they are scared to be laughed at by their neighbors*. *Many are scared that people would see that they are under TB treatment*.*” (FGD*, *Nhamatanda (Sofala)*, *MDR-TB)*
	Don’t believe in biomedical understanding of disease—prefer traditional medicine	*"Many don’t trust the treatment at the hospital*. *Many come late because they first go to the traditional healer; only after that they decide to go to the hospital" (FGD*, *Gondola (Manica)*, *TB-Sens)*
	Fear of taking medicines, fear of being hospitalized and dying in the hospital	*"Scared of being hospitalized*, *take medication or to die in the hospital" (FGD*, *Nhamatanda (Sofala)*, *TB-HIV)*

The fact that making a laboratory or clinical diagnosis of tuberculosis is difficult was noted to be a barrier to access treatment. Respondents noted that some initial tests, could be falsely negative, which delayed treatment initiation. Furthermore, several participants noted that the initial (usually empirical) treatment given for regular respiratory tract infections, were not effective. For some people, the absence of seeing improvement resulted in delays in returning to a health facility, or not going back at all.

FGD participants reported that some TB service providers providing preferential services to favored patients, or to those that paid an informal fee despite the fact that TB services should be free. This was reportedly more common in Manica than in Sofala.

Individual reasons as barriers to start treatment were mentioned in both provinces. Reasons mentioned included: preference to see the traditional healer, concern about shame and stigma in the community of being diagnosed with TB, lack of confidence in health system, not wanting/not being able to follow the advices of the HCWs.

Other barriers mentioned were access to laboratory facilities that did TB testing, and to less extent transport and financial difficulties. Some FGD participants said they did not have any difficulty in accessing TB care.

### 3. Barriers to adherence

In general, both in Manica and Sofala, and across all FDGs, participants said that there is a good treatment adherence for TB patients. Barriers to adherence to treatment is detailed in Table 3 and covers aspects on: individual factors; treatment related factors and health care worker related factors.

**Table 3:** Summary of barriers to adherence to TB-treatment.

Theme	Sub-theme	Quote
Individual factors	Difficulties in following advice from health care workers (such as smoking, no sexual activities for 6 months, no alcohol)	*"Others deny because they don’t like to follow the advice*, *they like to drink*. *If they think of drinking*, *they think they loose a lot" (FGD; Nhamatanda (Sofala)*, *TB-HIV)*
	Feeling better so don’t feel the need to continue treatment	*"Many give up when they feel better*, *before the treatment completion" (FGD*, *Gondola (Manica)*, *TB-Sens)*
	Shame of disease (that makes them not go to the hospital); or shame to go back to the hospital when they didn’t follow advice (scared of reaction of health staff)	*"Others don’t continue because of shame" (FGD; Nhamatanda (Sofala)*, *TB-Sens)*
	No trust in health care	*"All people should adhere; some don’t trust the machines in the hospital and like to go to the traditional healer*. *And when they go there*, *they don’t cure*. *Others think the disease is witchcraft" (FGD*, *Nhamatanda (Sofala)*, *TB-MR)*
Treatment related factors	Sides effects as reason to stop the medication (knee pain, loss of appetite, numbness, dizziness, etc.)	*“Some aspects make it difficult to continue*, *the treatment is very strong and provokes pain in the extremities (arms and legs)*.*” (FGD*, *Eduardo Mondlane (Manica)*, *TB/HIV co-infection)*
	Effect of treatment is being hungry and this is a problem if no food at home	*"Others don’t continue because there is no food; the pills provoke hunger and they are very strong" (FGD Nhamatanda (Sofala)*, *TB-Sens)*
Institutional factors	Late attendance	*“The services are good*, *we only complain about the part of the delays in attendance*. *The nurse says we should arrive early and he arrives here very late and that is not acceptable for a sick person*.*” (FGD*, *Eduardo Mondlane (Manica)*, *DS-TB)*

Barriers to adherence noted by FGD participants were primarily related to individual/structural reasons and to the treatment itself.

As individual factors, respondents mentioned shame/stigma, lack prioritization of treatment, or the fact that that participants rapidly felt better and didn’t feel like completing the therapy. Side effects (pain in legs and articulations; itching; weakness and vertigo; swollen feet) were mentioned as a barrier to adherence, particularly in patients with TB/HIV co-infection.

It was mentioned that people don’t return because health care workers do not start their clinic activities on time in the morning.

In lesser extent, hygienic conditions were also mentioned as another negative aspect of the services, such as water cups used for patients to take their medications that were not cleaned from one patient to the next.

### 4. Suggestions for improvement

Suggestions for improvement provided by participants were given on different levels: individual/community; institutional and specifically for HCWs. Most were institutional-level suggestions.

Suggestions included: providing nutritional support for eligible patients; early opening hours of the health facilities; and ensure that HCWs rapidly attend patients in a fair and equitable manner. Patients also recommended improved psychosocial support for TB patients including counseling HCWs to be respectful, to show empathy and to provide more encouragement to patients.

“*Counseling to help the TB patient follow the treatment (is critical)*, *the nurse should strengthen the counseling*, *tell the patient not to smoke*, *not to drink and not to abandon treatment*.”

(FGD, Gondola (Manica), MDR-TB)

Finally, participants requested that facilities provide more sanitary drinking water and clean cups with which they can take their TB medications.

## Discussion

This qualitative study explored knowledge, the quality of TB healthcare service delivery and perceived barriers to access and adherence to treatment among patients infected with DS-TB, MDR-TB and TB/HIV co-infection. We showed that knowledge regarding TB and MDR-TB is lacking, and that the quality of TB services is variable. Barriers to access care were mainly related to the long time to diagnosis and health care worker related, while the main barriers to adherence were individual. The results are in line with similar studies in other Sub-Saharan countries or even in Brazil, where similar barriers were identified[13–15].

Although TB is an “older” disease, surprisingly more is known around HIV and its transmission rate among study participants, including those that were not HIV infected–a testament to the educational, financial and political efforts around HIV nationally. Poor knowledge about TB in the general population has also been described in other countries [16, 17]. This suggests that the NTP should focus on awareness campaigns, perhaps using radio spots and a growing cadre of lay community HCWs or “cough officers” that provide brief talks on TB to patients at health facilities and for families of index TB cases, and then more detailed screening and referral for anyone with symptoms of TB. Perhaps TB educational campaigns could also be twinned with the successful HIV messaging efforts. The NTP has allocated a more significant funding portfolio to these efforts in their current Global Fund award, and this will be focus area in the upcoming NTP strategic plan that is in development for 2019–2023.

A systematic review described similar barriers as we found but cited cost as one of the factors. However, we did not find out of pocket costs as a frequently cited barrier in this particular study [18]. This could be mitigated by the fact that TB services are provide without cost to patients in Mozambique. Of note, this study did not go into detailed analysis of other costs including loss of income from work associated with TB treatment that can lead to catastrophic costs for families. Patients did frequently report lack of food and requested food subsidies which does highlight the degree of poverty and access to basic life necessities in this population.

Stigma continues to be a barrier to accessing and adhering to treatment and is not unique to the sub-Saharan context–a study in Eastern Europe shows that TB is highly stigmatizing [19]. Having TB is often seen as equivalent to the stigma associated with being HIV infected, or even a higher perceived stigma as seen in a study in Southern Mozambique [20]. Increasing and continuous community awareness about this treatable disease is needed, using different channels to reach all levels of the population, such as working with the large numbers of traditional healers to also recognize the symptoms of TB and to refer patients for biomedical evaluation and treatment. Recommendations for TB de-stigmatization have been described and need to be adapted to each country’s context [21].

Diagnostic delays resulted in patients not getting their laboratory TB test results, not returning for their follow-up clinic visit, and at times delaying treatment initiation, as also seen elsewhere [22, 23]. Continuous training of health staff on screening patients for TB and treating patients is necessary and needs to be improved. Interestingly, in South-Mozambique, gaps in knowledge on TB diagnosis and care was described [20], pointing out that continuous training of health care professionals is crucial in order to ensure that patients are screened, diagnosed and treated correctly for TB. Expanded use of improved molecular diagnostics such as the use of Xpert MTB/RIF can contribute to timely and appropriate diagnosis for bacteriologically confirmed TB, and in particular for MDR-TB. However, systems need to be implemented to ensure that all patients with a positive test result, particularly for MDR-TB, are linked to appropriate treatment and follow-up. Interventions such as community outreach to overcome the access barrier has been acceptable in other countries[24]. Mozambique’s national program includes currently TB associated community health care workers activities, where contact tracing and leading facility and community based educational/awareness campaigns are aimed to improve this linkage.

Barriers to treatment adherence vary from individual level factors (such as fear for side effects) to social (mainly stigma) and institutional factors (lack of timely attendance as a principle barrier). Two studies focusing on MDR-TB showed similar barriers to adherence as our study, who used a mixed population of TB patients who were lost to follow-up [11, 25]. Shorter drug regimens and new drug combinations could release the pill burden and its side effects. Adherence counseling and motivational support should be reinforced, and community activities such as home visit to prevent abandonment and strengthened community follow up especially for MDR-TB patients should be explored. Currently the National TB Program in Mozambique is updating a comprehensive psychosocial package and manual to better serve all TB patients.

While overall case notifications for TB are increasing in Mozambique, this study highlights a number of areas identified by patients with TB that need to be improved in order to strengthen the overall TB, TB/HIV and MDR-TB care cascade in Mozambique. In particular, a greater focus on the quality of TB healthcare services is critical, with a patient-centered approach that responds to the needs of patients.

Study limitations are following: Study sites were selected as a convenience sample and may not be representative of Mozambican health facilities. However, results can be used in other contexts of Mozambique with similar socio-cultural norms. Only patients under treatment were enrolled thus we are missing opinions of those did not arrive or abandoned services; health care provider perspectives do not inform study conclusions. While every effort was made to broadly recruit study subjects for interviews, participation at each site was voluntary and guided by facility leadership, which introduces the possibility of bias.

## Conclusions

At study sites in central Mozambique, there is a good knowledge about HIV but not about TB or MDR-TB. Challenges for access and adherence to treatment are mainly at individual and institutional level, but stigma associated with TB and basic lack of knowledge about the disease, treatment and transmission remains a challenge in the community and is preventing some patients from being evaluated and successfully treated. Improved advocacy, de-stigmatization and informational campaigns combined with enhanced screening efforts are necessary to drive testing for TB and to find the missing cases in Mozambique. On the laboratory and clinical side, new diagnostic technologies and shorter more tolerable drug regimens could improve bacteriologically confirmed TB notifications and treatment success rates. Focused adherence counseling sessions and community-based patient centered models for select patients need to be added for the patients at high risk of default and who live significant distances from health facilities. Finally, increased attention to not only coverage and treatment notification, but to the quality of TB healthcare services is necessary to improve TB treatment outcomes. With this in mind, trust between patients and the health care system can be strengthened, responding directly to the needs of the patients.

## Supporting information

S1 File Interview guide. (DOCX)

S2 File COREQ Checklist. (DOC)

S1 Dataset (ZIP)
