# Supplementary material for: Barriers to access and adherence to tuberculosis services, as perceived by patients: A qualitative study in Mozambique
Source: PLoS One. 2019 Jul 10;14(7):e0219470. doi: 10.1371/journal.pone.0219470 (PMC6619801; doi:10.1371/journal.pone.0219470)
Supplement: S1 File — (DOCX) [file pone.0219470.s001.docx]

# Appendix 5.

# UW IRB #48894

# PI: James Cowan

# “*Avaliação da cascata de cuidados de pacientes diagnosticados TB, TB-MR e a Coinfecção de TB-HIV nas Províncias de Manica e Sofala*”

# Instrumento 5: Guião de entrevista de grupo focais para pacientes TB, MDR-TB e TB/HIV

**SECção A: assistência do serviço de saúde às PACIENTES COM TB, MDR-TB e TB/HIV**

1. O que você sabe sobre TB e MDR-TB?
2. O que acha dos serviços sector TB nesta US?
3. Nalgum dia teve qualquer dificuldade durante o processo de aceder os serviços de TB/MDR-TB? Explique.

**SECÇÃO B: ACONSELHAMENTO PÓS-TESTE E ORIENTAÇÃO**

1. Que aprendeu sobre HIV?
2. Que é mais difícil em compreender sobre TB, MDR-TB?
3. Como é que pode ser feito o aconselhamento para ajudar um paciente a aderir ao tratamento TB, MDR-TB o TB/HIV?

**SECção c: Adesão aos serviços HIV/TB/MDR-TB**

*É geralmente difícil para muitas pacientes aderirem às consultas de PNCT e a recomendação ao tratamento TB, MDR-TB e TB/HIV.*

1. Quais são os problemas que enfrenta uma paciente para iniciar tratamento com:
   - 1. HIV?
     2. TB?
     3. MDR-TB?
2. Quais são as aspetos que foram mais difíceis para continuar a fazer tratamento?

**SECÇÃO D: MELHORAR LABORATORIO E PNCT**

1. Existe algo que poderia ser melhorado nos serviços de PNCT?
2. O que deve ser feito pela US seleção ao tratamento e sua continuidade?
3. O que o trabalhador de saúde poderia fazer?
4. Acha que fazer o diagnostico e tratamento imediato da tuberculose melhora o estado de saúde do paciente? (*Sondar: Como? Em qual maneira?)* Explique?
5. Acha que faizes o teste de HIV e inicias TARV melhora a qualidade da vida da paciente? Explique?
6. Tem mais alguma coisa a acrescentar sobre o que já discutimos?

MUITO OBRIGADA [Hora do fim da entrevista ______________]
