# Supplementary material for: Barriers to access and adherence to tuberculosis services, as perceived by patients: A qualitative study in Mozambique
Source: PLoS One. 2019 Jul 10;14(7):e0219470. doi: 10.1371/journal.pone.0219470 (PMC6619801; doi:10.1371/journal.pone.0219470)
Supplement: S2 File — (DOC) [file pone.0219470.s002.doc]

| **COREQ CHECKLIST** | | | |
| --- | --- | --- | --- |
|  |  |  |  |
| **No. Item** | **Guide Questions/Description** | **Reported on page/line #** |  |
| **Domain 1: Research team and flexibility** | | | Location in the Manuscript (Paragraph-section, line)* |
| *Personal Characteristics* | | |  |
| 1. Interviewer/facilitator | Which author/s conducted the interview or focus group? | Felicidade Faria  Georgina Castro  1 collaborator (non-co-author also did data collection) | (authors’ contribution in separate submission, not in main manuscript) |
| 2. Credentials | What were the researcher’s credentials? E.g. PhD, MD | Caroline De Schacht, MD, MSc, PhD |  |
|  |  | Cláudia Mutaquiha, MD |  |
|  |  | Felicidade Faria, Bsc |  |
|  |  | Georgina Castro, BSc |  |
|  |  | Nélia Manaca, PhD |  |
|  |  | Ivan Manhiça, MD, MPh |  |
|  |  | James Cowan, MD, MPH, MBA (PI) |  |
| 3. Occupation | What was their occupation at the time  of the study? | Research Assistants | - |
| 4. Gender | Was the researcher male or female? | 2 female and 1 male | Page 6 |
| 5.Experience and training | What experience or training did the  researcher have? | The members were trained by the PI on the protocol and interview techniques. They were research assistants at time of the study, having experience in the methodology | Page 6 |
| *Relationship with participants* | | |  |
| 6. Relationship established | Was a relationship established prior to study commencement? | No | - |
| 7. Participant knowledge of the interviewer | What did the participants know about the researcher? the researcher? e.g. personal goals, reasons for doing the research | Participant Information sheet, Informed Consent | - |
| 8. Interviewer characteristics | What characteristics were reported about the interviewer/facilitator? e.g. Bias, assumptions, reasons and interests in the research topic | **-** | Page 5 |
| **Domain 2: Study design** |  |  |  |
| *Theoretical framework* |  |  |  |
| 9. Methodological orientation and theory | What methodological orientation was stated to underpin the study? e.g. grounded theory, discourse analysis, ethnography, phenomenology, content analysis | Codes were identified, and organized manually into categories and major themes, using thematic content analysis | Page 6 |
| *Participant selection* |  |  |  |
| 10. Sampling | How were participants selected? e.g.  purposive, convenience, consecutive,  snowball | Participants were selected by purposeful sampling, from the active cohort of patients attending services. All patients were identified at the health facility | Page 5 |
| 11. Method of approach | How were participants approached?  e.g. face-to-face, telephone, mail,  email | Patients were contacted directly at the health centre | Page 5 |
| 12. Sample size | How many participants were in the  study? | A total of 51 participants | Page 7 |
| 13. Non-participation | How many people refused to  participate or dropped out? Reasons? | No refusal rate was seen. | **-** |
| *Setting* |  |  |  |
| 14. Setting of data collection | Where was the data collected? e.g.  home, clinic, workplace | Data were collected in the health facility, in an isolated space and outside of usual working hours | Page 5 |
| 15. Presence of no participants | Was anyone else present besides the  participants and researchers? | No | - |
| 16. Description of sample | What are the important characteristics  of the sample? e.g. demographic  data, date | Characteristics are presented in the table | Page 7 |
| *Data collection* |  |  |  |
| 17. Interview guide | Were questions, prompts, guides  provided by the authors? Was it pilot  tested? | All interviews, both IDI and FGD, were conducted using guides with open-ended questions and probes. The guide was not pilot tested in the field. | Page 6 |
| 18. Repeat interviews | Were repeat inter views carried out? If  yes, how many? | No | - |
| 19. Audial/visual recording | Did the research use audio or visual  recording to collect the data? | No audio or visual records were done. Notes were taken at all discussions | Page 6 |
| 20. Field notes | Were ﬁeld notes made during and/or  after the interview or focus group? | A note taker was always present during IDI and FGD. | Page 6 |
| 21. Duration | What was the duration of the inter  views or focus group? | Each IDI and FGD lasted between 60 and 90 minutes | Page 6 |
| 22. Data saturation | Was data saturation discussed? | Yes | - |
| 23. Transcripts returned | Were transcripts returned to  participants for comment and/or correction? | No | **-** |
| **Domain 3: Analysis and findings** |  |  |  |
| *Data analysis* |  |  |  |
| 24. Number of data coders | How many data coders coded the  data? | Three | Page 6 |
| 25. Description of the coding tree | Did authors provide a description of  the coding tree? | No | **-** |
| 26. Derivation of themes | Were themes identiﬁed in advance or  derived from the data? | Themes were identified in advance | Page 6 |
| 27. Software | What software, if applicable, was used  to manage the data? | Maxqda Version 11 | Page 6 |
| 28. Participant checking | Did participants provide feedback on  the ﬁndings? | No | **-** |
| *Reporting* |  |  |  |
| 29. Quotations presented | Were participant quotations presented  to illustrate the themes/ﬁndings? Was  each quotation identiﬁed? e.g.  participant number | Quotations were presented giving the Identification of the IDI/FGD and sex and age. In case of the FGD was also provided the profile. | Results (pages 8-13) |
| 30. Data and findings consistent | Was there consistency between the  data presented and the ﬁndings? | Yes | **-** |
| 31. Clarity of major themes | Were major themes clearly presented  in the ﬁndings? | Yes | Result section (pages 8-13) |
| 32. Clarity of minor themes | Is there a description of diverse cases  or discussion of minor themes? | Yes | Result section (pages 8-13) |
| ** This information refer to the clean version* | | | |
