# Supplementary material for: Barriers to access and adherence to tuberculosis services, as perceived by patients: A qualitative study in Mozambique
Source: PLoS One. 2019 Jul 10;14(7):e0219470. doi: 10.1371/journal.pone.0219470 (PMC6619801; doi:10.1371/journal.pone.0219470)
Supplement: S1 Dataset — (ZIP) [file pone.0219470.s003.zip › Transcripts TB study/DGF1_.docx]

**"Avaliação da Cascata de Cuidados de Pacientes Diagnosticados com TB, MDR-TB e Paciente Co-infectados com TB/HIV nas Províncias de Manica e Sofalaʺ**

# Instrumento: Guião De Entrevista para Grupos Focais - DGFs

**Data:** 10. 02. 2016

**Distrito:** Beira

**Nome da Unidade Sanitária**: CS Ponta Gêa

**Hora do início:** 08H:08

**Hora do fim:** 10H:18

**Número de DGF:** 01

**Legenda**

**E:** Pergunta do(a) Entrevistador(a)

**P:** Participante/entrevistado(a)

**RP:** Resposta do(a) participante/entrevistado (a)

**PH:** Participante Homem (seguido de sua posição de assento)

**PM:** Participante Mulher (seguida de sua posição de assento)

**n/a:** Não Aplicável

| Comentários/Observações Preliminares: *(circunstâncias que poderão influenciar a entrevista, etc.)* *A DGF correu bem. Tinha seis participantes, todos do sexo masculino.*  *A DGF foi feita por de baixo de uma linda sombra de uma grande e maravilhosa mangueira. Mangueira.* |
| --- |

**SECÇÃO A: ASSISTÊNCIA DO SERVIÇO DE SAÚDE AOS PACIENTES COM TB, MR-TB E TB-HIV**

1. **O que você sabe sobre TB?**

***RP-PH6:*** *TB é uma doença que precisa de muitos cuidados e muita atenção. Se você cumprir o que é dito no hospital, você cura. Se você curar e continuar a fazer o que você fazia antes, vai piorar.*

*Eu estou a medicar a 12 dias, e foi muito dificil detectar a TB em mim. Fiz várias vezes o BK e dava sempre negativo. A médica só descobriu que eu tenho TB através do meu Raio X.*

***RP-PH4:*** *TB é uma doença com tratamento de seis meses, durante esse tempo deve-se tomar os remédios todos os dias. Você pode melhorar enquanto está em tratamento, e muitos nessa fase se dislexam, esses não têm amor a vida. TB tem cura e há momentos que é dificil descobrir. No meu caso, eu fui ao CS de Macurungo, deram-me escarrador e o meu BK deu negativo. Eu ainda continuava a tossir muito, minha mãe me aconselhou a ir em outro hospital, fui ao CS de Ponta-Gêa, e o resultado deu positivo. Daí passei a fazer o tratamento.*

***RP-PH3:*** *TB é uma doença que precisa cuidados. Se a pessoa sabe que tem TB, deve deixar as brincadeiras, deve dormir cedo, comer bem e se comportar bem. Fiz tratamento com uma senhora do Gota, nada mudou. Fui ao hospital, fiz os exames de TB, deu positivo e comecei a fazer o tratamento.*

***RP-PH2:*** *Eu passei muitas fases com dor de barriga e braço, dai fiz o exame de TB, deu positivo, comecei a fazer o tratamento e agora me sinto melhor.*

***RP-PH5:*** *Eu também passei mal, estou a tratar a pouco tempo. TB é uma doença que não se sabe onde está a sair.*

***RP-PH6:*** *A maioria dos doentes tem TB, mas não sabem.*

1. **O que você sabe sobre TB- MR?**

***RP-PH1:*** *TB-MR é recaída, é metade de casa, metade é coisa de serviço. Por exemplo poeira de casa, cimento da obra e outras poeiras, juntando tudo isso dá TB. No hospital obrigam a tomar comprimido, leite, papa, e as vezes não temos e comprimido começa a lavar na barriga. É recaída, muitas pessoas se metem ou dormem na casa de falecido, apanham poeiras e isso é TB. Eu era palito e agora estou a melhorar.*

***RP-PH3*** *Somos dados seis meses para tratar. Você melhora e começa a beber, fumar e brincar mal. Aí você entra em recaida e fica muito mal. Depois de você cumprir a medicaçao deve ficar mais quatro meses sem voltar a fazer o que fazia antes.*

***RP-PH1:*** *A TB-MR até pega pé e você não consegue andar. Para descobrir há-de cair no osso e você começa a acabar.*

***RP-PH6:*** *Quando você não for descoberto nas primeiras semanas, é perigoso. Devemos dar bacela ao tratamento, isso fará com que você não tenha recaidas. A TB dificil de tratar pode ser da segunda via por conta de você voltar aos vicios logo que achar que está bem. Se voce não for esperto a TB pode te gozar e você chega no hospital bem mal.*

1. **O que acha sobre os serviços prestados neste sector de TB?**

***RP-PH3:*** *Tem pessoas que estão um pouco melhor e tem as que estão piores. Esta doença e transmissível, ela parece mensagem. No PNCT tem cinco litros de água e um copo e todos usam o mesmo copo, isto deve melhorar. Temos um atendimento e somos obrigados, e somos também bem aconselhados.*

***RP-PH2:*** *Temos que acordar sempre cedo, temos outras actividades, pedimos para tomar os remédios em casa, e a enfermeira não aceita alegando que esqueceremos de tomar os remédios.*

**E: Mas o que acham dos serviços prestados**

***RP-PH3:*** *Eu acho que é chato tomar os remédios usando o mesmo copo para todos os pacientes.*

***RP-PH6:*** *Os serviços estão bem e a enfermeira nos motiva e dá boas palestras incentivando as pessoas a medicarem.*

***RP-PH4:*** *Os serviços prestados são bons, a enfermeira nos incentiva a vir cedo e a tomar os remédios todos os dias. O galão de 5 litros que contem água está velho, tem partes verdes. Nos incentivam a trazer água, mas há quem esquece. Usam-se os mesmos copos para todos os doentes, isso significa que não vamos e não estamos a melhorar porque a TB está a passar de pessoa para pessoa através dos copos.*

1. **Algum dia teve qualquer dificuldade durante o processo para acesso aos serviços de TB, TB-MR? Explique.**

***RP-PH2:*** *Sim eu tive, por exemplo passei pelo CS de Macurungo, e só me davam receita para tomar medicamentos que não me ajudavam, mas eu já tinha dito que tossia muito e etc. Fiz várias vezes medicações sem necessidade, até que certa vez fui com a minha mãe e a enfermeira pediu que eu fizesse o exame de TB e deu positivo.*

***RP-PH4:*** *Foi muito difícil descobrir a doença. Fui a consulta só me deram cotrimoxazol e tomei esse remedio por muito tempo. Eu sou canalizador, estava a tossir muito e apanhava muita poeira. Fui novamente ao hospital e continuaram a me dar receita e eu sempre dizia que estava a tossir muito e a tirar escarro. Eu sentia frio e calor, comecei a ficar muito mal, me pediram fazer os exames de TB. Comecei em Maio de 2015, e só descobriram a TB em Novembro de 2015. Agora estou em tratamento e estou a me sentir melhor, e vou continuar com o tratamento até ao fim.*

***RP-PH6:*** *Tive dificuldades. Comecei em Novembro de 2015 com tosse. Eu trabalho na Shoprite, fui ao hospital e me deram amoxicilina, mas eu sempre dizia que tossia e que meu corpo aquecia. Tomei 60 comprimidos e nada mudou. Fui novamente ao hospital, dessa vez o da praia nova, fiz teste deu negativo. A diretora do hospital da praia nova me deu documentos para o laboratório, e tudo voltou a dar negativo. Dai tomei 150 comprimidos e nada passou. Voltei a consulta, já aqui na Ponta-Gêa e a enfermeira me mandou fazer o teste de HIV, mas sempre os meus testes de HIV tinham dado negativo. Nesse mesmo dia comprei uma nova senha, um enfermeiro me atendeu e pediu que eu fizesse o Raio X. Levei o Raio X à médica e ela disse que eu tinha TB. Comecei a fazer o tratamento de TB enquanto o laboratório fazia o meu exame de escarro. Fiz mais exames incluindo o de glicémia, e estava tudo bem, mas me aconselharam a não consumir muito açúcar, o laboratório também confirmou o meu resultado positivo de TB.*

***RP-PH3:*** *Eu comecei em Agosto de 2015, fui a consulta, me deram remédios que não davam certo. Voltei a consulta novamente, e o enfermeiro pediu que eu fizesse o exame de TB, que deu positivo e no dia 21 de Janeiro de 2016 comecei com o tratamento de TB.*

1. **O que sabe sobre HIV?**

***RP-PH3:*** *HIV é uma doença que não tem cura, mas se cumprir com o tratamento a pessoa melhora.*

***RP-PH5:*** *HIV vem à razão de TB. Se você cumpriu seis meses de tratamento de TB e apanha muitas recaidas, é sinal que você tem também HIV.*

***RP-PH4:*** *HIV é uma ITS, é um virus. Você apanha HIV através de se meter com uma pessoa que tem HIV. HIV não tem cura, o ARV é bom porque acalma a doença. Os procedimentos para o tratamento de HIV são como os de TB. O tratamento de HIV difere com o de TB porque o tratamento de HIV é eterno e para toda vida. Uma pessoa que faz o TARV fica bem do que uma pessoa que não tem HIV, ela fica bem, fica gorda e bonita. Se não cumprir com o tratamento vai te levar para a cova.*

***RP-PH6:*** *HIV é uma DTS que também se pode transmitir por objectos cortantes. HIV não tem cura, por isso em casa sempre se diz se brincares mal terás HIV. Se cumprires com o tratamento ficarás bem, se não cumprires com o tratamento, o HIV vai te gozar e ficarás bem mal. Muitos que tem TB, as pessoas pensam que eles têm SIDA.*

1. **O que foi mais dificil em compreender sobre TB e TB-MR?**

***RP-PH2:*** *Foi dificil perceber de onde veio a TB.*

***RP-PH1:*** *A TB veio de repente, comecei a tossir, pensei que fosse tosse pequena tipo de bebé. Vim ao hospital, fiz os exames de TB, deu positivo e agora estou a cumprir com o tratamento.*

***RP-PH6:*** *Eu estava a trabalhar quando a tosse começou. Na minha familia ninguém teve TB. Ninguém se quer na minha familia disse que eu poderia ter TB.*

***RP-PH4:*** *Foi dificil de compreender porque todo esse tempo ninguém dizia que eu tinha TB. Eu fui até aos curandeiros, mas nada mudava. Só quando vim à US descobriram a TB.*

***RP-PH3:*** *Foi dificil de compreender, tossia muito e pensei que fosse uma tosse passageira.*

1. **Como é que pode ser feito o aconselhamento para ajudar um paciente a seguir com o tratamento de TB?**

***RP-PH6:*** *Uma coisa importante é isto que estamos a fazer hoje (A DGF). Outra coisa, não é só tratar a TB, mas também dar bons conselhos e nós pacientes pormos em prática. Eu por exemplo, já que vocês estão aqui, gostaria que nos dissessem o que podemos consumir e o que não podemos.*

***RP-PH4:*** *Para o tratamento correr bem também depende de nós. Por exemplo se você leva remédios para casa e não toma, esse é um problema do paciente. As enfermeiras dão bons conselhos, mas alguns não levam a sério.*

***RP-PH2:*** *A enfermeira aconselha a todos. Por exemplo eu me lembro certa vez, logo que saimos de tomar remédios, dois pacientes do nosso grupo passaram a comprar cigarro e puseram-se a fumar. Eu fiquei chocado.*

***RP-PH3:*** *Depende de nós, até a TV aconselha.*

***RP-PH6:*** *Salva quem cumpre. Por exemplo a enfermeira para nos reter, levou nossos cartões.*

***RP-PH1:*** *Devemos cumprir saúde. Devemos tomar comprimidos até seis meses. Se fazer brincadeira há-de ir embora, a enfermeira está a trabalhar para nós.*

***RP-PH2:*** *Esta enfermeira controla bem e é muito atenciosa.*

**SECÇÃO C: ADESÃO AOS SERVIÇOS TB**

*(Geralmente é difícil para muitos pacientes aderirem ao tratamento TB,TB-MR e TB/ HIV).*

1. **Quais são os problemas que os doentes enfrentam para iniciar o tratamento com:**
2. **TB?**

***RP-PH2:*** *Eu não tive problemas. Comecei a tomar os remédios e até hoje tomo sem problemas.*

***RP-PH6:*** *Eu não tive problemas, primeiro fui a consulta, e me deram os remédios.*

***RP-PH4:*** *Eu não tive problemas*

***RP-PH5:*** *Eu não tive problemas. Só o laboratório me deu muito trabalho, andei por oito dias atrás do resultado, eu quase desistia.*

***RP-PH1:*** *No laboratório fui dado escarradores, trouxe os escarradores, fizeram os exames, levei os resultados ao médico e não deu em nada, era negativo. Fui novamente ao laboratório deram-me escarradores e os levei ao laboratório. Esperei três dias e o resultado saiu positivo. Quando me disseram sobre o resultado eu fiquei muito triste e admirado, mas devo cumprir. Me deram três comprimidos e me aconselharam a não beber, não fumar, não mexer mulheres e estou a cumprir. Eu era palito, agora estou bem e costumam me dar remédios para tomar em casa durante o fim-de-semana.*

1. **TB-MR?**

*n/a*

1. **TB- HIV?**

*n/a*

1. **Quais são os aspetos que foram mais difíceis para continuar a fazer o tratamento?**

***RP-PH4:*** *Influência das pessoas, elas dizem estás bem, vamos la brindar com um copo. Sentir-se gordo também pode fazer com que voltemos aos vícios.*

***RP-PH5:*** *Um tcheco (cigarro), meu cunhado está muito mal porque abandonou o tratamento e está com vergonha de ir ao hospital novamente.*

***RP-PH6:*** *Nós devemos pensar que o tratamento é tipo uma esquadra, uma prisão. Se você fugir da prisão e for encontrado vai pagar caro. Isso é o que acontece quando a pessoa abandona o tratamento, você larga tudo, vive a vida e passa a ter recaídas que é muito perigoso. Por exemplo uma vizinha minha de 15 anos tinha TB, fez o tratamento, melhorou, ficou bonita e engordou, mas abandonou o tratamento antes do fim, e agora ela está muito mal e a mãe diz que é SIDA e não está indo ao hospital.*

***RP-PH2:*** *Eu não sei porquê as pessoas abandonam o tratamento. Para mim vou seguir em frente ate ao fim. Por exemplo eu já não escondo que tenho TB.*

***RP-PH1:*** *É cabeça deles, é espirito mau. Eles estão fechados.*

**SECÇÃO D: MELHORAR O LABORATÓRIO E PNCT**

1. **Existe algo que poderia ser melhorado nos serviços de PNCT?**

***RP-PH2:*** *Devem ser melhorados os meios de diagnóstico, leva-se muito tempo para se descobrir que a pessoa tem TB.*

***RP-PH3:*** *Dizem vai e volta na segunda-feira e nesse tempo que estamos em casa era bom que estivéssemos a tomar algo para atenuar os sintomas enquanto esperamos voltar novamente ao hospital.*

***RP-PH4:*** *O laboratório deve melhorar no cumprimento do horário de entrega dos resultados. No PNCT, acho que está tudo bem, e devem ser melhorados os meios de diagnóstico.*

***RP-PH6:*** *Foi difícil descobrir que eu tinha TB, porque o BK que foi usado para o diagnóstico era do modelo antigo. Foi a médica que disse para eu voltar a fazer o BK, usando o modelo moderno*

- 1. **O que deve ser feito pela US na seleção ao tratamento e sua continuidade?**

***RP-PH1:*** *Quando abandonar aqueles bichinhos vão crescer e ficar TB grande. Nascem ovos e se reproduzem e você acaba o corpo.*

**E: Vocês se sentem bem com o tratamento que estão a fazer?**

***RP-PH2:*** *Eu noto uma diferença. Eu estou a sentir-me melhor, embora as vezes tenho me sentido enjoado.*

***RP-PH4:*** *As reações dos medicamentos não são constantes. Elas aparecem, mas logo passam. Por exemplo quando eu matabicho papa, logo em seguida apanho sono. As vezes tenho ficado facilmente nervoso, mas me contenho porque pode ser reação dos medicamentos. Não podemos desistir do tratamento por causa das reações.*

***RP-PH6:*** *Não é muito frequente os doentes desistirem por causa das reações dos medicamentos. As pessoas muitas vezes abandonam o tratamento por estarem a se sentir bem. Certa vez tive que sentar na rua por causa das reações, mas não desisti.*

- 1. **O que o trabalhador de saúde poderia fazer para melhorar aderência ao tratamento?**

***RP-PH4:*** *Os profissionais devem nos proporcionar bom atendimento, muitas das vezes eles nos atendem mal sobretudo quando você não dá refresco.*

***RP-PH6:*** *Os profissionais devem ter muita paciência. Ter paciência e ter amor é muito importante para a satisfação do paciente.*

***RP-PH2:*** *Deve ser melhorado o atendimento.*

**E: O que o profissional devem fazer para aqueles que estão em casa venham ao hospital?**

***RP-PH6:*** *Em relação aos que estão em casa e não querem vir ao hospital, nós só podemos aconselhar a vir ao hospital. Penso que não é tarefa do profissional ir buscar pacientes em suas casas.*

***RP-PH2:*** *Eu consigo notar se uma pessoa tem ou não TB, mas estão por aí e não se preocupam em ir ao hospital e muitos deles passam o dia todo bebendo álcool.*

1. **Acha que fazer o diagnóstico e tratamento imediato da tuberculose melhoraria o estado de saúde do paciente? *(Sondar: como? Ou de que maneira?*)**

***RP-PH4:*** *Sim melhoraria. Quanto mais cedo a pessoa iniciar o tratamento e terminar, é melhor.*

***RP-PH2:*** *Se houvesse um tratamento que levasse menos tempo seria bom porque esse tratamento é muito longo.*

***RP-PH6:*** *Depende do estado em que você vai ao hospital. Se você chega no hospital muito grave, há que haver muita paciência e esforço para melhorar. A pessoa que vem grave terá uma melhoria lenta em relação aos que vêm num estado normal. Por exemplo eu cheguei ao hospital num estado normal, andava e ia ao trabalho.*

- 1. **Acha que fazer o teste de HIV e iniciar o TARV melhoraria o estado da vida do paciente? Explique?**

***RP-PH6:*** *Sim melhoraria. Eu conheço alguém que tem HIV e TB, ele está a fazer os dois tratamentos e está a melhorar.*

***RP-PH4:*** *Sim. Se a pessoa tem TB e depois descobre que tem HIV, é melhor que trate tudo, porque se não fizer ao mesmo tempo uma das doenças vai lhe abater.*

***RP-PH2:*** *Penso que deve fazer os dois tratamentos para ganhar tempo. Imagina se tiver que fazer um tratamento para depois começar o outro, isso levaria muito tempo.*

1. **Tem mais alguma coisa a acrescentar sobre o que já discutimos?**

***RP-PH2:*** *Seria bom que dessem constantemente a papa soja, isso ajudaria muito os doentes.*

***RP-PH6:*** *Há pessoas que tomam o remédio e logo começam as reações e ficam a dormir no hospital até 12h sem comer nada. A papa ajudaria esses pacientes por exemplo.*

**MUITO OBRIGADO (A) Hora do fim da entrevista_10H:18_**
