# Supplementary material for: Barriers to access and adherence to tuberculosis services, as perceived by patients: A qualitative study in Mozambique
Source: PLoS One. 2019 Jul 10;14(7):e0219470. doi: 10.1371/journal.pone.0219470 (PMC6619801; doi:10.1371/journal.pone.0219470)
Supplement: S1 Dataset — (ZIP) [file pone.0219470.s003.zip › Transcripts TB study/DGF-10_.docx]

“**Avaliação da Cascata de Cuidados de Pacientes Diagnosticados com TB, MDR-TB e Paciente Co-infectedos com TB/HIV nas Províncias de Manica e Sofala”**

| **Instumento: Guião De Entrevista para Grupos Focais-DGFs** |
| --- |

**Data:** *24/03/16*

**Distrito:** *Gondola*

**Nome da Unidade Sanitaria:***H.D.Gondola*

**Hora do início:** *09:40*

**Hora do fim:** *11:45*

**Numero de entrevista:** *10*

**Legenda**

**E:** Pergunta do/a Entrevistador/a

**PP:**  Pergunta do/a Participante

**RE:** Resposta do/a Entrevistado/a

| **Comentários/Observações Preliminares:** *(circunstâncias que poderão influenciar a entrevista, etc.)* *comentario a entrevista foi feita numa alprende fechada onde fazen circuzicao masculina com 4 participante no lugar comodo.* |
| --- |

**SECÇÃO A: ASSISTÊNCIA DO SERVIÇO DE SAÚDE AOS PACIENTES COM TB, MR-TB E TB-HIV**

1. **O que você sabe sobre TB?**

**RP-PH4:** *TB é uma doença de tosse que leva duas semanas a tossir, depois de um tempo fui ao hospital fazer analise e acusou TB e aconselharam-me que quando for a tossir deve tapar a boca, e deve tossir dentro de uma latinha e depois tapar com areia.Mesmo em casa deve abrir janelas de casa para entrar ar.*

**RE-PH3**: *TB, é uma doença contajiosa quando estiver a tossir deve ter cuidado com outras pessoas que estiver ai ao lado. Não deve partilhar os utecilio doméstico ,como copo mesmo ao fazer necessidade menor é preciso fazer dentro de buraco.*

**RE-PM1**: *TB é uma doença provocada por um bacilo que pode ser transmitida atravéz do ar, mesmo pizando escaro seco de alguém que tem TB sem saber se pode contaminar .*

**RE-PH2**: *TB é uma doença contagiosa provocada por bacilo e a pessoa tosse muito. Ao tossir deve tapar a boca para não contaminar as outras pessoas pelo ar.*

1. **O que você sabe sobre TB-MR?**

**RE-PH2**: *TB-MR é uma doença que quando uma pessoa que ja tinha TB e depois abandonou o tratamento, depois de um tempo e quando vem no hospital é ja a recaida é quando se chama TB-MR. Isso pelo abandono de tratamento é quando ja é falado de TB resistente e quando chega aqui faz tratamento de injecção para o TB-MR é preciso comprir com tratamento.*

**RP-PM1**: *TB-MR é uma doença de tosse,que até pode tossir sangue. E quando vem aqui no hospital fazer analise é quando sai resultado positivo e tem sintoma de febre,doer dentro do peito até a pessoa tosse sangue. Isso é que se chama TB-MR resistente porque a pessoa fazia tratamento e abandou esse tratamento. quando vai ao hospital fazer analise acusa TB resitente, depois de resultado logo iniciei com tratamento de 6 meses a receber comprimidos.Mas aqui no hopital nós somos aconselhado que olha não deve partilhar utecilio domestico ,deve abrir janela em casa para entrada de ar. Assim ja estou a medicar e ja me sinto melhor com esse tratamento o que ficou são efeitos culaterais desse medicamento é muito forte,mesmo assim ja me sinto melhor e ja faço trabalho de casa enquanto não conseguia fazer nada em casa o problema era sério.*

**RP-PH3**: *TB-MR É aquela tosse em que o paciente não compriu com tratamento todo. Depois de um tempo é quando vem uma recaida ,eu assim tive uma recaida mas ja iniciei com tratamento e tomo 13 comprimido por dia. Um tipo de comprimido comprido, mas a tosse não está a passar estou sempre a tossir não sei porque! mas estou a medicar .*

1. **O que acha sobre os serviços prestados neste sector de TB?**

**RP-PH3**: *Os serviços prestado neste sector de TB estão a andar bem sem problemas, não tenho nada a reclamar. Só o grande problema para mim,é essa tosse que não está a passar até o meu corpo está a ficar fraco por causa de tosse.*

**RP-PM1***: Os serviços prestado estão a andar bem pelo que eu vejo, porque cada doente tem sua maneira de pensar e somos todos dado medicamento. O nosso maior problema são os escarradores para fazer analises, muitas das vezes não existem dentro da unidade sanitária. O grande problema são efeito colaterais e de medicamento até uma pessoa acorda muito fraco.eu passei mal porque muitas vezes que eu fazia analise e não acusava nada sobre a TB, até chegaram a me dar papel de transferência para ir fazer raio X em chimoio, foi quando saiu resultado que o TB esta no osso.Depois de resultado logo iniciou com tratamento e assim estou a ver melhoria porque ha vezes em que não tenho tosse, há vezes em que estou a tossir uma vez por dia e estou registar melhorias.*

**RP-PH4***: Neste sector de TB esta tudo bem, agora ja temos activista que trazem medicamentos nas casa de doentes. Sofri muito para ter o resultado de analise de TB. Cada vez que eu vinha fazer analise não acusava nada ,so a terceira vez, foi quando saiu resultado positivo e logo iniciei com tratamento de TB, assim ja me sinto melhor estou a comprir com tratamento sem falhar. Assim estou a espera para fazer uma nova analise, para ver em que estado de saúde estou agora. Mesmo as activista passam de lá em casa, sempre a visitar e saber de saúde de cada doente lá na comunidade, não temos problema para reclamar.*

**RP-PH2**: *Os serviços neste sector estão andar muito bem, gostaria de ter apoio alimentar porque esse tratamento é muito forte e tomo 12 comprimidos por dia. Agora não é facil sem nenhum apoio alimentar pelo menos receber farinha de soja. Em relação ao tratamento estamos a receber sem nenhum problema, o grande desafio é de fome porque tratamento é muito forte porque a injecção ja terminei agora estou a tomar comprimidos até se você não tem coragem de tomar os medicamento, pode abandonar de tomar esse tratamento.*

**RP-PH3**: *Os serviços estão bem, só esse tratamento é muito forte quando toma nas noite até duas horas madrugada te dão fome, gostaria que mudassem esse tipo de medicamento por exemplo eu estou a tomar mais não estou a ver melhoria,e estou a tosssir sempre.*

1. **Algum dia teve qualquer dificuldade durante o processo para acesso aos serviços de TB, TB-MR? Explique.**

**RP-PH2**: *Sim tive dificuldades, porque quando para ter acesso ao serviço de TB,TB-MR primeiro fui ao hospital fiz consulta e me mandaram ao laboratorio fui dado escarador para por saliva e trazer no dia seguinte. Recebi o resultado positivo de TB. Até algumas pessoas ficaram adimirada que olha teve sorte acusar e iniciar com tratamento de TB. Há pessoas que levam muito tempo a fazer analises mas não sai resultado, Meu resultado saiu e logo iniciei com tratamento. Somos bem atendido só quando fala para enfermeiro que essa nadica esta doer estou pedir para trocar para picar outro lado injecção porque ontem picou esse lado hoje pica outro lado ele responde mal ao doente mais o resto esta tudo bem.*

**RP-PM1**: *Durante o processo para acesso de servico aqui só tive problema no banco de socorro. Mais outros sector não tive problema ,só um dia fomos dado uma ampola para duas pessoas apanhar injeção com mesma ampola.*

**RP-PH4***: Durante o processo não tive dificuldade quanto ao acesso aos serviços porque ja temos activista que andam nas casa a distribuir o medicamento. até fiquei surprendido ao ver activista passar a visitar todos os dias os doentes e preguntar se temos medicamento para tomar porque tomamos 14 comprimidos mas injeção. Porque esse tratamento depende de peso da cada doente, quando chego no banco de socorro fui complicado porque uma ampola serviu para duas pessoas .Dai foi quando ligaram para enfermeiro Alberto e explicou como se pica a injecção. mas nós outros no sector não tive problema.*

**RP-PH3**: *Durante o processo não tive problema ao acesso no serviços de TB.*

1. **O que sabe sobre HIV?**

**RP-PH4**: *HIV é uma doença transmitida atravéz de relação sexual não protegida e HIV tem muita diferença com TB. Mais HIV pode apanhar atravéz de agulha,lamina que usou outra pessoa que tem HIV, para uma pessoa que não tem HIV.*

**RP-PH2*:*** *HIV é uma doença que pode ser transmitida atravéz de relação sexual, laminas nos curandeiros enquanto cortou outra pessoa com HIV, mesmo se tiver uma ferida não pode ter contacto com sangue nessa ferida.*

**RP-PH3**: *HIV é uma doença transmitida através de agulha que usou outra pessoa que tem HIV, pode contrair HIV.*

**RP-PH2**: *HIV é uma doença transmitida através de relação sexual não protegida, objecto cortante como lamina, agulha não pode partilhar com outra pessoa.*

1. **O que foi mais dificel em compreender sobre TB e TB-MR?**

**RP-PH3** *: O Mais dificil em comprender sobre TB e TB-MR é a uma tosse em que uma pessoa abandona o tratamento depois de um tempo fica mais doente vai ao hospital ja é recaida é quando se chama de TB resistente.*

**RP-PH4**: *O mais dificil em comprender sobre TB e TB-MR é que é uma doença em que uma pessoa fazia tratamento e depois abandona medicamento é quando apanha recaida .*

1. **Como é que podo ser feito o aconselhamento para ajudar um paciente com tratamento de TB?**

**RP-PH2***: O ancoselhamento para ajudar a um paciente a seguir com tratamento de TB,as activista deve mobilizar os doentes, fazendo palestras na comunidade. Porque antigamente um doente quando sai-se resultado positivo de HIV,o doente preferia tomar (ratex) medicamento que mata rato para ele morrer porque se não as pessoas vão saber que tenho HIV. É mesma coisa com TB as activista devem forticar com palestra nas comunidade.*

**RP-PH3**: *As activista devem ser muito fortes com a sencibilização lá na comunidade com os doentes ,aconselhar os doentes a não falhar na toma de medicamento, isso deve-se falar para os doentes.*

**RP-PH2**: *As acivista devem aconselhar os doentes de HIV que olha, devem comprir com tratamento, aconselhar não beber, não devem abandonar o tratamento. Fortificar palestra na comunidade, as activistas devem fazer controlo de doente que tomam medicamento .*

**RP-PM1***: O aconselhamento deve ser forte,ter muita mobilização, fortificar o aconselhamento, falar para paciente que olha HIV e TB e malaria estas doencas estão a amatar. Aconselhar se que se tiver filho pequeno deve trazer para fazer teste porque pode contrair a TB porque esta criança está sempre perto deles.*

**RP-PH4**: *As activistas devem fortificar o aconselhamento muita mobilização,aconselhar que não devem abandonar o tratamento, você que ja está em tratamento deve aconselhar quando ver alguém doente encorajar dar moral para tomar medicamnto.*

**SECÇÃO C: ADESÃO AOS SERVIÇOS TB**

*(Geralmente é difícil para muitos pacientes aderirem ao tratamento TB,*

*TB-MR e TB/HIV)*

1. **Quais são os problemas que os doentes enfrentam para iniciar com o tratemnto com:**
2. TB?

**RP-PH2**: *Os problemas que os doentes enfrentam para iniciar com tratamento é essa coisa de ter vícios maus,como relação sexual, não devem comer muito sal,evitar beber bebidas alcolicas. Ha mulheres com agitação de outra amigas dizem que não vou conseguir ficar 6 meses em tratamento sem fazer relação sexual, isso são problema que enfrentamos um doente tanto como homem tanto como mulher aquele que estiver doente naquele momento um dele nao aguenta ficar muito tempo sem fazer relação sexual durante 6 meses em tratamento é uma agitação de amigas lá na comunidade.*

**RP-PH4**: *Os problema que um doente enfrenta na parte de maus vicios, fumar cigarro, beber bebidas alcolicas, não deve comer piripiri.*

**RP-PH3**: *Os problema que enfrentam para iniciar com tratamento de TB é essa coisa de regras do hosital dizer deve ficar 6 meses em tratamento e não fazer relação sexual com varias mulheres.*

**RP-PH2***: Os problema que enfrenta para iniciar com tratamento somos falado para evitar de beber, agora há pacientes que não aguentam evitar .*

1. TB-MR?

*Não aplicavel.*

1. **TB-HIV?**

**RP-PH4**: *TB-HIV o meu ponto de vista TB tem cura, agora HIV não tem cura, só tem tratamento para atenoar o viru, mas se você comprir com esse tratamento melhora mas não cura,e essa pequena diferenca que tem TB-HIV.*

**RP-PH3**: *TB-HIV é que não tem tratamento mas só tem atenoante que pode fazer adormecer o viru de HIV, também pode estar associado com TB, quando é assim primeiro deve tratar TB, depois é quando poder continuar a tomar o tratamento de HIV, e tem pequena diferença que ja ouvi falar que HIV nos outros países ja tem injecção que pode apanhar por ano uma injecção.*

**RP-PH4***: É facil quando um doente comprir com tratamento de TB, porque esse tratamento deve tomar atens de comer nada, Agora tratamento de HIV deve tomar todos os dias e deve comprir com esse tratamento. Mas não é facil comprir com tratamento durante 6 meses . se você comprir melhora. E nós deontes quando estiver melhor devemos ser exemplares para outros doente, dizer que olha eu tinha essa doença mas fiz tratamento e compri ja estou melhor, devemos falar isso para nossos amigos,que estiverem doentes. É normal para um ser humano ter TB, tem tratamento e determinado tempo a medicar e cura. Agora HIV ja é diferente deve sempre tomar medicamento todos os dias até a sua morte*.

1. **Quais são os aspectos que foram mais dificeis para continuar a fazer o tratamento?**

**RP-PH4**: *Aspecto mais dificeis para continuar com tratamento muito mais é essa coisa de enfeito culaterais. Somos aconselhados que não devemos beber não devemos fumar. Por exemplo eu estava mal doente até não conseguia andar uma distância longa nem conseguia nas sobida. Mais com* *esse tratamento ja consigo fazer serviço de casa,o enfermeiro deu me muita força, para não abandonar o tratamento.*

**RP-PH4***: Aspecto mais dificeis para continuar com tratamento é essa coisa de efeitos colaterias porque o tratamento é muito forte.*

**RP-PM1***: Para continuar com trataento é muito dificeis porque esse tratamento é muito forte.Se você não ter coragem de tomar até pode abandonar de tomar por causa de efeitos culaterais, mesmo emfermeiro aconselha-nos que depois de tomar medicamento deve comer alguma coisa.*

**SECÇÃ D: MELHOR O LABORATÓRIO E PNCT**

1. **Existe algo que poderia ser melhorado nos serviços de PNCT?**

**RP-PH3**: *Algo para melhorar nos serviços de PNCT, deveria melhorar essa parte de alimentação,pelo menos que desse farinha de papa soja. Porque esse tratamento provoca muita fome até provoca vertins.*

**RP-PH2***: Nos serviços de PNCT deveria melhorar essa parte alimentar, porque esse medicamento provoca muita forte. Até se você não ter coragem não vai tomar só de pensar a fome e pensar vou comer oque?Pelo menos que desse farinha de papa soja, ia nos ajudar muito com esse tratamento, porque ha outros doente que não tem nada para comer logo de manhã matabicho.Agora com a dosagem de 14 comprimidos é muito pesado. Gostaria que pelo menos 15 em 15 dia recebecemos soja nós os doentes.*

**RP-PM1***: Nos serviços da PNCT deveria melhorar essa parte alimentar,porque esse medicamento é muito forte. Até se você não ter coragem de tomar pode abandonar mesmo, porque você comessa apensar não tenho nada para comer é complicado para doente. A PNCT deve melhorar essa coisa de alimentação.*

1. **O que deve ser feito pela US na selecção ao tratamento e sua continuidade?**

**RP-PH2***: A US na seleção ao tratamento e sua continuidade deve fortificar palestra nas comunidade. Na seleção primeiro deve dar prioridade aquele doente que apanha injecção pelo menos serem eles a ser atendidos 8h, atender aqueles de injeção depois atender aqueles que tomam comprimidos.*

**RP-PH3**: *A US na selecção ao tratamento primeiro deve dar prioridade aqueles paciente que apanha injeção.Mesmo as activista que andam nas casa a distribuir medicamento não devem falar na distribuição se possível deve ser todas segunda feiras,não deve trocar os dias de semana.*

**RP-PH4***: A US com a seleção de tratamento para a sua continuidade não tem problema a unidade sanitaria esta trabalhar bem assim, Só os enfermeiros devem fortificar nas palestras na comunidade mesmo aqui na unidade sanitaria devem fazer palestra para todos os doente.*

1. **O que o trabalhador da saúde poderia fazer para melhorar aderencia ao tratamento?**

**RP-PH3***: O trabalhador de saúde poderia melhorar aderência ao tratamento tendo amor com os paciente, ter paciência com doentes,agora não é que paciente chegou 7:30. fica a espera para ser atendido 10 h,isso não da para um doente.*

**RP-PH2**: *O trabalhador de saúde deve melhorar no atendimento primeiro deve dar prioridade aquele doente de TB,e aquele que apanha injecção deve ser primeiro a ser atendido.Agora eles aqui primeiro dão prioridade os que toma comprimidos e quando terminar aqueles que recebe comprimido é quando, depois vai lá na efermaria ver aqueles doente. é quando vem atender a nos que não apanhamos injecção e nos ficamos cansados de esperar ,mas não são todos enfrmeiro que tem esse comportamento, há dias em que chegamos, encontramos enfermeiro que atende bem que tem paciencia com doente.*

**RP-PH4***: Trabalhador de saúde deve ter amor com doente,ter carinho com doente. Agora essa coisa alimentar um enfeermeiro nada pode fazer talvez projecto é que deve apoiar a US com soja para enfermeiro nos distribuir .Só de lamentar que estamos a pedir que os enfermeiro deve dar prioridade aqueles doentes que apanham injecção,depois é quando pode atender aqueles que tomam comprimidos.*

**RP-PM1**: *Tabalhador de saúde primeiro deve atender bem aos doentes porque há vezes em que atendem mal. Depois um doente fica nervoso porque ficou muito tempo a espera de tratamento depois por causa de fome. Hoje o enfermeiro chegou muito cedo porque eles recomendam a chegar cedo sem comer nada, porque somos aconselhado que devemos sair em casa sem comer nada.*

**RP-PH4**: *Trabalhador de saúde poderia melhorar essa parte de alimentação ,os enfermeiros devem fazer pedido de poio de soja aos projecto que dá soja.Tudo depende de projecto que apoia a saúde,ele sozinho enfermeiro não pode fazer nada.*

1. **Acha que fazer o diagnóstico e tratamento imediato da tuberculose melhoraria o estado de saúde do paciente? (Sondar: como? Ou de que maneira?**

**RP-PH4***: Fazer diagnostico e iniciar com tratamento imediato melhoria o estado do paciente porque não fica muito tempo a espera de tratamento.*

**RP-PH3**: *Fazer diagnostico e tratamento imediato de tuberculose melhoria se ouvesse esse aparelho que faz diagnostico hoje e logo iniciar com trratamento imediato seria tão bom para um paciente não sofrer.*

**RP-PH4**: *Fazer diagnostico e tratamento imediato é bom. Porque o enfermeiro recomenda tomar medicamento antes dee comer nada.*

**RP-PH2**: *Fazer diagnostico e logo iniciar com tratamento é bom, porque melhoraria o estado de saúde de doente, logo tão cedo. Agora essa coisa de activista vem nas casa eu nunca recebi medicamento em casa não tenho activista sempre devo vir aqui apanhar injecção.*

1. **Acha que fazer o teste de HIV e iniciar o TARV melhoraria o estado da vida do paciente? Explique?**

**RP-PH4***: Fazer teste de HIV iniciar com TARV melhoraria o estado de vida de um doente,porque ja não sofre muito tempo ficar a espera de tratamento.*

**RP-PM1***: Fazer teste de HIV e logo iniciar com TARV,melhoria o estado de doente ,quando tem malaria faz analise e logo faz tratamento imediato.Seria bom iniciar com tratamento cedo em vez de ficar muito doente depois e quando vai ao hospital.*

**RP-PH3***: Fazer teste de HIV e iniciar com tratamento logo melhoria o estado do paicente e fazer controlo e receber medicamento para não prejudicar o doente,em vez de ficar muito tempo a espera do tratamento.*

1. **Tem mais alguma coisa a acrescentar sobre o que ja descutimos?**

**RP-PH4**: *Gostaria de acrescentar se houvesse aparelho que deteta logo o TB ossea seria tao bom porque demora muito para acusar.Agora essa coisa de ir a chimoio fazer raio X.*

**RP-PH3***: Nós aqui na US não temos aparelho de raio X um doente deve ir a chimoio para fazer o exame.*

**RP-PH2**: *Gostaria que nós doente tivessemos uma associação de criação de animal para nós não sofrer essa parte de alimentação porque quando fazemos pequeno negocio ja pode ajudar essa parte de alimentação*.

MUITO OBRIGADO (A) Hora do fim da entrevista:*11:45*
