# Supplementary material for: Barriers to access and adherence to tuberculosis services, as perceived by patients: A qualitative study in Mozambique
Source: PLoS One. 2019 Jul 10;14(7):e0219470. doi: 10.1371/journal.pone.0219470 (PMC6619801; doi:10.1371/journal.pone.0219470)
Supplement: S1 Dataset — (ZIP) [file pone.0219470.s003.zip › Transcripts TB study/DGF-11_.docx]

“**Avaliação da Cascata de Cuidados de Pacientes Diagnosticados com TB, MDR-TB e Paciente Co-infectedos com TB/HIV nas Províncias de Manica e Sofala”**

| **Instumento: Guião De Entrevista para Grupos Focais-DGFs** |
| --- |

**Data:***23/03/16*

**Distrito:***Gondola*

**Nome da Unidade Sanitaria:***H.D.Gondola*

**Hora do início:***10:07*

**Hora do fim:***11:26*

**Numero de entrevista:***11*

**Legenda**

**E:** Pergunta do/a Entrevistador/a

**PP:**  Pergunta do/a Participante

**RE:** Resposta do/a Entrevistado/a

| **Comentários/Observações Preliminares:** *(circunstâncias que poderão influenciar a entrevista, etc.)* *Comentario a entrevista foi feita numa alpendrecom 4 participante no lugar comodo no hospital distrital de Gondola.* |
| --- |

**SECÇÃO A: ASSISTÊNCIA DO SERVIÇO DE SAÚDE AOS PACIENTES COM TB, MR-TB E TB-HIV**

1. **O que você sabe sobre TB?**

**RP-PM2:** *TB é uma doença transmitida através de uma cerimonia chamada “phiringanisso”e assim quando morre alguém da familia antes daquela cerimonia tradicionais feita e depois um dos menbro da familia vai fazer relação sexual fora de casa. Mas se tinha usado uma capulana durante a relação sexual lá fora e outra pessoa da familia usar mesma capulana é quando fica doente e quando comessa tossir, tradicionalmente é quando diz que é “phirringanisso”. Porque tinha que ir manter relação sexual depois de cerimonia feitas. Agora quando chega no hospital faz analise é quando diz que é TB.*

**RP-PH4**: *TB é uma doença provocada através de relação sexual pode ser um filho,ou pessoa da familia que fez relação sexual lá fora e chega em casa, cuzinha poe sal na comida que preparou para a familia isso pode causar TB, primeiro antes de ir a casa deve tratar tradicionalmente porque andou fora de casa enquanto tinha faleciemento na familia e era antes de ser feitas as cerimonias tradicionais.*

**RP-PM1**: *TB é uma doença que vem por causa de mexer panelas, mexer onde fica farinha, enquanto fez relação sexual, com alguém e não se tratamentou tradicionalmente isso provoca TB enquanto na familia havia falecimento*

1. **O que você sabe sobre TB-MR?**

**RP-PM2**: *TB-MR é chamado por “Tsanganico” vem através de uma cerimonia não tratada de um falecimento da familia. Depois de um tempo é quando fica doente e vai ao curandeiro é quando diz que é “Tsanganico” começa emagrecer tossir sângue.*

**RP-PH3***: TB-MR é “Tsanganico”vem através de uma cerimonia não feita do menbro da familia que perdeu a vida e depois um dos menbro da familia vai fazer relação sexual antes de cerimonia ser tratada ,pode acusar TB-MR, mas aqui no hospital foi dado um escarador e fez analises e acusou TB-MR . Assim uma pessoa com escaro ao tossir deve tapar porque pode alguém pisar aquele escaro e contrai TB de outra pessoa.*

1. **O que acha sobre os serviços prestados neste sector de TB?**

**RP-PH3***: Os serviços prestado no sector de TB todos estão bem,quando chegamos aqui no hospital somos muito bem atendidos e somos tratado. se é comprimido é dado sem problema se é injecção apanha e vai a casa. Mesmo nos dias de feriado somos tratados. somos dado uma ampola para apanhar injecção no banco de socorro.*

**RP-PM1*:*** *Os serviços neste sector de TB esta tudo bem, quando chegamos aqui logo somos atendido sem problema apanhamos injecção.Mas se você comprir com esse tratamento você melhora a sua saúde*.

**RP-PM4**: *O serviço esta muito bem logo que chegamos aqui somos atendido sem problema ,e se você comprir com esse tratamento melhora a sua saúde.*

1. **Algum dia teve qualquer dificuldade durante o processo para acesso aos serviços de TB, TB-MR? Explique.**

**RP-PH3***: Durante o processo para acesso aos serviços de TB nunca tive dificuldade,primeiro fui ao laboratorio e depois de laboratorio saiu resultado positivo, fui mandado para PNCT e logo iniciei com tratamento. Mas primeiro tratamento foi de injecção durante 2 meses e depois fiz outra analise para ver o estado de saúde se ainda continua a doença ou não.Mas como não melhorou estou ainda apanhar tratamento de injeção*.

**RP-PM2**: *Não tive dificuldade durante ao acesso aos serviços primeiro fui ao laboratorio fazer analise,depois de resultado BK+ logo iniciei com tratamento de TB sem problema até hoje não tenho problema com tratamento .*

**RP-PH4**: *Durante o processo não tive problema primeiro fui ao laboratorio fazer analise depois de resultado positivo é quando iniciei com tratamento.*

1. **O que sabe sobre HIV?**

**RP-PH4**: *HIV é uma doença que pode apanhar através de objecto cortante como lamina,que usou uma pessoa com HIV,relação sexual não protegida com uma pessoas que tem HIV.*

**RP-PM1***: HIV é uma doença contagiosa provocada atraves de objecto cortante lamina que usou uma pessoa com HIV*.

**RP-PH3***: HIV é uma doença contagiosa que vem através de relacção sexual não protegida outra pessoa falam de que são esse estrageiros que trouce essa deonçaa de HIV ,porque eles fazem relação sexual com cães e são pago dinheiro.*

**RP-PM1***: HIV é uma doença contagiosa que aprece no corpo através de relacção sexual não protegida.*

1. **O que foi mais dificel em compreender sobre TB e TB-MR?**

**RP-PM2:** *Sobre TB,TB-MR é tsanganico quando uma pessoa faz relação sexual com uma pessoa que enquanto está nas cerimonia de um falecimento de uma familia isso causa tsanganco*.

1. **Como é que podo ser feito o aconselhamento para ajudar um paciente com tratamento de TB?**

**RP-PH4**: *O aconselhamento para ajudar um doente com TB deve ser fortificado e dizer que olha não deve comer muito sal, não deve ir ao curandeiro enquanto esta em tratamento daqui do hospital,não deve beber,não deve fumar não fazer relação sexual. Quando chegamos aqui no hospital fazemos tratamento e somos aconselhados isso tudo que acabai de falar agora,mesmo quando falamos de vir aqui no hosital o enfermeiro tem dado aconselhamento, até há vezes em que o enfermeiro percegue nas nossas casas manda as activista para nos aconselhar porque ja não vem ao hospital.*

**RP-PM1**: *Aconselhamento para ajudar um paciente a seguir com tratamento de TB, o enfermeiro deve fortificar aconselhamento para doente.Que olha não deve fumar cigarro, não deve fazer trabalho forte e deve comprir com tratamento.*

**RP-PH3**: *Aconselhamento para ajudar um doente de TB é seguir com tratamento ,o enfermeiro deve fortificar o aconselhamento falar para paciente não deve fumar ,não deve beber e não deve abandonar o tratamento*.

**SECÇÃO C: ADESÃO AOS SERVIÇOS TB**

**(Geralmente é difícil para muitos pacientes aderirem ao tratamento TB,**

**TB-MR e TB/HIV)**

1. **Quais são os problemas que os doentes enfrentam para iniciar com o tratemnto com:**
2. **TB?**

**RP-PM2***: Os problema que os doentes enfrentam para iniciar com tratamento aqui nesse hospital não temos problema somos bem atendido,não temos nada a reclamar*.

**RP-PH3***: Os problemas que os doentes enfretam para iniciar com tratamento nunca teve problema tudo esta bem, quanto ao inicio de tratamento de TB mesmo aqueles que apanham injecção não tem problema, temos tido um bom atendimento.*

**RP-PM2**: *Não temos problema somos bem atendidos.*

**RP-PH3**: *Não temos problema quanto ao iniciar do tratamento há vezes em que há troca do medicamento, mas mesmo assim tem explicado ao paciente porque é que troca medicamento até da moral de vir aqui no hospital.*

**RP-PH4**: *Eu nunca enfrentei nenhum problema tudo foi bom quando cheguei aqui fui bem atendido e logo iniciei com tratamento imediato.*

**RP-PH3**: *Há outros doentes que vem receber medicamento quando chegam em casa ja não tomam esse medicamento. Não querem deixar de fumar .Agora quando apanha recaida ja não quer ir ao hospital por causa de vergonha*.

1. **TB-MR?**

**RP-PH3**: *TB-MR é quando você apanha recaida. Mas se você comprir com tratamento atè melhora sua saúde, só de lamentar que esse medicamento é muito forte e deve-se tomar antes de comer nada mesmo assim passamos muito mal de fome, até pode não conseguir andar ,há vezes em que sinto muito sono será que são efeitos colaterais desse tratamento? Até se você não ter coragem pode não tomar mais esse medicamento, por causa desses efeito colaterais desse medicamento.*

**RP-PM2**: *TB-MR é quando uma pessoa tem TB depois não compriu com tratamento,é quando aparece TB-MR mas se você comprir com tratamento melhora o estado de saúde mas se você não ter coragem de tomar até pode abandonar por causa de fome.*

1. **TB-HIV?**

*Não aplicavel.*

1. **Quais são os aspectos que foram mais dificeis para continuar a fazer o tratamento?**

**RP-PM2**: *Aspecto mas dificeis para continuar com tratamento é essa coisa de efeito colaterais porque esse medicamento provoca muita fome, até se você não ter coragem de tomar pode abandonar esse tratamento porque é muito forte esse meicamennto . Agora se nao tomar não esta prejudicar enfermeiro, estara a prejudicar-se a si mesmo.*

**RP-PH3***: O mais dificil para continuar com esse tratamento, é nós tomarmos 14 comprimidos por dia, quando terminar começa com injecção é preciso aguentar, porque é preciso vir aqui todos os dias apanhar injecção. O tratamento é muito forte provoca muita fome, e deve-se tomar antes de comer nada é por isso que não temos coragem até pode abandonar porque há vezes em quenvocê em casa não tem nada para comer começa a pensar que oquê vou comer Conheço duas pessoas que abandonaram tratamento por caua de fome,mas se você comprir melhora a sua saúde.*

**RP-PM1**: *O mas dificil para continuar a fazer tratamento é por causa de fome, porque esse tratamento é muito forte até se não ter coragem pode abandonar. Agora há outros doentes que tem ajuda de comida e recebem soja, o mesmo doente em vez dele comer vai fazer troca com bebida (nipa) bebida seca para ele beber*.

**RP-PH3**: *Para continuar a fazer tratamento é por causa desse tratamento que somos dado antes de tomar esse tratamento somos falado muitas coisas para deixar de fazer, não fazer relação sexual enquanto está em tratamento. Agora muitos doentes depois de uma semana em tratamento quando vver que ja se sente melhor, comessa a fazer relação sexual, e diz que está melhor mas isso não é bom prejudica a sua saúde.*

**SECÇÃ D: MELHOR O LABORATÓRIO E PNCT**

1. **Existe algo que poderia ser melhorado nos serviços de PNCT?**

**RP-PH3***: Sim poderia melhorar essa parte de alimantação com ajuda de farinha de papa soja, para pelo menos fazer papa soja porque esse medicamento é muito forte.*

**RP-PM2***: Poderia melhorar o serviço de PNCT com apoio de farinha de papa soja,porque esse medicamento é muito forte e provoca muita fome,poderia melhorar essa parte de alimentação.*

**RP-PH4*:*** *Poderia melhorar essa coisa que os colega acabaram de falar ,nós ajudar com farinha de papa soja.Porque esse tratamento provoca muita fome.*

1. **O que deve ser feito pela US na selecção ao tratamento e sua continuidade?**

**RP-PH4**: *A US na seleção ao tratamento deve reforçar essa parte de aonselhamento para os doentes não abandonar tratamento.Os trabalhadores da saúde devem ir na comunidade fazer palestra, dar moral ao doente, seria bom para doentes que estão la na comunidade, que não tem comunicação.*

**RP-PH3***: Os enfermeiros na seleção ao tratamento para a continuidade deve ir na comunidade para dar palestra, mas em todo caso as activista andam de casa em casa a dar medicamento aos doentes nas zonas isso facilita os doente.*

**RP-PH4**: *As activistas quando é tratamento de HIV é quando andam de casa em casa a dar medicamento,aos doentes que tem HIV,isso poderia acontecer connosco também doente de TB. Porque tem doente que abandonam tratamento por causa de não conseguirem chegar aqui no hospital,buscar seu tratamento se fizessem também com doentes de TB seria bom para um doente.*

1. **O que o trabalhador da saúde poderia fazer para melhorar aderencia ao tratamento?**

**RP-PH3***: O trabalhador de saúde poderia melhorar essa parte, deve dar moral ao doentes,ter um bom tratamento,boa comunicação com doentes para ter mais aderência no tratamento.*

**RP-PH4**: *O trabalhador de saúde deve dar moral aos doentes.*

**RP-PM1**:*O trabalhador de saúde deve dar moral aos doentes, dar aconselhamento que olha não deve abandonar tratamento.*

1. **Acha que fazer o diagnóstico e tratamento imediato da tuberculose melhoraria o estado de saúde do paciente? (Sondar: como? Ou de que maneira?**

**RP-PH3***: Seria tão bom fazer dignostico e tratamento de imediato de tuberculose,melhoraria o estado de saúde do doente, porque o doente não ia sofrer até chegar no ponto de ir a chimoio fazer analise é porque estava a procura de diagnostico*.

**RP-PM1**: *Seria bom para paciente fazer diagnostico e tratamento imediato do tuberculose,melhoraria e o estado de saúde de um doente em vez de ficar muito tempo doente a espera do diagnostico*.

1. **Acha que fazer o teste de HIV e iniciar o TARV melhoraria o estado da vida do paciente? Explique?**

**RP-PM2**: *Seria bom fazer teste de HIV e iniciar com tratamento de TARV melhoraria o estado de vida de um doente não ia ter muitos problemas a espera de iniar o TARV, é bom para estado de saúde de doente*.

**RP-PH3**: *Seria tão bom iniciar o TARV cedo isto melhoria o estado de vida do doente, quanto mas cedo iniciar com tratamento. porque ajudaria a saúde de um doente em vez de ficar muito tempo a espera de tratamento depois de estar muito mal é quando vai ao hospital ,sera dificil para melhorar a saúde de um paicente .*

1. **Tem mais alguma coisa a acrescentar sobre o que ja descutimos?**

**RP-PH3***: Gostaria de acrescentar que o hospital deve distrbuir farinha de papa soja para doente porque esse tratamento é muito forte precisa de comer depois de tomar medicamnto.*

**RP-PM2**: *Gostaria que a o hospital distribuisse farinha de papa soja para doente de TB porque esse tratamento é muito forte.*

MUITO OBRIGADO (A) Hora do fim da entrevista:*11:26*
