# Supplementary material for: Barriers to access and adherence to tuberculosis services, as perceived by patients: A qualitative study in Mozambique
Source: PLoS One. 2019 Jul 10;14(7):e0219470. doi: 10.1371/journal.pone.0219470 (PMC6619801; doi:10.1371/journal.pone.0219470)
Supplement: S1 Dataset — (ZIP) [file pone.0219470.s003.zip › Transcripts TB study/DGF2_.docx]

**"Avaliação da Cascata de Cuidados de Pacientes Diagnosticados com TB, MDR-TB e Paciente Co-infectados com TB/HIV nas Províncias de Manica e Sofalaʺ**

# Instrumento: Guião De Entrevista para Grupos Focais - DGFs

**Data:** 11.02.2016

**Distrito:** Beira

**Nome da Unidade Sanitária**: Centro de Saúde da Ponta-Gêa

**Hora do início:** 08H:10

**Hora do fim:** 10H:06

**Número de DGF:** 02

**Legenda**

**E:** Pergunta do(a) Entrevistador(a)

**P:** Participante/entrevistado(a)

**RP:** Resposta do(a) Participante/entrevistado(a)

**PH:** Participante Homem (seguido de sua posição de assento)

**PM:** Participante Mulher (seguida de sua posição de assento)

**n/a :** Não Aplicável

| Comentários/Observações Preliminares: *(circunstâncias que poderão influenciar a entrevista, etc.)* *A DGF correu bem. Tinha seis participantes, dois eram do sexo masculino e quatro do sexo feminino. A DGF foi feita por de baixo de uma linda sombra de uma grande e maravilhosa mangueira.* |
| --- |

**SECÇÃO A: ASSISTÊNCIA DO SERVIÇO DE SAÚDE AOS PACIENTES COM TB, MR-TB E TB-HIV**

1. **O que você sabe sobre TB?**

***RP-PH6:*** *TB pode ser uma simples tosse, isso é o que as pessoas pensam. Com o tempo você descobre que essa tosse pode não ser normal. Eu tossi por mais de duas semanas, fiz o exame, deu positivo e comecei o tratamento.*

***RP-PH3:*** *Eu comecei por sentir que tinha abscessos, fui ao hospital deram-me remédios e nada passou. Obrigaram-me a fazer exames e acusou TB.*

***RP-PM2:*** *Comecei a sentir pontadas, fiquei internada, saí, fui ao são lucas onde me levaram ao Centro de Saúde da Ponta-Gêa, fiz análises e deu TB positivo e comessei a fazer o tratamento mais levei quase um mês para iniciar o tratamento.* ***RP-PM1****:*

*Comecei com sintomas de tosse, tomei vários remedios e não passava. Meu tio levou-me a marista, fui ao HCB fiz lá o Raio X, depois vim a Ponta-Gêa onde descobriram a TB. Agora estou a medicar. Eu sentia muito frio e o corpo aquecia.*

***RP-PM5:*** *Comecei a sentir frio e o corpo aquecia. Falei com minha prima, fui ao hospital e acusou TB.*

***RP-PM4:*** *Eu não tinha apetite de comer, só bebia muita água e urinava muito*

1. **O que você sabe sobre TB- MR?**

***RP-PH6:*** *Para originar a TB-MR (recaída) é porque nós doentes violamos o tratamento. Muitos por exemplo bebem álcool enquanto estão em tratamento o que leva a resistência da cura e a recaída. As consequências disto são graves.*

***RP-PH3:*** *A alimentação pode levar a recaída. O remédio é muito forte, exige comer muito e bem. Também não se podem perder noites.*

***RP-PM4:*** *Se a pessoa não toma comprimidos pode levar a recaídas.*

***RP-PM1:*** *Fumar, perder noites e não tomar os remédios leva a recaída*.

1. **O que acha sobre os serviços prestados neste sector de TB?**

***RP-PH3:*** *Por enquanto sentimos que estão a trabalhar bem. O problema penso que é nosso. Há pessoas que levam remédios e não tomam, sendo assim não melhoram e passam a falar mal da saúde cada pessoa pensa da sua maneira.*

***RP-PM2:*** *Mesmo sabendo que estão a trabalhar mal eu sempre venho no hospital e*  *estou sendo bem atendida.*

***RP-PM1:*** *não tenho razão de queixas, os enfermeiros estão a trabalhar bem mesmo* *Eu também estou sendo bem atendida.*

***RP-PH3:*** *Todos os hospitais atendem bem. Nós somos complicados, temos de saber porquê estamos indo ao hospital, muitos doentes não obedecem com as regras do hospital, porque devemos saber quando vamos ao hospital e porquê vamos.*

***RP-PH6:*** *De um(1) a dez (10) eu dou sete valores.*

***RP-PH3:*** *O recipiente de água deve ser mudado, tem uma camada que não está boa.*

***RP-PH6:*** *Acrescentando e continuando, sinto que há profissionais que não falam bem. Eles gritam, isso não está certo. Devem falar com respeito e suavidade. Gostaria que melhorassem a linguagem. O horário não está certo eles começam a atender muito tarde, nos precisamos levar os remédios cedo para que possamos ir ao trabalho. Mesmo que fosse para levar os remédios as 06H nós viríamos. Os patrões moçambicanos são complicados não toleram atrasos. Eu comecei a medicação no dia 07.01.2016, peço que melhorem o cumprimento do horário.*

***RP-PM1:*** *Eu me sinto bem, me atendem bem.*

***RP-PM2:*** *Eu estou sendo bem atendida, desde que comecei nunca fui mal atendida, mesmo que alguns cheguem tarde são também bem atendidos.*

***PR-PM5:*** *Não estou a ver mal deles.*

***RP-PH3:*** *A nossa enfermeira berra muito sempre que algum paciente não responde a primeira que é chamado.*

***RP-PM4:*** *Já tenho dois meses a tratar. Sinto que estão a atender bem. Eu não conseguia andar, vinha de tchopela e agora já consigo andar. O comportamento está bom por parte dos profissionais.*

1. **Algum dia teve qualquer dificuldade durante o processo para acesso aos serviços de TB, TB-MR? Explique.**

***RP-PH3:*** *Leva-se muito tempo para o diagnóstico. Dão-nos muitos medicamentos que não ajudam, enquanto vamos piorando. Mais tarde depois de se perder muito tempo é que somos diagnosticados TB.*

***RP-PH6:*** *Para mim não custou nada, mas tenho que obedecer questões próprias. Fui ao hospital, fiz o Raio X, depois fui fazer o exame de TB. Gostei do pessoal do* *laboratório, que até me ligaram para me dizer que devia ir buscar o resultado. O resultado foi positivo, estou a tratar e acho que para mim foi fácil.*

***RP-PM4:*** *Sentia meu corpo muito fraco e estava sempre a dormir. No hospital me deram frasco, levei a Ponta-Gea e aqui descobriram que eu tinha TB***.**

**E: Não teve dificuldades para iniciar o tratamento?**

***RP-PM4:*** *Primeiro fiz consulta, fiz o Raio X e vim deixar no hospital, dai descobriu-se que eu tinha TB.*

***RP-PM5:*** *Eu fui entregue o frasco, fiz Raio X, descobriram a doença e comecei a tomar os medicamentos.*

***RP-PM2:*** *Eu comecei com pontadas, vómitos e malária. Eu não conseguia nem ficar de pé. Fui levada ao HCB, a tensão estava muito alta e fui internada por uma semana. Fiz o Raio X. Depois de me darem alta a médica me deu um documento para o CS. Fui ao CS, fiz consulta me deram cotrimoxazol e outros remédios, mas mesmo assim a tosse não passou. Dai me levaram a Ponta-Gêa onde descobriram a doença e comecei imediatamente a fazer o tratamento.*

***RP-PM1:*** *Eu passei muito mal. Fiquei muito tempo doente. Tomei vários remédios, só mais tarde é quando descobriram que tinha TB. No dia 20 de Janeiro de 2016 comecei a tomar os remédios. Quando tomo os remédios sinto muita comichão, assim já começou.*

1. **O que sabe sobre HIV?**

***RP-PM2:*** *Eu não sei o que é HIV. Lá no hospital tiram sangue, fazem análise e dizem que é seropositivo. No meu caso também me tiraram sangue e me disseram que sou seropositiva e deve aceitar a medicação lá onde apanha o tratamento.*

***RP-PM1:*** *Eu não sei o que HIV, só costumo ouvir falar.*

***RP-PH6:*** *Eu em brincadeira digo que é uma associação desportiva de muitas doenças. O HIV cria deficiência do nosso organismo, neutraliza os anticorpos e nos debilita, tudo isso através de várias doenças.*

***RP-PH3:*** *HIV é uma doença associada de muitas doenças. Dizem que se apanha pelas lâminas e outros objetos cortantes. A doença tem tratamento, é importante seguir a medicação para ser salvo.*

***RP-PM5:*** *Eu não sei o que é HIV, só costumo ouvir SIDA, SIDA só m,as não entendo de onde vem a doença.*

***RP-PM4:*** *Eu não sei o que é HIV, alias sei, mas não sei explicar. Se apanha com lâminas e ir no curandeiro.*

***RP-PH3:*** *Aqui no Hospital fazem palestras de HIV. Ja houvi falar sobre esta doença mas não sei como se manifesta, porque não tem boa explicação.*

1. **O que foi mais dificil em compreender sobre TB e TB-MR?**

*Ninguém respondeu a esta questão.*

1. **Como é que pode ser feito o aconselhamento para ajudar um paciente a seguir com o tratamento de TB?**

***RP-PH3:*** *Depende do pensamento de cada doente. Há pessoas que têm vergonha de serem vistas. Outros levam remédios e não tomam, temos de cumprir para melhorar. Penso que dando soja como incentivo os doentes poderiam vir com mais afluência.*

***RP-PH6:*** *Eu acho que devem haver psicólogos que só lidam com esse tipo de doentes. O enfermeiro não dá conta do recado, porque ele deve atender e aconselhar. Também deveria se fazer uma entrevista aos doentes para se saber sobre sua vida socio-económica, de modo a ajudar este doente caso precise. As ONGs poderiam ajudar dando um cabaz aos doentes mais necessitados. Uma das coisas que o MISAU está a falhar é não contratar um psicólogos para trabalhar especificamente no convencer o doente a medicar.*

***RP-PM2:*** *Os remédios provocam muita fome, mas queremos saúde.*

***RP-PM5:*** *Os remédios são muito fortes, deve-se comer muito.*

***RP-PM4:*** *É a mesma coisa que falou a colega*

**SECÇÃO C: ADESÃO AOS SERVIÇOS TB**

***(Geralmente é difícil para muitos pacientes aderirem ao tratamento TB,TB-MR e TB/ HIV).***

1. **Quais são os problemas que os doentes enfrentam para iniciar o tratamento com:**
2. **TB?**

***RP-PH6:*** *Desde a consulta até ao laboratório foi tudo flexível. Até fui ligado para ir buscar o meu resultado.*

***RP-PH3:*** *Como somos africanos as vezes nos baseamos na tradição e nos esquecemos do hospital. Se formos ao hospital vamos ficar bem. Se tu ficares na tradição vais morrer.*

***E:*** *E a medicação constitui problema?*

***RP-PH3:*** *Quando você está muito acabado o medicamento e um problema. Você deve ser forte para tomar o remédio, comer e fazer outras atividades.*

***RP-PH2:*** *O curandeiro faz suas magias, quer galinha e sei lá o quê. Enquanto se fores ao hospital vais melhorar.*

1. **TB-MR?**

*n/a*

1. **TB- HIV?**

***RP-PM2:*** *Eu não sei se estou a tomar ARVs, mas agora estou somente a fazer o tratamento de TB. Eu comecei o tratamento de TB no dia 05.*

***RP-PM1:*** *Eu estou somente a fazer o tratamento de TB.*

***RP-PM4:*** *Eu estou a fazer ARVs e tratamento de TB. Comecei no ano passado, tenho três crianças e são saudáveis. Os ARVs são muito fortes, no ano passado me causavam vertigem, e eu parecia maluca. Certa vez as pessoas vieram me assistir, depois o enfermeiro disse que era efeito dos ARVs. O comprimido de TB leva a muita fome.*

***RP-PH6:*** *Eu penso que precisar-se-á fazer o CD4 antes de começar o TARV*

1. **Quais são os aspectos que foram mais difíceis para continuar a fazer o tratamento?**

***RP-PH3:*** *É a vontade do doente. Ele deve ter vontade de vir ao hospital.*

***RP-PM2:*** *O problema é dos pés, eles doem muito e aquecem como se a pessoa tivesse levado chamboco nos pés.*

***RP-PM1:*** *Os pés doem, quando você pede alguém ir buscar remédios, as vezes negam. O corpo fica mal, os pés aquecem, os joelhos doem, até perdemos vontade de comer.*

***RP-PH6:*** *Devido a fraqueza, má alimentação do paciente, e por aspetos psicológicos as pessoas simplesmente relegam a sua saúde por simples preguiça mental.*

***RP-PM4:*** *É não vir no hospital receber comprimidos. Outros é por preguiça.*

**SECÇÃO D: MELHORAR O LABORATÓRIO E PNCT**

1. **Existe algo que poderia ser melhorado nos serviços de PNCT?**

***RP-PM2:*** *Os copos são os mesmos para todos. A maioria de nós vem com sua água, mas ainda há pessoas que usam a água e copos do hospital.*

***RP-PM1:*** *Nós doentes somos complicados. Pediram que trouxéssemos água mas nem todos trazem.*

***RP-PH6:*** *Deve melhorar a comunicação. Devem haver modos corretos de falar com os doentes, sobretudo para os pacientes graves. Penso que o MISAU deve pôr baldes bonitos com torneira e deve ser mantida uma higiene exigente. A água que está aqui no hospital tem algas, isso é caricato e inadmissível. O hospital deve ser um exemplo de boa higiene. Foi vergonhoso ver uma enfermeira a fazer o serviço de servente. Mais uma vez digo que é bom que existam psicólogos não só para os doentes, mas também para os profissionais para que estes aprendam a atender os pacientes corretamente. Quando o profissional atende bem, o doente fica motivado e confortável.*

- 1. **O que deve ser feito pela US na selecção ao tratamento e sua continuidade?**

***RP-PH3:*** *Considerar o doente, dialogar corretamente com o doente tirando e esclarecendo suas dúvidas. O sector está bom. Tem um enfermeiro muito bom aqui, se o CS tivesse dez como ele, seria um ganho. Não há descriminação, penso que podemos estar todos juntos.*

**E: Fazendo os tratamentos de TB e HIV, quais são os efeitos?**

***RP-PM4:*** *O tratamento está bom porque agora já não sinto os efeitos dos remédios.*

***RP-PH3:*** *Quando alguém está doente deve cá vir para melhorar.*

***RP-PH6:*** *Eu penso que isso é uma questão técnica, se dependesse do doente talvez faríamos um tratamento até terminar para depois começar com o outro.*

- 1. **O que o trabalhador de saúde poderia fazer para melhorar aderência ao tratamento?**

***RP-PH3:*** *Primeiro ponto é carinho, boas respostas e encaminhar o doente de boa maneira.*

***RP-PH6:*** *Cada profissional ao chegar no seu trabalho deve saber com clareza o que vem fazer e trabalhar de forma correta. O doente se alegra quando é bem tratado e quando é tratado a tempo. O enfermeiro deve ter a capacidade suficiente de esclarecer as coisas aos doentes. Peço que sejam mais flexíveis ao dar medicamentos. O tempo de espera do doente deve ser reduzido, devem ser priorizados os mais velhos e os mais graves. Há um grande aglomerado de doentes nos sectores.*

***RP-PM1:*** *Devem nos atender com respeito, como pessoas, é o que nós queremos.*

***RP-PH6:*** *Aqui no quintal do hospital há muita coisa obsoleta que deve ser deitada fora. O hospital deve ter um quintal limpo e bonito.*

***RP-PM2:*** *Os enfermeiros as vezes vêm muito estressados e tratam mal os doentes. Serventes e enfermeiros devem ter carinho com os doentes. Eles despacham, isso não está bonito.*

1. **Acha que fazer o diagnóstico e tratamento imediato da tuberculose melhoraria o estado de saúde do paciente? *(Sondar: como? Ou de que maneira?*)**

***RP-PH6:*** *Muito. Tudo que se faz pontualmente dá resultados bons. Muitos atrasamos procurar o diagnóstico porque corremos atrás das farmácias sem antes ir a consulta. Ir a US o mais rápido possível é muito bom e ajuda a trazer sucessos.*

***RP-PH3:*** *Ajuda. Se você leva muito tempo sem diagnóstico, a situação fica péssima. Se você ficar com 35Kg ou 40 é uma derrota.*

***RP-PM2:*** *É, se você descobre cedo, começa a tratar cedo e melhora.*

***RP-PM5:*** *É bom porque melhora rápido*

- 1. **Acha que fazer o teste de HIV e iniciar o TARV melhoraria o estado da vida do paciente? Explique?**

***RP-PH3:*** *Melhora, porque quanto mais cedo tomar medicação, melhor.*

***RP-PH6:*** *As recomendações médicas são boas e devem ser cumpridas. Ajudam a minimizar as doenças oportunistas.*

***RP-PM1:*** *É muito bom fazer o tratamento enquanto é cedo, ajuda a melhorar.*

1. **Tem mais alguma coisa a acrescentar sobre o que já discutimos?**

***RP-PH6:*** *Elogiar a equipa, é importante que façam esses estudos, espero que os resultados contribuam positivamente. O que falha neste país é o não cumprimento de tanta coisa bonita que esta escrita. Era bom também que esses estudos sejam feitos em outras áreas. Nós queremos ver os efeitos desses estudos.*

***MUITO OBRIGADO (A) Hora do fim da entrevista___10H:06___***
