# Supplementary material for: Barriers to access and adherence to tuberculosis services, as perceived by patients: A qualitative study in Mozambique
Source: PLoS One. 2019 Jul 10;14(7):e0219470. doi: 10.1371/journal.pone.0219470 (PMC6619801; doi:10.1371/journal.pone.0219470)
Supplement: S1 Dataset — (ZIP) [file pone.0219470.s003.zip › Transcripts TB study/DGF3_.docx]

**A "Avaliação da Cascata de Cuidados de Pacientes Diagnosticados com TB, MDR-TB e Paciente Co-infectados com TB/HIV nas Províncias de Manica e Sofalaʺ**

# Instrumento: Guião De Entrevista para Grupos Focais - DGFs

**Data:** 12.02.2016

**Distrito:** Beira

**Nome da Unidade Sanitária**: CS Ponta-Gêa

**Hora do início:** 08H:40

**Hora do fim:** 09H:43

**Número de DGF:** 03

**Legenda**

**Anotador/a:** Alfredo Francisco Covele

**Entrevistador/a:** Maria Felicidade

**E:** Pergunta do(a) Entrevistador(a)

**P:** Participante/entrevistado(a)

**RP:** Resposta do(a) Participante/entrevistado(a)

**PH:** Participante Homem (seguido de sua posição de assento)

**PM:** Participante Mulher (seguida de sua posição de assento)

**n/a :** Não Aplicável

| Comentários/Observações Preliminares: *(circunstâncias que poderão influenciar a entrevista, etc.)* *A DGF correu bem. Tinha dois participantes, todos do sexo feminino. ADGF foi feita por de baixo de uma linda sombra, de uma grande e maravilhosa mangueira.* |
| --- |

**SECÇÃO A: ASSISTÊNCIA DO SERVIÇO DE SAÚDE AOS PACIENTES COM TB, MR-TB E TB-HIV**

1. **O que você sabe sobre TB?**

***RP-PM1:*** *TB é uma doença transmissível, talvez pelo ar, por exemplo quando alguém tosse sem tapar a boca. Numa casa com um doente de TB, o doente deve ficar distante de crianças, a casa deve ser arejada, e as janelas devem estar abertas para passar ar.*

***RP-PM2:*** *Também pode se transmitir comendo com alguém no mesmo prato e bebendo no mesmo copo.*

***RP-PM1:*** *Nós usamos chapa, se o doente estiver no chapa deve tapar a boca ao tossir para não transmitir a doença aos outros*

1. **O que você sabe sobre TB- MR?**

***RP-PM1:*** *A TB-MR é mais complicada. Os vírus são mais resistentes. Devemos fazer um tratamento mais forte para combater o vírus. Se não cumprirmos com o tratamento o vírus pode nos matar.*

***RP-PM2:*** *A TB-MR é muito complicada. Eu fiz um ano sem descobrir a doença. Tomei vários remédios, mas sempre vomitava, até que fui ao HCB e descobriram que era TB-MR. A TB-MR trata-se com injeções por dois anos. Devemo-nos alimentar corretamente, não se juntar com muitos homens de qualquer maneira, não consumir álcool, não perder noites, e não fumar.*

1. **O que acha sobre os serviços prestados neste sector de TB?**

***RP-PM1:*** *Acho que os serviços estão bem. Nos apoiam a continuar com o tratamento, se largarmos o tratamento nós é que ficamos prejudicadas. Vocês nos ajudam, se não nos ajudassem nós não nos sentiríamos confortáveis.*

***E: E o que acha dos serviços prestados?***

***RP-PM1:*** *Os serviços são bons, o tratamento dura dois anos, temos de vir e ir, estamos a melhorar.*

***RP-PM2:*** *Não é fácil andar todos os dias para o hospital, nós temos de ser bem atendidas.*

***RP-PM1:*** *Eu acho que está bem, mas deve melhorar o cumprimento do horário, nós devemos vir cedo e sem comer. Quando tomamos os comprimidos devemos ficar 30 minutos sem comer, daí é que podemos nos alimentar. De um modo geral devem melhorar o cumprimento do horário de atendimento.*

***RP-PM2:*** *Acho que os serviços estão bem. Como disse a minha colega devem melhorar o cumprimento do horário. Há doentes muito cansados, não têm dinheiro para alugar tchopela. Os comprimidos provocam dor, cansaço e areiam os joelhos.*

1. **Algum dia teve qualquer dificuldade durante o processo para acesso aos serviços de TB, TB-MR? Explique.**

***RP-PM1:*** *Eu tive. Quando comecei a ficar doente fiz muitos exames e tudo dava negativo. Chegou uma fase que ficava só em casa, não conseguia andar e tomava vários remédios que não tinham nada a ver. Depois de muito tempo minha mãe procurou um médico particular que descobriu a TB. Fiz o tratamento de seis meses, terminei e o resultado vou a dar positivo. Voltei a tratar, após terminar, o resultado novamente deu positivo. Agora estou a fazer injeções, disseram que é TB-MR. Já estou me sentindo bem e estou esperando o resultado do meu primeiro exame de controlo.*

***RP-PM2:*** *Tive dificuldades, andei em vários hospitais, fiz muitos exames e não acusava nada. Uma enfermeira disse que ia me ajudar, mas pediu 2000 MT, minha irmã não tinha o dinheiro. Fomos a Macuti, fiz o Raio X e descobriram TB. Fiz o tratamento de seis meses, mas minha barriga ficava sempre inchada e não melhorava. Voltei a ficar de baixa por mais duas vezes, daí descobriram que a TB era MR. Agora estou a tratar há dois meses e já estou me sentindo melhor. Eu nem conseguia falar.*

1. **O que sabe sobre HIV?**

***RP-PM1:*** *HIV é uma doença transmissível através de relações sexuais, através de objetos cortantes como agulha e lâminas. Se alguém se cortar com um objeto contaminado pode se infetar. HIV tem tratamento, e é até a morte. Se abandonar pode levar a morte.*

***RP-PM2:*** *HIV é uma doença que se transmite por via sexual e por objetos perfuro cortantes. Tem tratamento que não termina, deve ser feito até a morte.*

1. **O que foi mais dificil em compreender sobre TB e TB-MR?**

***RP-PM1:*** *Não foi dificil. Na minha familia tinha alguém com TB. Eu já sabia como a doença ia agir. Foi dificil no quisito ambiente, temos de nos isolar, os amigos nos abandonam, o namorado some, e muito desconfortavel para nos porque temos consciencia que podemos passar a doença para outras pessoas.*

***RP-PM2:*** *Eu tive um sobrinho que tinha TB, e era sempre eu que o levava ao hospital. O menino chegou a perder a vida, acho que apanhei dele.*

***RP-PM2:*** *Sim foi fácil.*

1. **Como é que pode ser feito o aconselhamento para ajudar um paciente a seguir com o tratamento de TB?**

***RPPM1:*** *Eu acho que devem apoiar o doente. Procurar saber como está, como vai o doente. Se o profissional se aproxima sempre do doente, nós ficamos sempre motivados a continuar com o tratamento. O tratamento é muito longo.*

***RP-PM2:*** *Apoiar, aconselhar.*

***E:*** *Aconselhar o que?*

***RP-PM2:*** *Dizer que não podem abandonar, aconselhar a se alimentar bem. Procurar os pacientes quando sentirem a sua ausência, uma vez que tem o nosso endereço, assim como fazem com os doentes de HIV.*

**SECÇÃO C: ADESÃO AOS SERVIÇOS TB**

***(Geralmente é difícil para muitos pacientes aderirem ao tratamento TB,TB-MR e TB/ HIV).***

1. **Quais são os problemas que os doentes enfrentam para iniciar o tratamento com:**
2. **TB?**

***RP-PM1:*** *Para iniciar o tratamento não tem tido muito problema. O problema começa quando iniciamos o tratamento. O tratamento leva a problemas de pés e joelhos. Os que têm tensão, o tratamento pode piorar a tensão. Nos primeiros dias das injeções provoca muita febre.*

***RP-PM2:*** *Não foi nada fácil. Estava muito doente, não conseguia andar.*

***RP-PM2:*** *Eu não tive problemas. A primeira consulta que fiz, eu expliquei tudo e o enfermeiro sugeriu que eu fizesse análises de TB, mas deu negativo. Minha mãe teve que pagar 1000MT no LAC para diagnosticar a doença. Logo que descobriram comecei com o tratamento.*

***RP-PM2:*** *Eu fui cobrada 2000MT, mas não tinha, e tive que ir ao HCB, e lá não me cobraram nada.*

1. **TB-MR?**

***RP-PM1:*** *Eu não tive muitos problemas, porque já vinha fazendo outros tratamentos de TB. Foi apenas mudar o tipo de tratamento.*

***RP-PM2:*** *Eu tinha problema de inchaço de barriga, e achava que era feitiçaria, fui em vários curandeiros e como acabava de terminar um tratamento de TB, foi difícil acreditar que ainda tinha TB.*

1. **TB- HIV?**

*n/a*

1. **Quais são os aspetos que foram mais difíceis para continuar a fazer o tratamento?**

***RP-PM1:*** *O mais difícil é a alimentação, porque há pessoas que não têm condições. É preciso tomar leite fresco, não comer piripíri, e não consumir muito óleo. Tem gente que não tem condições, tomam remédios e não se alimentam, o que pode areá-los. Não basta só tomar o remédio e não se alimentar.*

***RP-PM2:*** *Os remédios de TB e HIV são muito fortes, se você não comer bem os remédios podem te arear.*

***E: Para além de alimentação não tem outra coisa?***

***RP-PM1:*** *O principal é a alimentação, se não se alimentar bem os comprimidos podem prejudicar-te***.**

**SECÇÃO D: MELHORAR O LABORATÓRIO E PNCT**

1. **Existe algo que poderia ser melhorado nos serviços de PNCT?**

***RP-PM1:*** *Penso que não têm muito a melhorar. Acho que devia haver uma forma de se reduzir o número de comprimidos. Eu por exemplo tomo 12 comprimidos de manha e quatro atarde, isso depende do peso.*

***E:*** *E quanto ao laboratório?*

***RP-PM1:*** *O laboratório as vezes diz que a análise está contaminada e que devemos repetir, em suma deve se melhorar a parte de segurança no laboratório.*

***E:*** *E aqui na Ponta-Gêa?*

***RP-PM1:*** *Aqui também deve-se melhorar a parte de segurança. Aqui sentamos todos juntos, enquanto a recomendação é sentarmos separados, os de TB-MR num sítio e os outros noutro sítio.*

***RP-PM2:*** *Para mim quase tudo está bem. Não vejo muita coisa errada, as vezes atendem mal, as vezes bem, depende da temperatura da pessoa nesse dia.*

- 1. **O que deve ser feito pela US na seleção ao tratamento e sua continuidade?**

***RP-PM1:*** *Eu acho que não há muito a melhorar. Eu acho que só o atendimento deve melhorar. Um doente é como uma criança, o profissional deve atender o doente, ao invés de insultá-lo.*

***RP-PM2:*** *Nós doentes as vezes ficamos nervosos e estressados, é preciso ter paciência. Eu por exemplo facilmente fico zangada, e quando é assim começo a sofrer de peito.*

- 1. **O que o trabalhador de saúde poderia fazer para melhorar aderência ao tratamento?**

***RP-PM1:*** *Dando mais força, apoiando o doente, tratando o doente de forma natural, sem nos descriminar.*

***RP-PM2:*** *Os trabalhadores devem se comportar bem.*

***E: Como deve ser o atendimento?***

***RP-PM2:*** *Deve haver justiça, o que vem primeiro deve ser atendido primeiro. Há vezes que são atendidos os vêm tarde, ao invés dos primeiros. Isso não fica bem.*

1. **Acha que fazer o diagnóstico e tratamento imediato da tuberculose melhoraria o estado de saúde do paciente? *(Sondar: como? Ou de que maneira?*)**

***RP-PM1:*** *Sim. Por exemplo se o doente fizer análises completas, isso evitaria o doente fazer tratamentos errados antes de se descobrir a verdadeira doença. Por exemplo o laboratório da Ponta-Gêa não diz que tipo de TB se trata, apenas diz que a pessoa tem TB.*

***RP-PM2:*** *Sim, porque antes de a doença abranger o corpo era bom que o diagnóstico fosse rápido e específico.*

- 1. **Acha que fazer o teste de HIV e iniciar o TARV melhoraria o estado da vida do paciente? Explique?**

***RP-PM1:*** *Sim. Porque há pessoas que estão co infetadas. É importante fazer os dois tratamentos, porque se fizer apenas um, a outra doença pode causá-lo problemas. Se a pessoa fizer um tratamento de cada vez vai demorar a melhorar.*

***RP-PM2:*** *Sim. Porque combate as doenças do teu corpo. É sempre bom fazer os dois tratamentos, caso a pessoa seja co infetada.*

1. **Tem mais alguma coisa a acrescentar sobre o que já discutimos?**

***RP-PM1:*** *Acho que não temos mais nada para acrescentar.*

***RP-PM2:*** *Acho que falamos tudo o suficiente.*

**MUITO OBRIGADO (A) Hora do fim da entrevista___09H:43__**
