# Supplementary material for: Barriers to access and adherence to tuberculosis services, as perceived by patients: A qualitative study in Mozambique
Source: PLoS One. 2019 Jul 10;14(7):e0219470. doi: 10.1371/journal.pone.0219470 (PMC6619801; doi:10.1371/journal.pone.0219470)
Supplement: S1 Dataset — (ZIP) [file pone.0219470.s003.zip › Transcripts TB study/DGF4_.docx]

**"Avaliação da Cascata de Cuidados de Pacientes Diagnosticados com TB, MDR-TB e Paciente Co-infectados com TB/HIV nas Províncias de Manica e Sofalaʺ**

# Instrumento: Guião De Entrevista para Grupos Focais - DGFs

**Data:** 16.02.2016

**Distrito:** Nhamatanda

**Nome da Unidade Sanitária**: HRN

**Hora do início:**10H:06

**Hora do fim:** 12H:26

**Número de DGF:** 04

**Legenda**

**E:** Pergunta do(a) Entrevistador(a)

**P:** Participante/entrevistado(a)

**RP:** Resposta do(a) Participante/entrevistado(a)

**PH:** Participante Homem (seguido de sua posição de assento)

**PM:** Participante Mulher (seguida de sua posição de assento)

**n/a :** Não Aplicável

| Comentários/Observações Preliminares: *(circunstâncias que poderão influenciar a entrevista, etc.)* *A DGF correu bem. Foi feita em um local aberto, na varanda da enfermaria de TB. Tivemos quatro participantes, dois do sexo masculino e dois do sexo feminino.* |
| --- |

**SECÇÃO A: ASSISTÊNCIA DO SERVIÇO DE SAÚDE AOS PACIENTES COM TB, MR-TB E TB-HIV**

1. **O que você sabe sobre TB?**

***RP-PM3:*** *TB é uma doença transmitida através da tosse. Por exemplo piringanhisso é TB. Os curandeiros dizem que TB é piringanhisso. O hospital diz que essa doença é TB.*

***RP-PM4:*** *TB é provocada por piringanisso. As vezes o homem anda com outras mulheres lá fora, adoece, vai ao curandeiro, quando não cura é quando vai ao hospital fazer o tratamento.*

***RP-PH2:*** *Eu vi quando trabalhei com pastor, sempre voltava para casa. Três semanas depois comecei a tossir de longe, mas depois vim para aqui fazer o tratamento mas não melhorei, é quando me fizeram o teste de HIV e comecei a melhorar, mas temos problemas de medicamento, porque sonho muito mal. Muita das vezes o medicamento provoca muita reação adversa. As vezes o medicamento pede comer muito e não temos comida.*

***RPPM4:*** *As vezes falam estão a desistir, mas é por causa da reação dos medicamentos. Tomar comprimidos sem comer nada é muito difícil. Só beber água é muito difícil.*

***RP-PM3:*** *Este medicamento provoca muita fome e não temos o que comer.*

***RP-PH2:*** *Só nós somos muitos, mas o estado não aguenta dar-nos comida. Muitos amigos meus deitam os comprimidos.*

***RP-PH1:*** *TB é uma doença provocada por fumar e por piringanhisso, por exemplo eu ando na estrada descontrolado, vou ao curandeiro, e depois vou ao hospital. Quando vim ao hospital era muito tarde e disseram que era TB. Mais tarde fizeram o teste de HIV e me disseram que tenho SIDA. Os comprimidos dão nos bem, não tem problema. Eu não como massa nem arroz, só papa.*

1. **O que você sabe sobre TB- MR?**

***RP-PM4:*** *Nós somos crianças, e não sei, há muito tempo tinha tratamento, mas agora não se trata e não sabemos porquê.*

***RP-PM3:*** *Essa TB com esse tratamento a pessoa cura, basta cumprir com o tratamento. Eu cumpri e estou curada, este ano fui a machamba, mas Deus não faz cair chuva.*

***RP-PH1:*** *Eu sou criança, não conheço esse tratamento.*

***RP-PM3:*** *De onde vem essa TB não sei, mas muitas vezes é por causa de aborto e nado mortos. Há crianças também fazendo o tratamento, mas não sabemos de onde vem o tratamento. Alguns dizem que e um bicho. Outros desde que nasceram nunca andaram fora, mas têm essa doença.*

1. **O que acha sobre os serviços prestados neste sector de TB?**

***RP-PH2:*** *Eu fui bem tratado. Vim aqui sem andar, sem reconhecer as pessoas. Comecei a fazer o tratamento durante uma semana, depois me disseram que tinha TB, e daí comecei o tratamento durante seis meses. Naquele tempo eles davam matabicho, mas agora já não, e não sei em qual das portas estão a tratar bem.*

***RP-PM3:*** *Em caso de tratamento, está muito bem, só é difícil fazer o seguimento, mas alí no sector tratam bem.*

***RP-PM4:*** *O tratamento no sector de TB está andar bem. Você que é doente avalia como é a receção. Quando eu vim fui bem recebida e ainda estão a me tratar bem. Na hora da comida me chamam para levar a comida. Os meus pés estavam inchados, mas agora estou a melhorar, já ando até aí nos bancos.*

***RP-PH1:*** *O tratamento está andar bem. Estou aqui a três meses e está tudo a andar bem, mas agora só como papa.*

1. **Algum dia teve qualquer dificuldade durante o processo para acesso aos serviços de TB, TB-MR? Explique.**

***RP-PH1:*** *Eu não tive problemas.*

***RP-PM4:*** *Eu sempre fui vindo várias vezes, mas nunca descobriram a doença. Sempre me deram vários comprimidos, e fui guardando as receitas. Mais tarde quando estive mal, trouxe todas as receitas, é quando me fizeram a análise de TB, e me disseram para baixar e baixei.*

***RP-PM3:*** *Fiquei no hospital, fui dada comprimidos várias vezes, mas não descobriram a minha doença. Mais tarde foi quando conseguiram fazer-me a análise de TB, que deu positivo e comecei a fazer o tratamento.*

***RP-PH2:*** *A minha dificuldade é do tratamento de TB e HIV, porque este tratamento é muito forte. As vezes penso que me feitiçaram por causa da reação dos medicamentos. Há vezes que para tomar, a pessoa pensa várias vezes, mas acabo tomando com medo da recaída.*

1. **O que sabe sobre HIV?**

***RP-PM4:*** *Por minha parte eu estou atrapalhada querendo saber que tipo de doença é. Você não tem família, tem de tomar os medicamentos e não consegue ficar de pé, está toda hora a cair e ficar grossa. Que tipo de doença é esta?*

***RP-PH1:*** *SIDA é uma doença de relações sexuais.*

***RP-PM3:*** *Dizem que se provoca com lâmina, injeções e relações sexuais. Muitas pessoas dizem que é um bicho, mas como aparece não sei. Eu por exemplo já estou na menopausa e não ando com homem, mas já apanhei esta doença.*

***RP-PH2:*** *Esta doença nos procura. As vezes nós falamos que é uma doença de brancos estrangeiros, as vezes que é uma doença causada por objetos cortantes. Mesmo entre casais em casa, cada um tem medo do seu parceiro, quando sai e volta tarde, nós desconfiamos. Por vezes dão livros para lembrar, mas está difícil compreender. Há vezes que esta doença afeta crianças, esta doença jamais vai acabar. Quando você fica uma semana sem tomar os remédios, ela há-de vir com muita força.*

1. **O que foi mais dificil em compreender sobre TB e TB-MR?**

***RP-PM4:*** *Respondam vocês os mais velhos porque nós nascemos agora e não conhecemos esta doença. Nós encontramos vocês.*

***RP-PM3:*** *Eu tambem não sei que tipo de TB é essa que não tem tratamento.*

***RP-PH2:*** *As vezes esses nossos filhos vão a escola, engravidam e fazem aborto, chegam em casa cozinham para a familia e infelizmente isso faz mal, provoca TB-MR. Quando a pessoa trabalha longe e em casa acontece falecimento, quando a pessoa chega deve pegar medicamento, se não tratar vai ficar doente.*

***RP-PH1:*** *As vezes tem sido a queimadura de casa. Quando não se tratar também é uma doença TB-MR. Muitas vezes só descobrimos no hospital.*

***RP-PH2:*** *Eu fiz bem vir no hospital, se não já teria morrido.*

***RP-PM3:*** *Eu comecei a sentir barrulho na barriga, pouco tempo depois vomitei sangue e fiz diarreia com sangue também. Cheguei aqui no hospital, me receberam e me deram guia para o HCB, mas não tinha dinheiro.*

1. **Como é que pode ser feito o aconselhamento para ajudar um paciente a seguir com o tratamento de TB?**

***RP-PM3:*** *Outros desistem por falta de fé com o tratamento. É preciso cumprir, outros fazem deproposito porque a cura da doença é muito lenta.*

***RP-PH2:*** *Os enfermeiros nos aconselham bem, mas muitas vezes é por nosso dislexo, tipo eu já morri. Compara-se com as pessoas que vão a igreja, outras dizem vocês que vão a igreja, estão a fazer o quê quando vir a salvação. Os enfermeiros andam nas nossas casas, e por vezes até levam medicamentos para nós, só a doença é que é difícil.*

***RP-PM3:*** *É preciso perseguir, mas alguns aceitam regressar, outros até morrem em casa com medo do hospital. Outros fogem comprimidos. Duas meninas fugiram comprimidos aqui no hospital, elas saíram com o seu pai. Elas podem fugir, mas a doença é tua, não te larga.*

***RP-PM4:*** *Eu com meus dois filhos já vim no hospital, mas minhas amigas sempre me questionavam, porquê estas a levar a criança no hospital, ela não vai viver. Eu respondi, eles podem matar a minha criança, não há problema.*

**SECÇÃO C: ADESÃO AOS SERVIÇOS TB**

***(Geralmente é difícil para muitos pacientes aderirem ao tratamento TB,TB-MR e TB/ HIV).***

1. **Quais são os problemas que os doentes enfrentam para iniciar o tratamento com:**
2. **TB?**

***RP-PH2:*** *O que e difícil é não saber qual é a sua doença. Muitas vezes dão outros medicamentos que não são desta doença. Muitas vezes quando vamos ao laboratório nos dão frascos para fazer análise, quando sai positivo é quando tomamos medicamentos.*

***RP-PM4:*** *Outras pessoas é medo de baixar, preferem morrer, outros dão prioridade aos serviços de casa. Eu estou sozinha aqui no hospital, não tenho família, se for para morrer vou morrer, depende de Deus. Quando alguém cozinhar sua papa, eu vou pedir, quando chega a hora de comida, eu costumo ir levantar. A minha mãe está a tossir, mas ela não aceita vir ao hospital.*

***RP-PM3:*** *Medo de baixar, tomar medicamento ou morrer no hospital.*

***RP-PH1:*** *Muitos têm medo de injeção, por vezes pensam que o enfermeiro põe medicamento para matar na garrafa se soro.*

1. **TB-MR?**

*n/a*

1. **TB- HIV?**

***RP-PH1:*** *As pessoas querem tomar, mas o que é difícil é fome. Os comprimidos de TB são grandes e fortes, e os comprimidos de HIV provocam muita alucinação. Sonhar com cemitério, a lutar, com caixão, e quando você acorda está bem partido. Estes medicamentos exigem matabichar soja. Se você não comer nada desde manha vai morrer.*

***RP-PH2:*** *Nós que tomamos medicamentos, precisamos de receber medicamentos. Quando estava cá a PEPFAR eu recebia papa, mas agora como não tenho família aqui nunca me deram papa. Por vezes dão-se soja entre amigos. É muito difícil, não temos comida, só comemos pão. Quando você aproxima o enfermeiro, fazem passa-passa como bola e no fim do dia a pessoa volta sem nada para casa.*

***RP-PM4:*** *Como eu assim não tenho ninguém, vou fazer o quê, vou morrer.*

***RP-PH2:*** *Muitas vezes abandonos aumentam por falta de comida.*

***RP-PM3:*** *Como nós aqui, não nos dão nada.*

***RP-PH2:*** *Como vocês são da direção e estão a procurar pessoas para conversar, mas está difícil.*

***RP-PM3:*** *Eu só recebi uma vez papa de soja, mas as outras pessoas recebem sempre.*

1. **Quais são os aspetos que foram mais difíceis para continuar a fazer o tratamento?**

***RP-PM4:*** *Não sabemos porque cada pessoa está em casa dele. É difícil saber, eu não vou abandonar, sempre venho aqui no hospital.*

***RP-PM3:*** *Outros negam por não entenderem a sua importância.*

***RP-PH1:*** *Outros negam porque gostam de seguir ambiente, gostam de beber. Quando pensam na bebida, pensam que estão a perder muito.*

***RP-PM4:*** *Saúde e doença o que é mais bom? Eu aqui posso ficar quatro anos sem ir a machamba, o que me importa é ter uma boa saúde.*

***RP-PH2:*** *Abandonar o medicamento vai prejudicar-se a si próprio.*

***RP-PH1:*** *Outros levantam medicamentos e deitam. Tem ainda outros que vão fumar cigarro na casa de banho.*

***RP-PM4:*** *Aqui proíbem cigarro, tabaco, piripíri, mas outros não cumprem, sempre desobedecem.*

***RP-PM3:*** *A pessoa que adoece não pode beber, fumar, comer piripíri, nem manter relações sexuais, mas por causa da teimosia nós desobedecemos.*

***RP-PH2:*** *Esse comportamento não fica bem, fica perdido.*

***RP-PM4:*** *Se eu melhorar vou pedir emprego em casa de quem quer que seja.*

***RP-PH1:*** *Pessoas levam remédios para deitar.*

**SECÇÃO D: MELHORAR O LABORATÓRIO E PNCT**

1. **Existe algo que poderia ser melhorado nos serviços de PNCT?**

***RP-PM4:*** *Gostaria que nos apoiassem com alimentação, sobretudo soja para combater a fome.*

***RP-PH1:*** *Gostaria que nos dessem soja.*

***RP-PM3:*** *O estado deve nos apoiar com alguma alimentação, para nós aguentarmos com o tratamento.*

***RP-PH2:*** *A direção deve nos ajudar com comida, para nós podermos aguentar com a medicação. Sintam pena de nós por favor.*

- 1. **O que deve ser feito pela US na seleção ao tratamento e sua continuidade?**

**RP-PM6: *RP-PM6:*** *Nós como estámos a iniciar, estámos a aprender apenas.*

***RP-PH2:*** *Gostaria que reduzissem as gramas dos medicamentos porque são muito fortes e nós não aguentamos. Quando falamos das reações dos medicamentos não nos é dada uma resposta certa. As vezes nos respondem que quando a direção vier vamos chamar-te para falar com eles. Também há muita troca de medicamentos, gostaria que o tratamento fosse o mesmo do princípio ao fim porque os comprimidos são grandes, outros cortam e tomam metade. Os doentes não vêm depois de 30 dias, vêm depois de 60 dias, porque tem stock de medicamento cortado.*

***RP-PH1:*** *Gostaria que melhorassem a higiene, quem varre no hospital somos nós doentes.*

***RP-PM4:*** *Não temos água aqui no hospital.*

***RP-PH1:*** *Tem muita água, mas os que guardam a chave é que dificultam. Os medicamentos que estão a me dar não estão bons. Eu estou a tomar 12 comprimidos, quando falo que não me sinto bem, eles dizem que o tratamento é esse mesmo. Dantes diziam que iam trocar para o tratamento injetável, mas até agora ainda não trocaram.*

***RP-PH2:*** *Por vezes tem sido difícil todos os meses fazer análises e não nos dão o resultado. Só todos os meses tiramos sangue e não nos dizem qual é o resultado. Gostaria que nos dissessem o resultado.*

***RP-PM3:*** *Vocês são novos não e? Eu faço análise uma vez por ano, fiz em janeiro.*

- 1. **O que o trabalhador de saúde poderia fazer para melhorar aderência ao tratamento?**

***RP-PM1:*** *Gostaria que nas segundas-feiras o enfermeiro de TB fizesse uma palestra a convidar todos os doentes com tosse para fazer análise, porque muitos chegam e não são bem dirigidos para o setor de TB. Gostaria também que os trabalhadores do setor de TB fossem a procura dos doentes com tosse nos centros de saúde periféricos para serem tratados.*

***RP-PH2:*** *O enfermeiro deve preocupar-se com o doente, não deve só preocupar-se com a administração do medicamento, deve também perguntar como está, onde está, onde dói e mais.*

***RP-PM4:*** *Gostaria que os quartos tivessem redes mosquiteiras, mas outros lados tem rede mosquiteira.*

***RP-PM3:*** *Deve ter um servente para a enfermaria. Os enfermeiros não têm problemas, nós sozinhos é que temos problemas.*

***RP-PH2:*** *Tratar os doentes com carinho.*

1. **Acha que fazer o diagnóstico e tratamento imediato da tuberculose melhoraria o estado de saúde do paciente? *(Sondar: como? Ou de que maneira?*)**

***RP-PH1:*** *É bom iniciar o tratamento porque não desgasta o paciente.*

***RP-PM4:*** *É bom porque a sua saúde melhora, mas primeiro vai ao laboratório, depois vai fazer análises, depois do resultado mandam para o setor de TB para iniciar o tratamento. Por exemplo mesmo indo ao curandeiro ele não dá logo tratamento, primeiro faz a consulta depois dá o tratamento.*

***RP-PH2:*** *Depende de cada doente. Alguns já vêm com guia de transferência, quando chegam iniciam logo o tratamento.*

***RP-H1:*** *Eu primeiro vim para cá, fiz o Raio X, depois fui ao encontro do médico, ele assinou o meu processo e me enviou para o setor de PNCT.*

***RP-PH2:*** *Sim é bom porque melhora a saúde, mas tem de fazer análise, pesar para ver que quantidade de medicamento vai tomar, para não tomar nem mais, nem menos.*

- 1. **Acha que fazer o teste de HIV e iniciar o TARV melhoraria o estado da vida do paciente? Explique?**

***RP-PH3:*** *Iniciar o tratamento imediatamente é bom porque evita desgastar o organismo. É preciso atacar logo com o tratamento.*

***RP-PH2:*** *Sim melhoraria porque ajuda a saúde do doente. Primeiro começa-se a fazer o tratamento aqui no hospital. Depois de uma semana é quando dão comprimidos correspondentes a uma semana. Este processo de busca é bom porque ajuda àqueles carentes. Antigamente nós recebíamos dinheiro de passagem para o regresso, isso nos ajudava muito, mas agora já não dão. É bom fazer o teste. Depois tens de voltar para iniciar o tratamento. Eu fiquei um mês a tomar outros comprimidos. Depois fui aconselhado, é quando comecei este tratamento. Muitos desistiram alegando que é o próprio comprimido que mata.*

***RP-PM3:*** *Primeiro fiz teste. Depois do resultado fiz o Raio X, após o Raio X comecei com tratamento duas semanas depois.*

***RP-PH2:*** *Sim é bom, mas depende do organismo de cada um. Temos de fazer análise e ter aconselhamento para não desistirmos. Eu tomei os medicamentos aqui mesmo no hospital porque estava de baixa. Eu sempre levanto aqui os medicamentos.*

1. **Tem mais alguma coisa a acrescentar sobre o que já discutimos?**

***RP-PH2:*** *Não temos mais nada a dizer.*

***RP-PH1:*** *Apenas agradecer por nos terem ouvido.*

***RP-PH3:*** *Não tenho nada a dizer.*

***RP-PM4:*** *Não tenho nada, tudo já foi falado***.**

**MUITO OBRIGADO (A) Hora do fim da entrevista___12H:26___**
