# Supplementary material for: Barriers to access and adherence to tuberculosis services, as perceived by patients: A qualitative study in Mozambique
Source: PLoS One. 2019 Jul 10;14(7):e0219470. doi: 10.1371/journal.pone.0219470 (PMC6619801; doi:10.1371/journal.pone.0219470)
Supplement: S1 Dataset — (ZIP) [file pone.0219470.s003.zip › Transcripts TB study/DGF5_.docx]

**"Avaliação da Cascata de Cuidados de Pacientes Diagnosticados com TB, MDR-TB e Paciente Co-infectados com TB/HIV nas Províncias de Manica e Sofalaʺ**

# Instrumento: Guião De Entrevista para Grupos Focais - DGFs

**Data:** 17. 02. 2016

**Distrito:** Nhamatanda

**Nome da Unidade Sanitária**: HRN

**Hora do início:** 09H:23

**Hora do fim:** 12H:45

**Número de DGF:** 05

**Legenda**

**E:** Pergunta do(a) Entrevistador(a)

**P:** Participante/entrevistado(a)

**RP:** Resposta do(a) Participante/entrevistado(a)

**PH:** Participante Homem (seguido de sua posição de assento)

**PM:** Participante Mulher (seguida de sua posição de assento)

**n/a :** Não Aplicável

| Comentários/Observações Preliminares: *(circunstâncias que poderão influenciar a entrevista, etc.)* *A DGF correu bem. Foi feita na varanda da enfermaria de TB, um local aberto, e participaram seis pessoas, cinco do sexo feminino e um do sexo masculino. No final foi oferecido um lanche aos participantes.* |
| --- |

**SECÇÃO A: ASSISTÊNCIA DO SERVIÇO DE SAÚDE AOS PACIENTES COM TB, MR-TB E TB-HIV**

1. **O que você sabe sobre TB?**

***RP-PM1:*** *Tuberculose é doença dos pulmões que quando os bichos começam a comer, você começa a tossir, não consegue comer, fica sem vontade e fica muito magrinha. Só quando você vai para o hospital é quando descobrem a doença. Você começa a tomar comprimidos e fica melhor. Depois de iniciar o tratamento ficas gorda, começas a comer muito, mas muito mesmo. Comecei a apanhar esta doença há vinte anos atrás, trataram-me, fiquei bem, fiz três filhos, e uma das minhas filhas tem 35 anos. Em 2014 comecei com o tratamento, mas não fiquei melhor. Fui para Maputo e continuei a fazer o tratamento, mas mesmo assim não melhorei. Fiz o tratamento por seis meses, voltei a Nhamatanda, continuei com as análises, aí o médico descobriu a minha doença e disse-me que ia melhorar e prescreveu injeções durante 60 dias e 12 comprimidos. Até agora já apanhei 10 injeções. Aqui no hospital é minha casa, não vou desistir.*

***RP-PM2:*** *Comecei com minha doença quando tinha 17 anos, fiz tratamento e curei. Fiz meus três filhos e agora recaí. Agora estou a fazer tratamento de injeções. Sobre a TB eu não sei o que é e nem sei como apanhei essa doença. Talvez apanhei pelas minhas mãos ou pelos pés, mas não sei.*

***RP-PM6:*** *TB é tosse, mas vim cá fiz análise e disseram que tenho TB. Essa doença não sei como apanhei. Se eu fosse menina podia dizer que apanhei lá fora, mas nesta minha idade, nem marido não tenho, talvez apanhei pelas injeções ou lâminas, mas na verdade não sei como apanhei essa doença.*

***RP-PM5:*** *TB é uma tosse. Comecei a vomitar e vim cá fazer análise e descobriram que estou com TB, e já estou a fazer tratamento com comprimidos. Comecei com o tratamento em Janeiro, ainda não fiz dois meses. A pessoa sente dores de peito, emagrecimento e fica sem vontade para comer. Como apanhei a doença, eu não sei, talvez por agulhas ou lâminas.*

***RP-PM4:*** *Comecei em Agosto, muita gente dizia que minhas filhas andavam fora. Fui ao curandeiro, deram-me medicamentos e não curei. Fiz tratamento de piringanisso, também não curei. Vim cá no hospital, fiz análise e já estou melhor e tenho dois meses a fazer o tratamento.*

***RP-PH3:*** *Outros falam que TB é uma falha de casa. Fui no curandeiro trataram mas não curei. Vim para o hospital em Novembro de 2014, estou a fazer tratamento e estou melhor.*

*RP-PM2: Muitos dizem que é falha em casa. Mas em casa vemos crianças com essa doença, também é falha?*

1. **O que você sabe sobre TB- MR?**

***RP-PH3:*** *TB-MR é esta minha porque não pega medicamento, porque comecei há muito tempo, mas não estou a melhorar. Fui para Mopeia fazer o tratamento, mas não melhora e vomitei sangue. Não consigo andar longas distâncias, nem subir montanhas. Sempre que ando um pouco tenho problemas de respiração. Não sei de onde veio esta doença.*

***RP-PM1:*** *Esta TB que não tem cura será que um dia vai passar? Todo o momento estamos a cumprir o tratamento, mas não estamos a curar. Eu desde 2013 até hoje nunca desisti, mas não estou a curar. Estou sempre a chorar por causa da minha doença, e por vezes vomito sangue.*

***RP-PM6:*** *Esta TB veio por causa dos nossos antepassados que não seguiram com os mandamentos. Antigamente nós cumpríamos com a tradição. Agora as igrejas estragaram a tradição, e as nossas filhas também não cumprem e fazem muitos abortos.*

***RP-PM5:*** *Esta TB-MR é isso que falou essa mãe, é mesmo piringanisso, nós não fazemos mais cerimónia de cupitacufa. Só estamos a seguir as normas da igreja, e nem as normas da igreja estamos a seguir, quando vamos no curandeiro dizem que é piringanisso. Quando tratam não cura, esta doença é muito complicada.*

***RP-PM4:*** *TB-MR nas nossas casas está a acontecer porque nós não seguimos com as normas. Muitas vezes carregamos bidons de 20 litros, vamos a machamba, cozinhamos, e mesmo tratados a doença recomeça.*

1. **O que acha sobre os serviços prestados neste sector de TB?**

***RP-PM6:*** *Os serviços prestados no sector de TB estão a correr muito bem. Somos bem atendidos, os enfermeiros não gingam e estamos a ser tratados sem pagar nenhum valor monetário.*

***RP-PM2:*** *Os serviços estão sendo bem prestados neste sector. Atendem-nos como família deles, como se fosse mãe ou irmã deles. Quando não conhecemos um sector o enfermeiro nos acompanha. As pessoas muito magrinhas eles atendem.*

***RP-PM5:*** *Eles atendem bem, mesmo atrasados eles atendem e não insultam. Mesmo bem magrinho eles atendem e não te descriminam.*

***RP-PM1:*** *Gostaríamos que depois do tratamento os enfermeiros nos aconselhassem sobre o que devemos fazer. Por vezes estamos doentes, e quando você termina o tratamento pedem-nos para fazer análises de controlo ou Raio X, ou BK para ver sobre o seu estado depois do tratamento.*

***RP-PH3:*** *Para nós doentes de TB, neste sector estão a atender muito bem, mesmo chegando tarde eles nos atendem bem.*

***RP-PM6:*** *Noutros sectores te mandam voltar a casa, caso chegues tarde. Neste sector não, por exemplo hoje eu cheguei tarde, mas me deram medicamentos.*

1. **Algum dia teve qualquer dificuldade durante o processo para acesso aos serviços de TB, TB-MR? Explique.**

***RP-PM6:*** *Por mim não tive dificuldades, cheguei, receberam, deram-me papeis para análise, fiz as analises, logo tive o resultado e iniciei o tratamento.*

***RP-PM1:*** *Eu quando comecei fiquei um ano inteiro sem descobrir a minha doença, foi todo 2013. Depois acabei falando que a minha tosse é muito complicada, pedi ao médico que quero ir fazer Raio X, daí é que despertei o médico que na verdade tinha TB. Foi quando ele me prescreveu o tratamento de TB, mas até hoje não vejo melhoria.*

***RP-PM5:*** *Eu quando vim estava com febre e disseram que era malária, tomei comprimidos de malária, mas sempre estive a tossir, o peito me aquecia, não conseguia varrer, voltei pela segunda via, disse que ainda estou a tossir, deram-me frasco, fiz análise e descobriram a doença. Comecei a fazer o tratamento e já estou a melhorar. Estou a tossir, mas poucas vezes. Fui nos profetas, gastei dinheiro mas não melhorei.*

***RP-PM6:*** *O governo está a trabalhar muito para satisfazer a população, mas não está a conseguir, está difícil.*

***RP-PM1:*** *Esta doença para mim está muito complicada, não sei se vou curar.*

***RP-PH3:*** *Comecei com febre, mas sempre vim, mas deram-me medicamentos de malária e não curei. Mandaram-me fazer análise, foi quando descobriram a doença. Eu por causa da tradição e curandeiro não posso comer galinha, cabrito, por causa de sangue.*

***RP-PM6:*** *Quando ver que o problema está nos pulmões deve fazer Raio X.*

***RP-PH3:*** *Quando estiver doente, não se deve matar nenhum animal. Eu só me alimento de vegetais.*

1. **O que sabe sobre HIV?**

***RP-PM6:*** *SIDA é provocada por nossos filhos, fazem abortos, mantêm relações sexuais e provocam SIDA. Depois dizem que é bicho da SIDA. Há muito tempo diziam que é doença de estrangeiro, os estrangeiros dormiam com cães grandes e provocou SIDA.*

***RP-PM1:*** *HIV é uma doença que se contrai pelas relações sexuais com homem infectado, mas TB é uma doença provocada por não cumprir com a tradiçao.*

***RP-PH3:*** *HIV está nas relaçoes sexuais nos quiosques, estrada e muitas vezes os enfermeiros falam para usarmos preservativos.*

***RP-PM6:*** *Muitas vezes nós os mais velhos estamos a levar HIV nas lâminas e injeções, nos enfermeiros na zona.*

***RP-PM2:*** *HIV para os mais velhos apanham nos bares, nas bebidas, onde se encontram com as jovens. Elas as jovens, quando lhes pagam bebidas, estas por sua vez pagam com sexo.*

***RP-PM1:*** *Os velhos agora não sossegam, estão toda hora atrás das jovens e eles não te largam, logo violam para pagar a bebida que te pagou. É muito feio o que fazem os velhos.*

***RP-PH3:*** *Os jovens de hoje não aceitam perder, se eles gostaram do seu dinheiro, querem tirar proveito com os mais velhos porque eles querem distribuir a doença.*

***RP-PM2:*** *Outras podem andar com roupa curta, mas não gostam de andar com muitos homens de qualquer maneira, só depende de coração de cada um.*

***RP-PM1:*** *Tenho minha filha doente, mas não aceita fazer o tratamento. Ela só está a esperar o dia que Deus vai-lhe levar. Eu estou cansada com ela.*

1. **O que foi mais dificil em compreender sobre TB e TB-MR?**

***RP-PM1:*** *Foi dificil compreender porque não sei como apanhei esta doença. Não sei se veio a partir dos meus pais, mas gostaria de saber.*

***RP-PM2:*** *Eu não compreendo como apareceu esta doença, mas minha mãe me disse que eu tive asma na minha infância, não sei se é complicaçao da asma, espero que os enfermeiros me façam compreender.*

***RP-PM6:*** *Está difícil compreender de onde é que saiu esta doença, muitas vezes sei que é por causa de matar galinha num falecimento. Aconteceu com um homem, que quando chegou da viagem, a filha preparou uma galinha, antes dele comer vomitou sangue, é daí que começou sua doença.*

***RP-PM1:*** *Isso já era para acontecer.*

***RP-PH3:*** *Não minha mãe. Isso acontece quando sua filha abortar e cozinhar para si, ou pôr sal na comida, você fica doente na hora.*

***RP-PM6:*** *Nós estamos a pedir o governo para procurar medicamentos para nós. Nós queremos saúde.*

***RP-PM1:*** *Eu só quero curar porque está difícil compreender esta doença.*

***RP-PM5:*** *Quando comecei a adoecer disseram que era a minha filha que tem 18 anos, que já começou a namorar e já teve aborto, trataram mas não melhorei, essa é apenas uma doença só.*

1. **Como é que pode ser feito o aconselhamento para ajudar um paciente a seguir com o tratamento de TB?**

***RP-PM1:*** *Outros abandonam de verdade, levantam medicamentos e não tomam, deitam os comprimidos. É melhor que o tratamento seja no regime de internamento, dar injeções e comprimidos durante seis meses. Dar aviso que vai internar no hospital***.**

***RP-PM6:*** *O enfermeiro não tem nada para aconselhar, apenas pode deixar o doente.*

***RP-PH3:*** *O enfermeiro deve aconselhar os doentes a seguirem com as orientações do hospital, que ele vai mudar, vir no hospital fazer controlo, não fazer relações sexuais, não comer piri-piri e não beber.*

***RP-PM4:*** *Pedir os pacientes a não beber, aconselhar a continuar com o tratamento, vir sempre no hospital.*

**SECÇÃO C: ADESÃO AOS SERVIÇOS TB**

***(Geralmente é difícil para muitos pacientes aderirem ao tratamento TB,TB-MR e TB/ HIV).***

1. **Quais são os problemas que os doentes enfrentam para iniciar o tratamento com:**
2. **TB?**

***RP-PM5:*** *Muitos problemas é não-aceitação da doença, a pessoa vê que você está doente, está magrinha, mas é teimoso e não faz o tratamento. Outros dizem que essa doença não se cura no hospital, muitos dizem que o hospital está a fazer negócio.*

***RP-PM1:*** *Muitos não iniciam o tratamento porque confiam nos curandeiros, os curandeiros não motivam os doentes a fazer o tratamento no hospital porque dizem que o hospital não trata a TB. Esses curandeiros precisam de saber sobre essa doença e dirigir os doentes para o hospital.*

***RP-PH3:*** *Tem outros doentes que vivem longe. É necessário ir nas comunidades fazer palestras, fazer consultas nas brigadas móveis, levar os escarradores, distribuir aos doentes com tosse, caso saia resultado positivo, fazer a busca do doente, para ele iniciar o tratamento.*

***RP-PM4:*** *Outros têm dificuldade de passagem para alcançar o CS, com o tratamento.*

***RP-PM2:*** *Outros é por causa da distância, gostaria que os ativistas passassem nas zonas para distribuir medicamentos aos doentes que sofrem dessa TB. Nós estamos a vir sempre porque vivemos perto.*

***RP-PM4:*** *Eu vivo longe, mas quando me disseram que devo levantar análises, não fui para casa. Dormi em casa da minha família que fica perto daqui do hospital.*

***RP-PM2:*** *Eu vivo em Maguimba, mas quando me disseram que tenho essa doença aluguei uma casa perto do hospital para me diminuir a distância. Por exemplo em Metochira Pita é uma zona grande, precisamos de um hospital.*

1. **TB-MR?**

n/a

1. **TB- HIV?**

n/a

1. **Quais são os aspetos que foram mais difíceis para continuar a fazer o tratamento?**

***RP-PM6:*** *Nós não sabemos. Isto depende do comportamento de cada um. Há uma senhora que só levantou comprimidos uma vez e não veio mais.*

***RP-PM1:*** *Outros porque ainda não percebem bem sobre a doença. Quando recebem os comprimidos deitam e são os primeiros a conviver com os outros e espalham a doença. Há muito tempo vinham muitos doentes porque recebiam soja. Agora que não tem soja, aqui já não há nenhum doente.*

***RP-PH3:*** *Outros não continuam por causa da vergonha.*

***RP-PM4:*** *Muitos na verdade é por causa da vergonha, mas eu não tenho vergonha, porque eu é que estou doente.*

***RP-PM1:*** *Eu vivo no quarto bairro, muitos me riam em 2013, se eu fosse fraca não vinha cumprir o tratamento, mas as minhas vizinhas hoje estão a fazer o tratamento de HIV/SIDA.*

***RP-PH3:*** *A doença veio para todos, não veio para uma pessoa só, galinha, cabrito, todos animais adoecem.*

***RP-PM1:*** *Eu me riram muito em 2013. Me descriminavam. Nós aqui temos que mobilizar os outros para continuarem a tomar os comprimidos.*

***RP-PH3:*** *Outros não continuam porque não têm comida, esses comprimidos provocam muita fome e são muito fortes.*

***RP-PM3:*** *Esse remédio provoca muita fome, gostaria que o governo ajudasse com comida ou papa soja, porque este medicamento provoca muita fome.*

***RP-PM5:*** *Esses comprimidos provocam vertigem e fraqueza. Se você não for forte acabará desistindo.*

***RP-PM1:*** *Podemos não ter comida, mas devemos continuar a vir receber comprimidos.*

***RP-PH3:*** *O meu vizinho foi perguntado quantos filhos tem e ele disse que tem dez, enquanto tem só três. Isso é por causa da fome, para receber soja. Eu como muito.*

***RP-PM6:*** *Sinto muita fome, acordo com fome e fico muito cansada por causa dos comprimidos.*

***RP-PM4:*** *Uns levantam medicamentos e não tomam, mas bebem álcool.*

***RP-PM5:*** *Você pode desmaiar por causa da fome.*

***RP-PM2:*** *Eu como muito, esses comprimidos provocam muita fome.*

***RP-PM1:*** *Esses comprimidos obrigam a comer muito.*

**SECÇÃO D: MELHORAR O LABORATÓRIO E PNCT**

1. **Existe algo que poderia ser melhorado nos serviços de PNCT?**

***RP-PM2:*** *Por mim está tudo bem, estamos a ser bem tratados, gostaria que o estado continuasse assim.*

***RP-PM5:*** *Estamos a ser bem tratados, por isso não tem nada a ser melhorado.*

***RP-PM6:*** *Estão a tratar bem, nada poderia ser melhorado porque nós não estamos a pagar nenhum dinheiro.*

***RP-PM1:*** *Poderia se oferecer pelo menos soja para os doentes de TB, para prevenir a reação provocada pelos comprimidos.*

***RP-PH3:*** *Outros morrem pela fome.*

***RP-PM6:*** *Poderiam distribuir comida, sobretudo soja para os doentes de TB, para diminuir a reação adversa.*

***RP-PM1:*** *Gostaria que fosse feito um encontro com todos doentes com TB, para termos conhecimento sobre a doença, e nós podermos apoiar aqueles que não vêm ao hospital. Seria bom nós recebermos uma palestra sobre esta doença. Esta doença existe e muitos estão a morrer.*

***RP-PH3:*** *Quando morrem dizem que é feitiçaria, enquanto é doença simples.*

- 1. **O que deve ser feito pela US na seleção ao tratamento e sua continuidade?**

***RP-PM6:*** *Nós como estámos a iniciar, estámos a aprender apenas.*

***RP-PM1:*** *O tratamento de TB é só para seis meses. Quando termina pedimos ir fazer Raio X de controlo antes da alta. Para evitar andar muitas vezes, era bom diminuir a quantidade de comprimidos. Não dar alta aos doentes sem o diagnóstico terminar. Muitas vezes temos alta sem curar.*

***RP-PM2:*** *Para mim o meu diagnóstico foi feito pelo Raio X.*

***RP-PH3:*** *Gostaria que o tratamento fosse feito para ambos, isto é, com o casal para que o aconselhamento seja para o casal.*

***RP-PM2:*** *Eu fui chamar o meu marido para saber sobre o meu tratamento.*

***RP-PH3:*** *Nós jovens precisamos de manter relações sexuais. Muitas vezes as mulheres não acreditam quando o homem fala das recomendações do hospital. Eu durmo na sala e minha esposa no quarto. Quando ela não aceita eu digo que pode ir a casa dos pais, eu quero a minha cura.*

***RP-PM6:*** *Nós gostaríamos que nos dessem soja.*

***RP-PM1:*** *Gostaríamos de ter leite e soja.*

***RP-PM6:*** *Gostaríamos que quando o tratamento com comprimidos não melhora, nos fosse feito um Raio X do tórax, talvez a doença está no tórax.*

- 1. **O que o trabalhador de saúde poderia fazer para melhorar aderência ao tratamento?**

***RP-PM1:*** *Gostaria que nas segundas-feiras o enfermeiro de TB fizesse uma palestra a convidar todos os doentes com tosse para fazer análise, porque muitos chegam e não são bem dirigidos para o setor de TB. Gostaria também que os trabalhadores do setor de TB fossem a procura dos doentes com tosse nos centros de saúde periféricos para serem tratados.*

***RP-PH3:*** *Eu vim várias vezes, sempre me deram comprimidos de malária, isso porque eu não conhecia o setor de TB.*

***RP-PM5:*** *Eu sofri muito. Tomei muitos medicamentos de malária, mais tarde é que consegui localizar o setor de TB.*

***RP-PM1:*** *Eu estou viva por causa do vizinho que quando perguntei onde fica o setor de TB, ele me mostrou este setor.*

***RP-PH3:*** *Eu sofri muito. Fiz vai e vem. Os trabalhadores devem ir nas zonas para procurar doentes com tosse nos bairros.*

***RP-PM5:*** *Minhas amigas me riam, mas agora estou bem.*

***RP-PH3:*** *Queremos soja e verão que muitos vão engordar. O governo deve ajudar nisso que também ajudaria no aumento da aderência.*

***RP-PM1:*** *Eu sofri muito para iniciar o tratamento em Maputo. Eu chorei para os trabalhadores de saúde me fazerem o Raio X do tórax.*

***RP-PM6:*** *Para nós que estamos longe, gostaria que fossemos tratados lá porque não temos dinheiro de passagem*

1. **Acha que fazer o diagnóstico e tratamento imediato da tuberculose melhoraria o estado de saúde do paciente? *(Sondar: como? Ou de que maneira?*)**

***RP-PH3:*** *Iniciar o tratamento imediatamente é bom porque evita desgastar o organismo. É preciso atacar logo com o tratamento.*

***RP-PM6:*** *Outros iniciam o tratamento muito tarde, enquanto o corpo está bem acabado e é difícil passar a doença.*

***RP-PM5:*** *É bom imediatamente iniciar o tratamento porque quando demoram a doença vai curar lentamente, não é fácil tratar a TB. Quanto mais tarde for, mais lenta será a cura da doença.*

***RP-PM6:*** *A doença é preciso tratar imediatamente para os pulmões não se estragarem tanto.*

***RP-PM1:*** *Gostaríamos que aumentassem o número de enfermeiros que fazem a triagem para melhorar o diagnóstico de TB. Outros não sabem que aqui se faz o tratamento de TB. Esses enfermeiros do setor de TB devem ir a triagem procurar pessoas com tosse.*

***RP-PH3:*** *Eu fiquei um ano a vir para aqui a me darem comprimidos brancos e não curava.*

***RP-PM5:*** *Eu sofri muito.*

***RP-PM1:*** *É preciso fazer o tratamento todos os dias e o diagnóstico deve ser feito na triagem. Todos os doentes não conhecem o setor de TB. Muitos estão a morrer por não saberem a existência deste setor.*

- 1. **Acha que fazer o teste de HIV e iniciar o TARV melhoraria o estado da vida do paciente? Explique?**

***RP-PM1:*** *Outras pessoas devem fazer os dois tratamentos, TB e HIV.*

***RP-PH3:*** *Sim melhoraria, mas é importante que o tratamento inicie cedo para o casal. Não deve ser feito o tratamento individualmente.*

***RP-PM6:*** *Também devem dar aconselhamento para usar o preservativo.*

***RP-PH3:*** *Em Morrumbala muitas pessoas e sobretudo crianças estão a apanhar o tratamento de HIV. Eu não sei onde contraíram a doença.*

***RP-PM5:*** *Outras crianças apanham tratamento de HIV, apanharam a doença quando a mãe esteve grávida.*

***RP-PH3:*** *É bom porque iniciou cedo com o tratamento.*

***RP-PM6:*** *Sim melhoraria, porque ir sozinha a andar muitos não vão descobrir que você esta doente, muita gente não vai conhecer a minha doença.*

***RP-PM5:*** *Muitos não acreditam que essa doença existe, dizem que os enfermeiros estão a mentir, esta doença não existe.*

***RP-PM4:*** *É vergonhoso vir ao hospital enquanto está mal doente. Se vir cedo é melhor.*

***RP-PM5:*** *Esse tratamento exige comer. Se você tomar remédio passa mal e pode causar a morte.*

***RP-PM1:*** *Esta doença se você seguir marido pode ter divórcio, se seguir mulher pode ser divorciada, é uma doença complicada.*

***RP-PH3:*** *Onde eu trabalho ele não aceita o tratamento, quando lhe convidamos para vir ao hospital não aceita, quando você lhe diz para vir ao hospital até te insulta, mas sempre compra medicamentos no mercado.*

***RP-PM6:*** *A pessoa demora ir ao hospital porque não sabe o que está a sentir, deve-se ir ao hospital para melhorar*

***RP-PM1:*** *É um senhor que conheço que vende aí no mercado, fica parece boss, mas está doente. É importante vir ao hospital, porque é uma estrutura que tem conhecimento da ciência.*

1. **Tem mais alguma coisa a acrescentar sobre o que já discutimos?**

***RP-PM1:*** *Tudo o que falamos gostaria que acontecesse em 2016.*

***RP-PM6:*** *Acontecerem as nossas palestras aqui no HRN. Os doentes de HIV e TB, somos irmãos, gostaria que estivéssemos juntos no encontro.*

***RP-PH3:*** *Gostaria que o que falamos acontecesse.*

**MUITO OBRIGADO (A) Hora do fim da entrevista__12:45_**
