# Supplementary material for: Barriers to access and adherence to tuberculosis services, as perceived by patients: A qualitative study in Mozambique
Source: PLoS One. 2019 Jul 10;14(7):e0219470. doi: 10.1371/journal.pone.0219470 (PMC6619801; doi:10.1371/journal.pone.0219470)
Supplement: S1 Dataset — (ZIP) [file pone.0219470.s003.zip › Transcripts TB study/DGF6_.docx]

**"Avaliação da Cascata de Cuidados de Pacientes Diagnosticados com TB, MDR-TB e Paciente Co-infectados com TB/HIV nas Províncias de Manica e Sofalaʺ**

# Instrumento: Guião De Entrevista para Grupos Focais - DGFs

**Data:** 18.02.2016

**Distrito:** Nhamatanda

**Nome da Unidade Sanitária**: HRN

**Hora do início:** 08H:59

**Hora do fim:** 11H:05

**Número de DGF:** 06

**Legenda**

**E:** Pergunta do(a) Entrevistador(a)

**P:** Participante/entrevistado(a)

**RP:** Resposta do(a) Participante/entrevistado(a)

**PH:** Participante Homem (seguido de sua posição de assento)

**PM:** Participante Mulher (seguida de sua posição de assento)

**n/a :** Não Aplicável

| Comentários/Observações Preliminares: *(circunstâncias que poderão influenciar a entrevista, etc.)* *A DGF correu bem. Foi feita na varanda da enfermaria de TB, um local aberto, e participaram três pessoas, duas do sexo feminino e um do sexo masculino. No final foi oferecido um lanche aos participantes.* |
| --- |

**SECÇÃO A: ASSISTÊNCIA DO SERVIÇO DE SAÚDE AOS PACIENTES COM TB, MR-TB E TB-HIV**

1. **O que você sabe sobre TB?**

***RP-PM2:*** *TB é uma doença que se transmite de uma pessoa infetada para outra, através de tossir para outras pessoas sem tapar a boca, e comer no mesmo prato. É preciso tossir num plástico com areia e pôr o escarro no frasco, lavar muito bem o frasco de escarro e secar no sol.*

***RP-PH1:*** *TB é uma tosse e aquecimento no peito. Vim aqui, disseram-me para fazer análise. Fiz e disseram-me que estou com TB. Já estou a fazer o tratamento com injeções há quatro meses, por vezes sinto muita dor do meu corpo, por vezes o corpo me doi e tenho formigueiro.*

1. **O que você sabe sobre TB- MR?**

***RP-PH1:*** *Esta TB-MR que tenho eu não sei o que é, porque não nos explicaram, apenas os comprimidos que estou a tomar são diferentes com dos outros doentes. Eu estava a tomar 16 comprimidos, mas agora depois do peso diminuiram para 12 comprimidos e injeções. Por vezes sinto muita comichão no corpo.*

***RP-PM2:*** *TB-MR é andar fora com um homem sem saber que ele tem os seus problemas de saúde como tosse, aquecer o corpo, e aquecimento dos pulmões. Aí se eu me envolver com esta pessoa posso apanhar esta TB-MR.*

1. **O que acha sobre os serviços prestados neste sector de TB?**

***RP-PM2:*** *Os serviços prestados estão bem porque quando cheguei aqui no hospital não conseguia andar nem ficar de pé, mas agora já estou bem melhor. Apenas quando tomo os medicamentos tenho tido dores de cabeça, vertigem, e por vezes não consigo andar.*

***RP-PH1:*** *Estes serviços estão a andar muito bem, não temos tido dificuldades. Logo que chegamos os enfermeiros nos atendem bem, mesmo chegando tarde atendem-nos*

1. **Algum dia teve qualquer dificuldade durante o processo para acesso aos serviços de TB, TB-MR? Explique.**

***RP-PM2:*** *Tive dificuldades porque comecei com pontadas. Vim para aqui fazer consulta, deram-me medicamentos e não melhorei, voltei pela segunda via, também deram-me outra receita e não melhorei, a terceira via foi quando me deram frasco para fazer análise. Fiz as análises de tosse, foi quando descobriram a minha doença e me enviaram para este setor.*

***RP-PM3:*** *Sim tive dificuldades, vim para aqui fizeram-me análise de TB, acusou e comecei a fazer o tratamento de TB.*

1. **O que sabe sobre HIV?**

***RP-PH1:*** *HIV é uma doença que é provocada com a pessoa que tem HIV e quando mantem as relações sexuais, mas quando toma medicamento a pessoa melhora.*

***RP-PM2:*** *HIV é uma doença provocada por relações sexuais, agulhas infetadas com o virus de HIV, lâminas no curandeiro, quando cortam uma pessoa infetada e depois a mesma passa para uma pessoa que não tem esta doença.*

***RP-PM3:*** *HIV é uma doença que se apanha quando você se descontrola nas relações sexuais, injeções infetadas, e lâminas infetadas. Essa doença tem tratamento, quando cumpre melhora a saúde.*

1. **O que foi mais dificil em compreender sobre TB e TB-MR?**

***RP-PH1:*** *Outros dizem que TB é provocada quando você varre a casa com janelas e portas fechadas, fazer fornos de carvão e trabalhar muito esforçado de baixo do sol. Está difícil entender essa interpretação.*

***RP-PM2:*** *É difícil entender. A poeira provoca TB. A pessoa quando varrer deve fechar a boca.*

***RP-PM3:*** *É difícil entender que trabalho esforçado provoca TB. Manter relações sexuais provoca T*

1. **Como é que pode ser feito o aconselhamento para ajudar um paciente a seguir com o tratamento de TB?**

***RP-PM2:*** *Os enfermeiros acompanham-nos bem, mas os doentes devem ajudar e ouvirem o que nos aconselham, se não acatar esta informaçao você é que fica prejudicado.*

***RP-PH1:*** *O enfermeiro deve ajudar dizendo o paciente a não faltar no tratamento. Para melhorar a saúde você que é doente deve cumprir com o que o enfermeiro te diz. O enfermeiro também deve fazer análise de controlo. Deve aconselhar a não faltar no tratamento, se não, não vai curar.*

***RP-PM2:*** *Enfermeiros aconselham a vir no hospital sempre. Fazer injeções todos os dias, mas os que não obedecem não curam. Também devem aconselhar a tomar os comprimidos que levantam no hospital.*

***RP-PM3:*** *Muitos doentes levantam os comprimidos e não tomam. Na verdade estes não vão curar e dão culpa aos enfermeiros. Os enfermeiros devem obrigar os doentes a tomarem os comprimidos para melhorarem a sua saúde.*

**SECÇÃO C: ADESÃO AOS SERVIÇOS TB**

***(Geralmente é difícil para muitos pacientes aderirem ao tratamento TB,TB-MR e TB/ HIV).***

1. **Quais são os problemas que os doentes enfrentam para iniciar o tratamento com:**
2. **TB?**

***RP-PM2:*** *Muitas das vezes tem sido teimosia dos doentes, não acatam o conselho do pessoal de saúde.*

***RP-PH1:*** *Outros quando iniciam o tratamento durante três meses e não melhoram abandonam porque não acreditam no tratamento que estão a fazer, uma vez que não estão a registar melhorias. Quando voltam a US os enfermeiros explicam a reação do medicamento e o tempo de tratamento que é longo. Não iniciam o tratamento dependendo do comportamento de cada individuo, eu quando descobriram a minha doença logo comecei a fazer o tratamento. Todos os dias apanho injeções. Todas as pessoas devem cumprir, outros não confiam nas máquinas do hospital e gostam de ir ao curandeiro, e quando vão lá também não curam. Outros acham que a doença que têm é feitiço. É importante seguir o hospital porque na verdade cura a doença.*

***RP-PM3:*** *Os que não iniciam o tratamento é por sua culpa. Muitos é por causa da vergonha de vir receber os comprimidos, temem ser gozados com os vizinhos. Muitos têm receio das pessoas e de serem vistos que estão a fazer tratamento de TB.*

***RP-PH1:*** *É melhor vir tomar medicamentos porque vergonha mata. Algumas pessoas dizem ter medo de serem descobertas que estão a fazer tratamento de TB.*

1. **TB-MR?**

***RP-PH1:*** *Depende de cada pessoa, muitos não acreditam na doença, têm vergonha dos vizinhos e da estigmatização. É bom cumprir o que o hospital fala. Quando cumpre, você na verdade cura. Eu sempre venho aqui no hospital e estou a curar. Outros por causa da teimosia chegam aqui no hospital muito tarde, já acabados e são obrigados a baixar por decidirem vir tarde no hospital. Isto tudo por causa da vergonha dos vizinhos. Tenho meu vizinho, que por causa do receio chegou no hospital muito tarde e baixou durante quatro meses, mas agora já esta melhor, e já teve alta e está em casa.*

***RP-PM2:*** *Estas pessoas não iniciam por falta de bom comportamento. Nem o enfermeiro quando te fala é o bem para você. Nem os que te riem é melhor os deixar porque eles na verdade não estão bem, você só tem que cumprir com o tratamento para melhorar a saúde, depois eles vão se envergonhar.*

***RP-PM3:*** *Os que não iniciam com o tratamento é pelo seu comportamento, por vezes primeiro querem ir no curandeiro, mas estão a perder o seu tempo, em vez de vir receber tratamento.*

***RP-PH1:*** *Estão a perder tempo, o tratamento é gratuito.*

1. **TB- HIV?**

*n/a*

1. **Quais são os aspetos que foram mais difíceis para continuar a fazer o tratamento?**

***RP-PH1:*** *Não continuam porque a cura desta doença é muito lenta. Eles querem curar logo. Outros desistem porque vão nos outros hospitais, assim como nos curandeiros e não querem cumprir com o tratamento. Se você não cumpre não cura, é preciso vir sempre no hospital até você ser tratado todos os dias, e até o enfermeiro pedir outra análise para ver a sua saúde. Outros não continuam porque a doença proíbe muitas coisas como manter relações sexuais, comer piripíri e fazer machamba. Outros não conseguem cumprir e acabam desistindo o tratamento assim que sentem uma pequena melhoria.*

***RP-PM2:*** *Muitas vezes eles tomam medicamentos por duas a três semanas, se não registarem melhorias, eles desistem do tratamento porque dizem que os medicamentos não estão a fazer nada no seu organismo. Outros é por causa da reação dos medicamentos.*

***RP-PM3:*** *TB é diferente de outras doenças como dores de barriga e malária. TB tem um tratamento muito forte, quando começa o tratamento, os comprimidos provocam muita reação, os comprimidos partem todo corpo, não consegues levantar nem comer, se o doente não for forte pode desistir. A reação destes medicamentos dura um mês, no segundo mês você pode sentir menos reações adversas*

***RP-PM2:*** *Devem cumprir pelo menos um mês a tomar o remédio.*

***RP-PH1:*** *Eu quando tomo os comprimidos tenho tido muitos problemas sobretudo vertigem. Para chegar em Chirassicua tenho que sentar várias vezes pelo caminho.*

***RP-PM2:*** *Eu depois de tomar os comprimidos e apanhar injeções sofro muito, a minha família me põe água na cabeça.*

***RP-PM3:*** *Esta doença é muito complicada. Provoca muita reação. Sem coragem você pode desistir.*

**SECÇÃO D: MELHORAR O LABORATÓRIO E PNCT**

1. **Existe algo que poderia ser melhorado nos serviços de PNCT?**

***RP-PH1:*** *Neste setor os serviços estão a correr bem, mesmo chegando tarde a situação é a mesma. Apenas o setor gosta que os doentes cheguem cedo. Poderia o setor nos apoiar com comida para distribuição aos doentes com TB porque este medicamento provoca muita reação adversa.*

***RP-PM2:*** *Esses comprimidos quando tomamos devemos comer alguma coisa para pôr no estomago.*

***RP-PM3:*** *Outros só reclamam sobre comida, o setor devia nos apoiar com papa. Pelo menos outros setores recebem comida, mas nós não. Não recebemos nada e a nossa doença não nos permite trabalhar. Os nossos companheiros recebem arroz, açúcar, sabão, sabonete, óleo, e etc.*

- 1. **O que deve ser feito pela US na seleção ao tratamento e sua continuidade?**

***RP-PH1:*** *Antes recebia 16 comprimidos, mas depois do peso passei a receber 12, mas tem sido muito difícil porque sinto muita dor da caixa torácica. Não temos como contrariar a seleção do medicamento, só queremos a melhoria da nossa saúde.*

***RP-PM2:*** *A seleção do medicamento está bem porque eu quando cheguei aqui não conseguia andar, nem ficar de pé, mas agora depois deste tratamento já me sinto melhor, apenas lamentar sobre as reações adversas, depois de tomar sinto meu corpo todo partido e sem força. Fico totalmente grossa, só me põem água na cabeça.*

***RP-PM3:*** *A seleção do medicamento está bem e depende da gravidade da doença, primeiro comecei com 16 comprimidos, mas depois foi reduzindo, mas curei.*

- 1. **O que o trabalhador de saúde poderia fazer para melhorar aderência ao tratamento?**

***RP-PM3:*** *O trabalhador de saúde deve ter muita paciência com os doentes, outros insultam quando o paciente não responde a chamada. O trabalhador de saúde deve ter respeito com os doentes.*

***RP-PM2:*** *O trabalhador deve escutar bem a doença dos doentes para fazer um bom diagnóstico. Por vezes antes do doente terminar de falar o enfermeiro já tem a receita para o paciente. As vezes o medicamento não é da sua doença. Tem de ter paciência.*

***RP-PH1:*** *Enfermeiro é um pai, deve atender o doente como o seu filho, deve ter paciência, o doente deve falar o que sente e ser dado o medicamento que corresponde com a sua doença. O trabalhador deve ter paciência com todos os doentes, deve seguir a bicha segundo a ordem de chegada, não atender primeiro amigos e você que chegou primeiro permanecer ainda na bicha. O enfermeiro deve atender com pena.*

1. **Acha que fazer o diagnóstico e tratamento imediato da tuberculose melhoraria o estado de saúde do paciente? *(Sondar: como? Ou de que maneira?*)**

***RP-PH1:*** *É bom porque melhora a saúde do paciente antes de ficar desgastado, o doente não faz muitas voltas para US, ajuda ao doente porque ele não vai andar muito.*

***RP-PM2:*** *Sim, melhoraria a saúde do paciente porque você começa cedo o tratamento, antes que a pessoa fique muito fraca, porque quando você fica muito fraca, a cura torna-se lenta.*

***RP-PM3:*** *Sim melhoraria a saúde do paciente, porque o doente recupera rapidamente.*

- 1. **Acha que fazer o teste de HIV e iniciar o TARV melhoraria o estado da vida do paciente? Explique?**

***RP-PH1:*** *Sim melhoraria a saúde do paciente porque o doente vive muito tempo, basta cumprir com o tratamento.*

***RP-PM2:*** *Sim melhoraria o estado de saúde do paciente, porque você inicia logo o tratamento, o estado de saúde é bom, vive mais tempo do que a pessoa que não vem no hospital. Não devemos ter vergonha com as pessoas, porque a vergonha mata.*

***RP-PM3:*** *Sim é melhor tratar cedo antes de desgastar a saúde. O seu estado de saúde melhora em relação àquele que não inicia e vive mais tempo de vida.*

1. **Tem mais alguma coisa a acrescentar sobre o que já discutimos?**

***RP-PM2:*** *Por minha parte não tenho nada por acrescentar.*

***RP-PH1:*** *O que foi discutido aqui é tudo, já não tenho nada.*

***RP-PM3:*** *Apenas pedir a vocês para que nos ensinem sobre esta doença de TB, como se apanha porque até aqui não temos conhecimento.*

**MUITO OBRIGADO (A) Hora do fim da entrevista___11H:05__**
