# Supplementary material for: Barriers to access and adherence to tuberculosis services, as perceived by patients: A qualitative study in Mozambique
Source: PLoS One. 2019 Jul 10;14(7):e0219470. doi: 10.1371/journal.pone.0219470 (PMC6619801; doi:10.1371/journal.pone.0219470)
Supplement: S1 Dataset — (ZIP) [file pone.0219470.s003.zip › Transcripts TB study/DGF7_.docx]

**"Avaliação da Cascata de Cuidados de Pacientes Diagnosticados com TB, MDR-TB e Paciente Co-infectados com TB/HIV nas Províncias de Manica e Sofalaʺ**

# Instrumento: Guião De Entrevista para Grupos Focais - DGFs

**Data*:*** *08/03/16*

**Distrito:***Chimoio*

**Nome da Unidade Sanitária**:*C.S.E.M*

**Hora do início:***09:08*

**Hora do fim:** *10:52*

**Número de DGF:***07*

**Legenda**

**E:** Pergunta do(a) Entrevistador(a)

**P:** Participante/entrevistado(a)

**RP:** Resposta do(a) participante/entrevistado(a)

**PH:** Participante Homem (seguido de sua posição de assento)

**PM:** Participante Mulher (seguida de sua posição de assento)

**n/a :** Não Aplicável

| Comentários/Observações Preliminares: *(circunstâncias que poderão influenciar a entrevista, etc.)Comentario a entervista foi feita na sala de PNCT com grupo focal de 5 participante e foram muitos participativos com as questoes.* |
| --- |

**SECÇÃO A: ASSISTÊNCIA DO SERVIÇO DE SAÚDE AOS PACIENTES COM TB, MR-TB E TB-HIV**

1. **O que você sabe sobre TB?**

**RE- PM1**:*Sobre TB è uma tosse em que uma pessoa tosse sangue,e tambem tosse escaro com sangue.E quando vem aqui no hospital faz analise acusa T.B e na nossa tradição acontece isso quando não compriu com as cerimonias do falecimento.Se voce não comprir com aquelas todas cerimonias tradicionais de falecimento é quando acontece essa tosse,e se você for tratar tradicionalmente é quando diz que é phiringanisso,mas aqui no hospital quando faz analise é quando acusa TB.*

**RP-PM3**: *TB é uma doença em que uma pessoas com sintomas de frio todo corpo,e ao tossir deve tapar a boca,TB são bacilo de tosse durante uma semana e isso que e o meu ponto de vista sobre TB.*

**RP-PM4**:*TB, é uma doença que pode transmitir atrávez de poeira,tradicionalmente diz que é phirringanisso,começa a tossir e ao tossir deve evitar muito tossir onde estão as criancas por causa desse bacilos, e podemos até contaminar outra pessoa, devemos comprir com o tratamento.*

**RP-PM5**:*TB, é uma doença que pode se apanhar naquelas cerimonias de falecimento e se não comprir com as cerimonias de falecimento pode causar TB, no futuro por causa de não comprir aquelas cerimonias de falecimentos e quando comessa a tossir é quando vai ao curandeiro e ele diz que é phirringanisso, aqui no hospital quando vem fazer analises acusa TB.*

1. **O que você sabe sobre TB- MR?**

**RP-PH2:** *TB-MR é aquela TB, que você apanha phirringanisso e não cumpre com os tratamentos e quando apanha recaida e vem aqui nos hospital diz-se que é TB-MR. faz analise e logo inicia com tratamento de injecção e comprimidos e não deve falhar com esse tratamento. Agora você vem aqui no hospital ja acabado, doente e não cumpre com as regras do hospital é dificil de melhorar.*

**RP-PM2**: *TB-MR é aquela TB em que um doente não cumpre com as regras do hospital e somos falado para não fazer relação sexual enquanto esta em tratamento ,não devemos comer peixe seco. Essa TB-MR, aparece quando não cumpre com as regras de hospital, um doente deve cumprir com tratamento e regra do hospital*.

**RP-PH2**: *Ao tossir deve tapar a boca ,porque tem outros com TB que fica no osso, eu primeiro vim aqui fiz analises de TB e cumpri 6 meses de tratamento mas até agora ainda estou a tossir só, mas estou a fazer tratamento não sei se essa TB está no osso. Ja não sei também porque não está a passar está sendo complicado para mim .*

**RP-PM1**:*Não sei de nada sobre essa TB-MR.*

1. **O que acha sobre os serviços prestados neste sector de TB?**

**RP-PH2:***Os serviços prestados neste sector do principio até hoje estamos a ser tratado muito bem. medicamento estou a tomar até ja estou a me sentir melhor porque quando vim aqui no hospital estava muito mal e muito doente, mas agora ja estou melhorando. Somos aconselhado a não fazer relação sexual, enquanto está em tratamento e somos dado um bom aconselhamento que olhar primeiro a toma dos medicamento e depois é quando pode comer algumas coisa tambem somos aconselhado não devemos fazer relação sexual enquanto está em tratamento, e estamos e ser bem tratado e temos um aconselhamento com tratamento de injeção para aqueles que apanha injeção são picadoe só descansam no sabado e domingo durante o fim de semana.e todos os dias temos tratamento e somos dados medicamento ,embora o enfermeiro tem chegado tarde mas temos atendimento apesar de ser tarde.Gostaria que melhora-se essa parte de atendimento porque não está ajudar porque o enfermeiro diz para nos chegarmos cedo e ele vem muito tarde .Porque alguns doente desarascam, fazemos buscato para ter comida ,agora o enfermeiro chega tarde, a que hora nos vamos fazer nossos biscatos para comprar nossa comida porque ficamos quase todo dia aqui a espera de tratamento. Agora isso não ajuda cria transtorno para um doente.Quando reclamamos ele diz que olha saúde é sua não há como, devemos comprir sempre.*

**RP-PM3**: *Os serviços estão a andar bem, cada doente que vem sempre aqui levanta seu medicamento toma e vai a casa sem problema. Só de lamentar essa coisa de atrazo do atendiemento,o enfermeiro chega aqui 9 horas é quando começa a dar medicamentos, agora o doente fica aqui muito tempo a espera e cansado de esperar e comeca a ter vertins por causa de fome. Somos falado que devemos vir antes de pôr nada na barriga então isso é complicado para um doente.Porque esse enfermeiro chega aqui muito tarde.*

**RP-PM1**: *Os serviços prestados estão bem, só de lamentar essa parte de atrazo no atendimento.O enfermeiro fala para nos chegarmos muito cedo e ele chega aqui muito tarde e isso não da para doente. Gostaria que pelo menos que inicia-se atender 8 hora iniciar a dar medicamento mas ele começa com trabalho dele 9 horas. Mas para parte de medicamento ele da sem problema só lamentamos essa coisa de atrazo no atendimento. Por exemplo injecção ele inicia 11 horas porque primeiro atende os doentes que recebem medicamento de comprimido.*

**RP-PH2**: *Há muito tempo atendiam 2 pessoas ,mas agora só ficou uma pessoa para o atendimento não sei porque .*

**RP-PM5**: *Os serviços estão bem, só que essa coisa de atrazo no atendimento,ele primeiro quando chega aqui atende filhos deles coisa de casa ,depois de atender filhos é quando atende a nós também,lamentar atrazo no atendimento.*

**RP-PM4**: *Os serviços prestados está tudo bem, só de lamentar essa parte de atrazo no atendimento ,porque ele diz devemos chegar aqui cedo,mas ele chega muito tarde enquanto nos saimos cedo de casa sem comer nada para chegarmos aqui ficar muito tempo e para sair muito tarde por causa de demora no atendimento.*

1. **Algum dia teve qualquer dificuldade durante o processo para acesso aos serviços de TB, TB-MR? Explique.**

**RP-PH2:** *Durante o processo aos serviços de TB,TB-MR sim tive muitas dificuldades porque eu primeiro não sabia que era TB, primeiro ia nos curandeiros mas não curava essa tosse,Depois de um tempo é quando fui ao hospital e fui dado frasco para cospir e fiz analises ,e saiu resultado positivo de TB, e dai iniciei com tratamento de tuberculose e ja estou sentir melhor.Mas quando você cumprir com tratamento aqui no hospital você melhora com* *esse tratamento*.

**RP-PM3**: *Durante os serviços eu tive dificuldade, porque minha tosse não passava .Assim estou pensar em ir para curandeiro para ver o que esta acontecer com essa problema de tosse porque não esta a passar.*

**RP-PM4**: *Durante o processo o serviço para acesso de TB eu não tive dificuldade mesmo no laboratorio não tive problema.*

1. **O que sabe sobre HIV?**

**RP-PH2:***Sobre HIV,apanha-se atravez de agulha,relação sexuais não protegida..*

**RP-PM3**:*HIV,apanha se atravez de relação sexual não protegida,agulha e lamina.*

**RP-PM4**:*HIV apanha- se atravez de juntar com homem que HIV sem proteção,lamina,que cortou uma pessoas que tem HIV,agulha que usou uma pessoas que tem HIV.*

**RP-PM5***:HIV,apanha-se atravez de relacao sexual sem protecao ,nao usar lamina que usou outra pessoa que tem HIV.*

**RP-PH2**:*HIV e uma doenca que tem uma pessoa e outra pessoa vai manter relacao sexual com esta pessoa sem protecao pode transmitir HIV..*

1. **O que foi mais dificil em compreender sobre TB e TB-MR?**

**RP-PM4:***O mais dificeis em compreender sobre TB e TB-MR um enfermeiro deve explicar ao paciente o que e TB e TB-MR,mas para mi nao foi tão dificil em compreender essa doença.*

**RP-PM5**:*Para mi foi facil em compreender porque meu marido perdeu a sua esposa e eu fiquei preocupada porque nos eramos duas esposas e eu sou a segunda esposa,mas quando ele o marido chegou em casa informo-me que olha a outra esposa perdeu a vida porque tinha TB,depois dela ter morrido o marido informou-me,e eu iniciei a ficar doente .Quando começo ficar doente pensei muita coisa acerca daquilo que o marido havia me informado. Fui até ao curandeiro não resolvia nada comecei a vomitar sangue,fui mas ao outro curandeiro eu estava pensar que era fetico de familia*.

**RP-PM1**:*Não tive dificuldade porque meu pai tinha mesmo problema de TB agora foi facil de compreender esse problema de TB,e TB-MR.*

**RP-PM3**:*Nao tive problema de compreenser porque minha mae tinha mesmo problema de TB,e acabou morrendo com esse problema de TB,minha mãe tossia sangue e por isso não tive problema de compreender .Agora não sei se apanhei porque minha mãe tinha mesmo problema não sei,porque mesmo essa filha que eu tenho ja teve mesmo problema de TB fez tratamento e melhorou.*

1. **Como é que pode ser feito o aconselhamento para ajudar um paciente a seguir com o tratamento de TB?**

**RP-PH2:***Aconselhamento como um paciente e so dizer olha deve apanhar tratamento , um médico deve dar exemplos com uma pessoa que ja sofreu da doença de TB,falar para outros doente que olha esse paciente tinha mesmo problema mas agora ja esta melhor.Um enfermeiro deve atender aos pacientes com tempo e hora,porque e cansativo vir aqui todos os dias apanhar tratamento.Mas algumas pessoas tem vergonha de vir aqui no hospital por isso acaba abandonando pelomenos 2,3 dias injecção agora todos os dias voce aranjar alguem para dar injecção e pagar em vez de vir aqui no hospital nem se paga.*

**SECÇÃO C: ADESÃO AOS SERVIÇOS TB**

***(Geralmente é difícil para muitos pacientes aderirem ao tratamento TB,TB-MR e TB/ HIV).***

1. **Quais são os problemas que os doentes enfrentam para iniciar o tratamento com:**
2. **TB?**

**RP-PM5**: *Os aspecto mas dificeis para continuar a fazer tratamento e na fase inicial e só cumprir com todo tratamento, porque há outros paciente quando chegam no meio de tratamento e quando se sente ja melhor, pensam que já estão curados e abandonam o tratamento. Alguns doentes que ja estavam a tomar um tipo de medicamento num dia e no dia.quando chega aqui ha vezes em que da medicamento diferente trocam de medicmento isso cria transtorno para nos porque ja abetuamos um tipo de medicamento e para dia seguinte voce chega aqui e dado outro tipo de medicamento.Agora injecao voce deve vir todos os dias apanhar e para andar e grande problema serio e obriga para vir aqui todos os apanhar injecao ,mas estamos a comprir fazer oque e sua saude que esta em jogo deve cumprir.*

**RP-PH2:***Os problema que um doente enfrenta,voce encontra alguem que ja teve tratamento desencorraja diz que e dificil de comprir esse tratamento a dosagem e muito forte e esse tratamento provoca muita fome.e esse tratamento tem muita reacoes pricipalmente nos joelho doe para sentar e problema ,para fazer xixi sai xixi vermelho e pessoa ate fica com medo para continuar a tomar esse medicamento.*

**RP-PM5**:*Quando ver que tem problema de tossir sangue vai ao hospital fazer analises nao deve recuzar de ir ao hospital com sua familia.Em vez de esperar de ficar muito doente nao um bom e processo atrazado.Ha doente em que quando estao doente primeiro vao ao curandeiro perder tempo depois e quando ver que ja esta mal e quando vai ao hospital,e quando chega no hospital faz analise e acusa TB,enquanto ja esta muito mal.Nao devemos esperar ficar muito doente acabado e melhor ir ao hospital enquanto e cedo.*

**ii) TB-MR?**

*Nao aplicvel*

1. **TB- HIV?**

*Nao aplicavel*

**9.Quais são os aspectos que foram mais difíceis para continuar a fazer o tratamento?**

**RP-PM5**:*Os aspecto mas dificeis para continuar a fazer tratamento e na fase inicial e so cumprir com todo tratamento,porque ha outros paciente chegado no meio de tratamento quando se sente ja melhor......ja estava.....no dia seguinte.....quando chega aqui ha vezes em que da medicamento diferente trocam de medicmento isso cria transtorno para nos porque ja abetuamos um tipo de medicamento e para dia seguinte voce chega aqui e dado outro tipo de medicamento.Agora injecao voce deve vir todos os dias apanhar e para andar e grande problema serio e obriga para vir aqui todos os apanhar injecao ,mas estamos a comprir fazer oque e sua saude que esta em jogo deve cumprir.*

**SECÇÃO D: MELHORAR O LABORATÓRIO E PNCT**

1. **Existe algo que poderia ser melhorado nos serviços de PNCT?**

**RP-PH2:***Sim nesse sector de PNCT deve ser melhorado apartir de la no laboratorio ate aqui no PNCT,temos sofrido muito mesmo com receita para ser dado papel de resultado no laboratorio e um grande problema.Agora aqui na PNCT,tem grande problema de atrazo no atendimento,gostaria que pelomenos trabalha-se 2 pessoa porque esse enfermeiro nao esta a cumprir com as leis nem mandamentos.*

**RP-PM2***:Devem melhorar essa coisa de atrazo no atendimento e la tambem no laboratorio essa coisa de mandar ir em gondola diz vem amanha,voce chega aqui diz vem amanha,ate um doente chega de ir sozinho ate la em gondola ir saber o seu resultado isso nao e bom para um paciente.*

**RP-PH2**:*Eu dancei muito era falado vem amanha,vem amanha,amanha,amanha.*

**RP-PM1**:*Eu levei um mes para ter meu resultado.*

**RP-PM3**:*Essa coisa de atrazo no atendimento deve ser melhorado.*

**RP-PM4**:*O enfermeiro ele recomenda aos pacientes chegar cedo ,mais ele chega muito tarde e comeca atender mito tarde isso nao esta dar para os doentes, enquanto ele recomenda que devemos chegar aqui muito cedo e ficamos a espera dele.E ele inicia atender 9 hora isso cria transtorno para pacientes e saimos aqui no hospital muito tarde com fome,e vertins porque ele inicia atender muito tarde,deveria melhorar essa parte de atrazo no atendimento.*

- 1. **O que deve ser feito pela US na selecção ao tratamento e sua continuidade?**

**RP-PH2:** *A unidade sanitaria na selecao ao tratamento e sua continuidade deveria fazer palestras,e fazer que os pacientes testemunhar que olha eu tinha esse problmas assim assim assim, aranjar um espaco para fazer essas palestra todos os dias .Os paciente a dar testemunho aos outros doentes que olha eu esta muito mal doente mas com esse tratamento no hospital x mas ja estou melhor por causa desse medicamento.Esse e o meu ponto de vista a U.S deveria fazer isso que acabei de falar palestra.*

**RP-PM5**:*Assim ha doente chega tarde aqui no hospital bem mal acabado de doenca isso nao e bom.E muito bom um doente enquanto estiver andar ir ao hospital enquanto esta andar fazer analise e iniciar com tratamento cedo isso bom.Agora nada de esperar acabar corpo depois e quando vai ao hospital isso nao e bom,mas tambem esse tratamento e muito forte as reacoes doe joelho,e voce todos os dias deve ir apanhar tratamento,mas se voce comprir voce melhora.*

- 1. **O que o trabalhador de saúde poderia fazer para melhorar aderência ao tratamento?**

**RP-PM1:***O trabalhador de saude poderia ter amor com pacientes ,atender bem aos pacientes,porque ha outras enfermeira ,servente acompanhar aos doentes nas porta porque quando chegamos aqui,ha vezes em que chegamos aqui somos ensultados ser atendidos mal isso para doente nao e bom, um doente sente-se mal e ate pode abandonar tratamento .*

**RP-PH2**:*O trabalhador de saude deve atender muito bem aos pacientes.Porque se atende mal ate os paciente pode falar mal que olha aquela unidade sanitaria atende muito mal,mas no primeiro de maio atende muito bem aos pacientes,ha outros paciente que diz em nhamaonha e que atende bem aos pacientes.Um enfermeiro geral deve ter modo como receber os paciente e como expressar com paciente.*

1. **Acha que fazer o diagnóstico e tratamento imediato da tuberculose melhoraria o estado de saúde do paciente? *(Sondar: como? Ou de que maneira?*)**

**RP-PM1**:*O diagnostico imediato o tratamento de TB,sim e melhoraria estado de saude de um paciente e e facil de melhorar o estado de saude.*

**RP-PH2**:*Sim melhoria o estado de um paciente em vez de esperar muito doente e ja acabado e quando vai iniciar com tratamento hade ser dificil de melhorar e bom fazer tratamento cedo.*

**RP-PM3**:*Sim melhoria o estado de saude de paciente quando vir iniciar com tratamento antes de ficar muito doente ,melhoria a saude de um doente quando descobrir cedo e ir ao hospital cedo e logo iniciar com tratamento cedo.*

- 1. **Acha que fazer o teste de HIV e iniciar o TARV melhoraria o estado da vida do paciente? Explique?**

**RP-PH2:***Sim é bom fazer teste cedo e logo inciar com tratamento cedo.*

**RP-PM5***:Sim é bom fazer teste cedo e logo iniciar cedo com tratamento antes daqule bichinho roer seu corpo. E muito bom conservar sua saude ,porque ha vezes em que pensa que e malaria enquanto nao e malaria e outra doença.*

**RP-PM3**:*E muito bom fazer teste cedo e logo iniciar com tratamento cedo vai melhorar cedo sua saude tambem nao vai sofrer muito.*

**RP-PM1**:*E muito bom iniciar com tratamento cedo nao vai sofrer muito com doenca.*

1. **Tem mais alguma coisa a acrescentar sobre o que já discutimos?**

**RP-PH2:***Gostaria de acrescentar alguma coisa como cada doente deve receber farinha de papa soja porque esse medicamento provoca muita fome.*

**RP-PM1***:Gostaria que a U.S distribui-se farinha de papa soja para os doentes.*

**RP-PH2**:*Porque esse medicmanto de HIV,e TB nao tem outro tipo de medicamento,para pelomenos atenoar a dosagem porque esse medicamento e muito maior a carga e grossa do propio medicamento esta exagerado e muito grande.*

**MUITO OBRIGADO (A) Hora do fim da entrevista:***10:52*
