# Supplementary material for: Barriers to access and adherence to tuberculosis services, as perceived by patients: A qualitative study in Mozambique
Source: PLoS One. 2019 Jul 10;14(7):e0219470. doi: 10.1371/journal.pone.0219470 (PMC6619801; doi:10.1371/journal.pone.0219470)
Supplement: S1 Dataset — (ZIP) [file pone.0219470.s003.zip › Transcripts TB study/DGF8_.docx]

**"Avaliação da Cascata de Cuidados de Pacientes Diagnosticados com TB, MDR-TB e Paciente Co-infectados com TB/HIV nas Províncias de Manica e Sofalaʺ**

# Instrumento: Guião De Entrevista para Grupos Focais - DGFs

**Data*:****09/03/16*

**Distrito:**Chimoio

**Nome da Unidade Sanitária**:*C.S.E.M*

**Hora do início:***08:46*

**Hora do fim:***10:36*

**Número de DGF:***08*

**Legenda**

**E:** Pergunta do(a) Entrevistador(a)

**P:** Participante/entrevistado(a)

**RP:** Resposta do(a) participante/entrevistado(a)

**PH:** Participante Homem (seguido de sua posição de assento)

**PM:** Participante Mulher (seguida de sua posição de assento)

**n/a :** Não Aplicável

| Comentários/Observações Preliminares: *(circunstâncias que poderão influenciar a entrevista, etc.)Comentario a entrevista com grupo fical foi deita nos escritorio da HAI em baixo de uma abacateira com 5 participante.* |
| --- |

**14: GUIÃO DE ENTREVISTA PARA GRUPOS FOCAIS COM PACIENTES TB, TB-MR e TB-HIV**

**SECÇÃO A: ASSISTÊNCIA DO SERVIÇO DE SAÚDE AOS PACIENTES COM TB, MR-TB E TB-HIV**

1. **O que você sabe sobre TB?**

**RP-PM1:***T.B e uma doenca contajiosa atravez de respiracao quando estiver perto com um doente de TB ele quando estiver a tossir deve tapar a boca ,deve ter muito higene,mesmo quando ker falar deve tapar a boca.*

**RP-PM2**:*T.B A outra coisa quando estivermos dentro de uma sociedade devemos usar recipiente individuais, como prato copo, não devemos partilhar recipiente domestico com pessoas que tem TB.Mesmo ao tossir aquele escaro grosso deve tapar porque quando alguém pizar pode contrair outra pessoa TB.*

**RP-PH3**:*T.B é um virus que se transmite atravez do ar,quando tossir deve tapar a boca deve ter higene,porque é facil contrair TB a outra pessoa.*

**RP-PH4**:*T.B há veze em que vem com HIV de fora de casa, devem separar cobertura com as criancas, ao tossir deve tapar a boca, mesmo ao beber agua, não podemos restar no copo e dar outra pessoa deve deitar.Deve escarar no sitio e lavar esse escarador e pôr a secar no sol.*

**RP-PH5**:*T.B é uma doençaa perigosa ,podemos apanhar nas bebidas aquela coisa de trocar copos,com bebida,troca de cigarro com 5 pessoas que tem a TB .Devemos ter nossos objectos individuais ,ter cuidado com pessoa para não contrair a mesma doença,mesmo ao tossir devemos tapar a boca.*

1. **O que você sabe sobre TB- MR?**

**RP-PH3:***T.B-MR quando uma pessoa que tem TB-MR deve evitar fazer relacões sexuais ,avitar fumar e deve cuidar o sim propio mesmo.*

**RP-PH4**:*Não fazer sexo,primeiro ponto você sabe que es doente de TB-MR,primeiro deve comprir com tratamento,não deve desistir o tratamento ,não deve beber ,não deve fumar quando tem esse TB-MR, não deve comer piripiri.*

**RP-PH3**:*Não deve perder sono,quando tem essa doença de TB-MR.*

**RP-PM2:** *TB-MR se você seguir com a dosagem segundo a indicação do médico la no hospital voce melhora. Porque o médico aconselha deve tomar medicamento antes de comer nada e não deve falhar na toma de medicamento*.

**RP-PM1**:*Quando tem TB-MR,deve comprir com tratamento, não quando ver que ja sente melhor deixa de tomar medicamento.É melhor comprir porque aquela doença esconde e voce pensa que ja está melhor enquanto não esta melhor e deve comprir com todo tratamento.*

1. **O que acha sobre os serviços prestados neste sector de TB?**

**RP-PH5:** *Os serviços nesse sector de TB, eu acho que esta andar mal ,porque nos somos falado que devemos chegar cedo no hospital ,mas o enfermeiro chega muito tarde 9horas.Enquanto nos chegamos cedo e ficamos a espera dele aqui no hospital isso acontece muitas vezes.*

**RP-PH4:***O serviço prestado é isso que acabou de falar o colega aqui que essa coisa de atrazo no atendimento,mas tratamento esta tudo bem.Eu por exemplo quando cheguei aqui estava muito mal doente,mas agora ja estou a melhorar com esse tratamento.E devemos vir aqui no hospital antes de comer nada,e quando chegamos aqui ficamos muito tempo a espera para chegar e chega hora que ele quer muito tarde.Só lamentar que o enfermeiro deve melhorar essa parte de atrazo no atendimento.*

**RP-PH3**:*Os serviços neste sector é verdade que o enfermeiro diz para nos chegarmos muito cedo enquanto ele chega muito tarde,e nós doente ficamos muito tempo a espera dele .e inicia com tratamento muito tarde.Mas esse medicamento esta servir muito bem para os doentes so essa coisa de atrazo no atendimento .E o medicamento é muito forte. E gostaria recebe-se farinha de papa soja para cada doente,enquanto porque enfermeiro recomenda devemos vir aqui sem comer nada e depois ele atraza atender os pacientes.*

**RP-PM2**:*Os serviços estão andar mal,o problema maior é atrazo no atendimento,ja ouvi falar que na Beira atende muito cedo os doentes de TB e também recebe farinha de papa soja .Porque há doente que vem aqui sem nada ne tem nada de comer em casa agora com esse tratamento é preciso comer.*

**RP-PH5**:*Os serviço está andar muito mal,problema é de atrazo no atendimento.E gostaria que pelo menos recebecemos farinha de papa soja,porque existem doente que vivem sozinhos e desarascam sozinho a comida agora está doente como vai aranjar comida é dificil e em casa não tem nada para comer.*

**RP-PH2**:*Os serviços estão mal com essa coisa de atrazo no atendimento.Gostaria que muda-se esse sistema de vir tomar aqui medicamento devemos ir tomar em casa. Pelo menos nos dar medicamento de 4 dias para tomar em casa.Mas tamebém há outros doentes que são dado medicamento quando ja chega em casa ja não tomam medicamento. Outra coisa o enfermeiro chega tarde e nos ficamos até 11 horas.*

**RP-PM2**:*Há outros doentes que toma em casa medicamento.E essa coisa de esperar aqui até 11hora não esta dar para um doente.*

**RP-PH3**: *Há vezes em que não temos medicamentos e dizem deve esperar por medicamento isso também cria transtorno para um doente.*

**RP-PM1:***Gostaria que melhora-se essa parte de horario no atendimento.*

**RP-PH4**:*Aquela porta deve chegar cedo pelomenos 7:30 para atender cedo os paciente com tempo e hora aos paciente de TB.*

1. **Algum dia teve qualquer dificuldade durante o processo para acesso aos serviços de TB, TB-MR? Explique.**

**RP-PM2*:****Durante o processo os serviços de TB,TB-MR sim teve dificuldade comecei a ficar doente em Abril de 2015. Adoeci muito fazia analise não acusava nada TB, só em Outubro é quando saiu resultado positivo de TB e logo iniciou com tratamento.*

**RP-PH3**:*Dificuldade tive sim porque primeiro nos primeiros dia não acusava nada e fiz muitas analises de TB,fiz 2 analises. A terceira vez foi quando acusou TB positivo e a dotora levou-me para aquela porta de PNCT,para iniciar com tratamento de TB até hoje estou ja me sentir melhor. A nossa dificuldade é essa coisa de atrazo no atendimento o enfermeiro obriga chegarmos cedo aqui antes de comer nada, e depois ele chega aqui muito tarde.Isso também cria constragimento para um doente,mas o tratamento esta tudo bem,so essa parte de atrazo no atendimento.*

**RP-PH4**: *Durante o processo aos serviços,quando cheguei aqui no hospital primeiro fui ao laboratorio depois de laboratorio fui na PNCT,para iniciar com a toma de medicamento e ja me sinto melhor com esse tratamento.Só todos doentes reclamam essa parte de atrazo no atendimento.*

**RP-PM1**:*Durante o processo não tive dificuldade iniciou com seu tratamento e ja estou melhor só essa parte de atrazo no atendimento.*

**RP-PH5**: *Durante o processo os serviços quando cheguei aqui tinha sintomas de dore de cabeça e foi dado medicamento e fiz analise não acusava nada. Trocou de hospital e fui no hospital provincial fiquei internada no hospital duas semana fui dado alta para casa.Um visinho viu meu* *estado de saúde e disse vai no educardo mondlane fazer analise,quando cheguei aqui fiz analise e acusou resultado positivo TB,e logo inicou com tratamento de TB ate hoje .*

1. **O que sabe sobre HIV?**

**RP-PM2**:*HIV é uma doença contagiosa atravéz de relação sexual,objecto cortantes.*

**RP-PH3**:*HIV é uma doença que se transmite por varias coisas laminas,agulha,sexo não protegido.*

**RP-PH4***:HIV é transmitido atravez de relação sexual sem proteção,lamina que cortou uma pessoa que tem HIV,agulha que picou pessoa com HIV.*

**RP-PM1**:*HIV é transmitido atravéz de sangue, contaminado de HIV.*

**RP-PH3:***HIV, é o viru, não atua naquele instante pode ficar muito tempo no corpo só depois de um tempo é quando sobre sai o HIV.*

**RP-PM2**:*Por exemplo HIV,uma pessoa pode apanhar atravez de sangue contaminado.*

**RP-PH4**: *A pessoa que doa sangue primeiro deve fazer teste de HIV, depois de resultado é quando pode doar sangue para outra pessoa doente. Porque essa coisa de doar sangue pode doar sangue contaminado e pode contrair aquele doente que nao tem HIV*.

1. **O que foi mais dificil em compreender sobre TB e TB-MR?**

**RP-PH5**: *Sobre TB e TB-MR é aquilo que acabamos de falar que deve evitar fazer relação sexuais e quando tem aquelas cerimonias quando há falecimento e não cumpre com as cerimonias .*

**RP-PH3***:É quando cumpre com as cerimonias mesmo comprindo sempre aparece TB.*

1. **Como é que pode ser feito o aconselhamento para ajudar um paciente a seguir com o tratamento de TB?**

**RP-PH5**: *Para um paciente seguir com tratamento de TB primeiro deve ser aconselhado para não desanimar e não desistir o tratamento deve comprir com aconselhamento.*

**RP-PH3**:*Deve seguir bem o aconselhamento e seu tratamento.*

**RP-PH4**:*Deve seguir a hora de toma de tratamento.*

**RP-PM2**: *TB é uma doençaa simples e se você comprir passa. bastar comprir com tratamento durante 1 mês,mas esse tratamento é muito forte. Mas depois de 6 meses de tratamento passa.Mas só sofre no primeiro mês de tratamento.*

**RP-PH4**: *É dizer que olha TB, um doente deve vir no hospital todos os dias a tomar medicamento e sair em casa sem comer nada,vir tomar aqui no hospital o tratamento.*

**RP-PM2**:*Deve seguir bem o aconselhamento do medico e a dosagem também de medicamento*.

**RP-PH3:***Moralizar o doente para que não abandona o tratamento.*

**RP-PM2***:Nao descriminar o doente,nao usar violencia verbal ao doente.*

**RP-PH5**:*Deve comprir com as regras do hospital aquilo que e aconselhamento de medico.*

**RP-PH4**: *Aqui ser aconselhado que olha chega 6hora sem comer nada e o enfermeiro ele chega tarde depois dele comer.*

**RP-PM1**: *Não devemos abandonar medicamento,um enfermeiro deve respeitar o paciente*.

**RP-PM2**:*Aconselhar doente que olha quando se sentir melhor não deve abandonar medicamento ,não fumar ,não beber evita relação sexual não consumir coisa muito doce faz mal.*

**SECÇÃO C: ADESÃO AOS SERVIÇOS TB**

***(Geralmente é difícil para muitos pacientes aderirem ao tratamento TB,TB-MR e TB/ HIV).***

1. **Quais são os problemas que os doentes enfrentam para iniciar o tratamento com:**

**i) TB?**

*Nao aplicavel.*

**ii) TB-MR?**

*Nao aplicavel.*

**iii) TB- HIV?**

**RP-PH4:***Quando um paciente,tem TB e vai fazer teste acusa sempre HIV.*

**RP-PM2**: *Ja ouvi falar que sintoma de TB é mesmo sintoma de HIV,sente também dores de cabeça….*

**RP-PM1**:*Essa pergunta e complicada de responder.*

1. **Quais são os aspectos que foram mais difíceis para continuar a fazer o tratamento?**

**RP-PM2:** *Aspecto que foram mais dificeis para continuar a fazer o tratamento e a reação do medicamento e forte e provoca dor nos menbros.*

**RP-PH3**:*Aspecto dificeis para continuar a fazer tratamento,o medicamento e forte provoca dor nos menbros.*

**RP-PM1**:*Dificeis a continuar a fazer tratamento por causa de reações desse medicamento é muito forte, mas isso de reações depende de organismo de cada pessoa há vezes em que fica escura,inchasso nos pés,comichão são reações depende de organismo de cada um*.

**RP-PH4**: *Dificil de continuar com tratamento essa reações do medicamento provoca dor de joelho.*

**RP-PM2**: *Dificil de continuar com tratamento porque tem mudança de temperatura,ora aquece muito corpo,ora sente frio.*

**RP-PH5**: *Dificil a continuar com tratamento há vezes que fica falta de apetite.*

**RP-PH4**: *Aspecto mais dificeis para continuar com tratamento é ,esse tratamento tem reações de sono mas essa reações e muito mas nos primeiro dias de tratamento, mas depois de 2 meses passa essa reação nem sono acaba tudo.*

**SECÇÃO D: MELHORAR O LABORATÓRIO E PNCT**

1. **Existe algo que poderia ser melhorado nos serviços de PNCT?**

**RP-PM1:** *Nos serviços de PNCT devem melhorar o horário de atendiemento.*

**RP-PH5**: *Deve melhorar pelomenos trabalhar 2 enfermeiro para melhorar o atendimento,ter activista que distribui medicamento para doente de TB. Demora atender porque chega filha dele primeiro atende filhos dele depois e quando atende a nos tambem agora isso nao da para paciente.*

**RP-PH4**:*Activista chega aqui na porta de PNCT,entra só e nos ficamos a espera de ser atendidos e enfermeiro primeiro atende esse activista e depois é quando atende a nos paciente, agora isso cria constragimento para um paciente.*

**RP-PH5**:*Sim as activista tem direito de ser atendido primeiro,depois é quando atende a nos também,e primeiro ele quando chega faz coisa dele primeiro depois e quando começaa a tender paciente,enquanto ja estamos cansado de esperar.*

**RP-PM2**:*A parte focal dele é chegar cedo e nos atender,porque nos tomamos medicamento ai no hospital,e deve ser muito flexivel no atendimento aos paciente.*

- 1. **O que deve ser feito pela US na selecção ao tratamento e sua continuidade?**

**RP-PM2:***A U.S na seleção de tratamento pelo menos dar medicamento de uma semana para ir tomar em casa.*

**RP-PH3**:*A pessoa que atende nesse sector de PNCT tem razao de nao dar medicamento para ir tomar em casa,porue ha pacientes que quando chega em casa ja nao toma o medicamento.*

**RP-PH5**:*Essa doenca traz muito cansanco nao sei se e reacao de medicamento*.

**RP-PH4**:*Ha doente que vive longr,nao tem transporte e deve vir aqui todos os dias tomar medicamento.Agora se voce recebe medicamento e voce chega em casa nao toma,nao esta prejudicar enfermeiro esta prejudicar a sua saude mesmo.*

- 1. **O que o trabalhador de saúde poderia fazer para melhorar aderência ao tratamento?**

**RP-PH3**:*O trabalhador de saude para melhorar a derencia deve moralizar ao doente ,encoranjar ao doente,que olha nao podes abandonar medicamento.no caso tenha doente gravemente deve dar prioridade a esse doente grave doente a ser primeiro a se atendido.*

**RP-PH4**:*Talvez melhorar essa parte quando um servente ver um doenteque nao consegue andar ajudar a esse doente aconpanhar ate a porte do enfemeiro,porque ha vezes em que um servente vem um doente bem mal doente mas acompanha na porta do medico*.

**RP-PM2**:*Essas serventes ha vezes em que viola doente verbalmente .*

**RP-PH4:***Ha vezes em que um doente esta muito mal doente mas manda entrar primeiro familia dele ,e esse servente tambem atende mal tambem aos doentes em vez de piorizar aquele doente que esta malpara apanhar tratamento,para ir em casa .*

**RP-PM2**:*Deve melhorar essa parte de atrazo no atendimento isso ajudaria muito na aderencia de um doente a ir fazer tratamento,ajudaraia muito na aderencia .*

1. **Acha que fazer o diagnóstico e tratamento imediato da tuberculose melhoraria o estado de saúde do paciente? *(Sondar: como? Ou de que maneira?*)**

**RP-PM2:***Acho que e melhor fazer tratamento a tempo e hora em vez de esperar ficar muito doente ,logo que sentir que esta doente e melhor fazer tratamento imediato.*

**RP-PH4**:*Acho e melhor cedo fazer diagnosticoe logo iniciar com tratamento agora se voce ir tarde custa para melhorar a doenca.e melhor enquanto e cedofazer diagnostico e iniciar com tratamento.*

**RP-PM2**:*Fazer tratamento imediato melhoria o estado de paciente mas rapido,porque se voce permanece em casa voce nao vai saber que e TB,e partilha mesmo objecto com a familia sem saber que contaminar muita gente .E aconselhavel fazer diagnostico e logo iniciar com tratamento rapido.*

**RP-PM1**:*E muito bom saber o seu estado de saude .*

**RP-PH4**:*E muito bom,porque voce fez teste e sabe que e positivo TB,e nao quer tomar medicamento estara a se prejudicar a si mesmo. E voce desconfiar que tem TB e melhor iniciar com tratamento de TB,em vez de esperar acabar corpo nao bom.*

**RP-PM2**:*Melhoria a saude do paciente mas rapido em vez de permanecer em casa ,acontaminar outras pessoas,quando se sentir doente e melhor ir cedo ao hospital para tratamento.*

**RP-PH3**:*Melhoria,sim a saude de paciente,eu descobri cedo e logo iniciei com tratamento e ja estou a melhorar e estou a comprir com tratamento nao brinco mal e estou melhorando.*

- 1. **Acha que fazer o teste de HIV e iniciar o TARV melhoraria o estado da vida do paciente? Explique?**

**RP-PM2:***Sim melhoria o estado de vida do paciente a iniciar cedo com tratamento e bom e é mais rapido.*

**RP-PM1***:E muiro bom saber o seu estado de suade e logo iniciar com tratamento de TARV,melhoraria a saude .*

**RP-PH4**:*E muito bom porque iniciar comTARV e seguir com tratamento melhoraria o estado de vida de pacienteem vez de esperar acabar corpo nao e bom.*

**RP-PH5**:*Sim o paciente deve iniciar com TARV,para melhorar o estado de saude.*

**RP-PM2**:*Quando faz teste e logo iniciar com TARV sim melhoria,o estado de vida de pacienteem de esperar perderkilos .E melhor iniciar cedo com tratamento.*

1. **Tem mais alguma coisa a acrescentar sobre o que já discutimos?**

**RP-PH4**:*Gostaria de acrescentar na parte do TB essa doenca,um doente começa de laboratorio,PNCT ,farmacia .Mas aquele enfermeiro de PNCT,tem deixado doente da prioridade primeiro coisas deles*.

**RP-PM2**:*Acto de fala sai vai converssar com outros colegas ora esta atender telef com amigos deles.*

**RP-PH3**:*Ha vezes em que chega uma pessoa que tem dienheiro e o primeiro a ser atendido.*

**RP-PH4**:*Eu nao tenho conhecido e so esperar ate ele atender .*

**RP-PM2:***Um cidadao tem direito de comprir bicha.*

**RP-PH5**:*Ai funciona muito ser conhecido,e suborno tambem.*

**MUITO OBRIGADO (A) Hora do fim da entrevista:***10:36*
