# Supplementary material for: Barriers to access and adherence to tuberculosis services, as perceived by patients: A qualitative study in Mozambique
Source: PLoS One. 2019 Jul 10;14(7):e0219470. doi: 10.1371/journal.pone.0219470 (PMC6619801; doi:10.1371/journal.pone.0219470)
Supplement: S1 Dataset — (ZIP) [file pone.0219470.s003.zip › Transcripts TB study/DGF9_.docx]

**"Avaliação da Cascata de Cuidados de Pacientes Diagnosticados com TB, MDR-TB e Paciente Co-infectados com TB/HIV nas Províncias de Manica e Sofalaʺ**

# Instrumento: Guião De Entrevista para Grupos Focais - DGFs

**Data:** 22. 03. 2016

**Distrito:** Gondola

**Nome da Unidade Sanitária**: H. D. Gondola

**Hora do início:** 09H:24

**Hora do fim:** 11:46

**Número de DGF:** 09

**Legenda**

**E:** Pergunta do(a) Entrevistador(a)

**P:** Participante/entrevistado(a)

**RP:** Resposta do(a) Participante/entrevistado(a)

**PH:** Participante Homem (seguido de sua posição de assento)

**PM:** Participante Mulher (seguida de sua posição de assento)

**n/a :** Não Aplicável

| Comentários/Observações Preliminares: *(circunstâncias que poderão influenciar a entrevista, etc.)* *A DGF decorreu num bom lugar, com privacidade, calmo e onde os participantes deram o seu contributo sem receio.* |
| --- |

**SECÇÃO A: ASSISTÊNCIA DO SERVIÇO DE SAÚDE AOS PACIENTES COM TB, MR-TB E TB-HIV**

1. **O que você sabe sobre TB?**

**RP-PM1:** *TB é uma doença contagiosa provocada atravéz de respiração, quando estiver perto com um doente de TB ele quando estiver a tossir deve tapar a boca ,deve ter muita higiene ,mesmo quando quiser falar deve tapar a boca.*

***RP-PH6:*** *TB é uma doença que se identifica por tosse há mais de uma semana, febres, respiração funda nas subidas, falta de apetite, e por vezes quando estás a subir, a pessoa tem tido respiração funda.*

***RP-PM3:*** *TB é uma doença que provoca cansaço, o doente não respira bem, tem tosse e o seu corpo fica fraco.*

***RP-PM2:*** *TB é uma doença que cansa o corpo, provoca tosse e respiração rápida.*

***RP-PH4:*** *Essa doença é provocada por uma pessoa contaminada, quando tosse ao lado de uma pessoa sã. Quando anda-se descalço, você pode pisar escarro de um doente com TB e ficar contaminado. Também fumo de cigarro e drogas pode levar a TB.*

***RP-PH1:*** *TB é uma doença que nós não sabemos de onde vem. Muitos dizem que é uma doença provocada por piringanisso. Quando você faz cupita-kufa, vai ao curandeiro, faz tratamento não cura. Só quando vamos ao hospital é que nos dizem que é TB. Tenho um filho de 15 anos, e teve essa doença, mas ele não conhece mulher e nunca fez relações sexuais. Só o hospital é que trata a doença.*

**2 O que você sabe sobre TB- MR?**

**RP-PH3:** *TB-MR quando uma pessoa que tem TB-MR deve evitar fazer relações sexuais, avitar fumar e deve cuidar-se a si mesmo.*

***RP-PH6:*** *Esta TB é difícil de curar porque o bicho está escondido nos ossos. Há vezes que alguns pacientes quando fazem o tratamento não cumprem, acabam abandonando, e quando estes manifestam a recaída dificilmente curam e a doença vem muito forte.*

***RP-PM2:*** *Eu nunca ouvi falar dessa TB-MR.*

***RP-PH1:*** *Eu tambem nunca ouvi falar dessa doença.*

***RP-PM2:*** *Quando a pessoa tem TB-MR sente muita dor nas ariticulaçoes.*

***RP-PM3:*** *Eu ainda não ouvi falar dessa TB.*

**3 O que acha sobre os serviços prestados neste sector de TB?**

**RP-PH5:** *Os serviços nesse sector de TB, eu acho que esta andar mal, porque nós somos falado que devemos chegar cedo no hospital, mas o enfermeiro chega muito tarde 9horas. Enquanto nós chegamos cedo e ficamos a espera dele aqui no hospital isso acontece muitas vezes.*

**RP-PH4:** *O serviço prestado aqui neste sector é isso que acabou de falar o colega aqui que essa coisa de atraso no atendimento ,mas tratamento esta tudo bem. Eu por exemplo quando cheguei aqui estava muito mal ,mas agora ja estou a melhorar com esse tratamento. E devemos vir aqui no hospital antes de comer nada, e quando chegamos aqui ficamos muito tempo a espera para chegar e chega hora que ele quer muito tarde. Só lamentar que o enfermeiro deve melhorar essa parte de atraso no atendimento.*

***RP-PM3:*** *Neste sector estão a trabalhar bem, dão-nos os comprimidos muito bem, dão palestra, aconselham, apenas nós é que devemos seguir estes conselhos para o nosso bem.*

***RP-PM5:*** *Este sector trabalha muito bem, não temos problemas, dão aconselhamento, trazem os comprimidos em casa em todas as segundas-feiras. Se você não toma é da sua conta, mas o hospital está atendendo muito bem.*

***RP-PM2:*** *Este setor está a trabalhar muito bem, não tenho razões de queixas. Quando você não consegue andar, em todas as segundas-feiras trazem comprimidos para tomar. Tem ativistas que nos trazem os comprimidos em casa.*

***RP-PH1:*** *Este setor nos trata bem, até os ativistas trazem-nos medicamentos em casa, e aqui no hospital tomamos comprimidos no setor, eles dão-nos água e tomamos. Quando viemos ao hospital, é mais para fazer controlo mensal. Quanto aos medicamentos as ativistas trazem em nossas casas.*

***RP-PH4:*** *As ativistas vêm dar-nos os comprimidos em casa todos os dias. Estão a tratar-nos bem.*

*RP-PH6: Os enfermeiros têm paciência connosco. Dão aconselhamento para que saibamos cuidarmo-nos da doença. Estão a tratar-nos bem.*

1. **Algum dia teve qualquer dificuldade durante o processo para acesso aos serviços de TB, TB-MR? Explique.**

**RP-PM2*:*** *Sim teve dificuldade comecei a ficar doente em Abril de 2015. Adoeci muito fazia analise não acusava nada TB, só em Outubro é quando saiu resultado positivo de TB e logo iniciei o tratamento.*

**RP-PH3**: *Dificuldade tive sim porque primeiro dia não acusava nada e fiz muitas analises de TB ,fiz 2 analises. A terceira vez é quando acusou TB positivo e a dotora levou-me para aquela porta de PNCT, para iniciar com tratamento de TB, até hoje estou a fazer o tratamento e ja me sinto melhor. A nossa dificuldade é essa coisa de atraso no atendimento o enfermeiro obriganos para chegarmos cedo aqui antes de comer nada, e depois ele chega aqui muito tarde.Isso tambem cria problema para um doente. Mas o tratamento esta tudo bem, só essa parte de atraso no atendimento.*

**RP-PH4**: *Durante o processo aos serviços, quando cheguei aqui no hospital primeiro fui ao laboratório depois de laboratório fui na PNCT, para iniciar com a toma de medicamento e ja me sinto melhor com esse tratamento. Só todo doente reclama essa parte de atraso no atendimento.*

***RP-PH1:*** *Dificuldades sempre existem. Apenas andei cinco dias para receber o resultado da análise e iniciar o tratamento.*

***RP-PH6:*** *Tive dificuldades porque fiz análises por duas vezes e sempre tive resultado negativo. A terceira vez enviaram-me ao Hospital Provincial de Chimoio, fiz a análise e deu positivo e iniciei o tratamento.*

***RP-PM5:*** *Eu tive dificuldades porque vim fazer consulta, deram-me comprimidos brancos, tomei e não melhorei. Vim pela segunda vez, deram-me frasco para pôr escarro, fizeram a análise e o resultado foi positivo, em seguida iniciei com o tratamento.*

***RP-PH4:*** *Tive dificuldades porque quando comecei fizeram análise e não acusou nada. Mandaram-me fazer teste de HIV, também tive resultado negativo, foi quando me referiram ao Hospital Provincial de Chimoio para fazer análise de TB, e deu positivo, em seguida iniciei o tratamento.*

***RP-PM3:*** *Eu tive muitas dificuldades, porque andei cinco vezes e sempre deram-me comprimidos brancos e por vezes de tensão, e nunca ficava melhor. Só a sexta vez é que deram-me frascos para escarrar neles. Fiz a análise de tosse e acusou TB, e comecei imediatamente a fazer o tratamento.*

***RP-PM2:*** *Tive dificuldades.*

1. **O que sabe sobre HIV?**

***RP-PM3:*** *HIV é uma doença provocada por relações sexuais ou por objetos perfuro-cortantes.*

***RP-PH6:*** *HIV é uma doença provocada por objetos cortantes como agulhas e tesouras.*

***RP-PH1:*** *HIV é uma doença provocada por lâminas, agulhas e outros objetos. Tambem é provocada por pessoas, por exemplo quando você namora muito e tem relaçoes sexuais. Se você se previne não vai apanhar a doença.*

***RP-PH4:*** *HIV pode apanhar-se quando você vai ao hospital e apanha injeções não fervidas que foram usadas numa pessoa infetada.*

***RP-PM3:*** *HIV pode provocar-se por injeções, agulhas, relações sexuais não protegidas por preservativo.*

***RP-PH4:*** *Por vezes nós vamos ao curandeiro e usamos lâminas que cortaram uma pessoa infetada pelo vírus HIV, nessas condições acabamos por também contrair o HIV.*

1. **O que foi mais dificil em compreender sobre TB e TB-MR?**

**RP-PH5**: *Sobre TB e TB-MR é aquilo que acabamos de falar que devemos evitar fazer relação sexual e quando tem aquelas cerimonias de falecimento e não compre com as cerimonias .*

**RP-PH3***: É quando compre com as cerimonias mesmo comprindo sempre aparece TB.*

***RP-PM5:*** *Foi muito dificil compreender essa doença devido aos sintomas que são varios numa so vez.*

***RP-PH1:*** *É muito difícil compreender porque muita das vezes a pessoa não sabe quando é que é TB e quando é que é HIV. Essa TB traz muitos sintomas, dores do peito, pernas e todo o corpo fica cansado.*

***RP-PM3:*** *É o que falou este papa. Muitas vezes nós não sabemos o que sentimos, são várias doenças no individuo, só quando chegamos ao hospital é quando nos dizem que é TB.*

***RP-PM2:*** *É muito difícil compreender essa doença, nem como ela se apanha. Por vezes é acompanhada por vários sintomas. É difícil saber que é TB.*

***RP-PH4:*** *É difícil compreender porque essa doença começa por uma constipação, depois é quando vens ao hospital. Depois de a tosse resistir muito, o hospital te manda fazer análise de TB, se o resultado for positivo começa a fazer o tratamento.*

1. **Como é que pode ser feito o aconselhamento para ajudar um paciente a seguir com o tratamento de TB?**

**RP-PH5**: *Para um paciente seguir com tratamento de TB primeiro deve ser aconselhado para não desanimar e não desistir do tratamento e deve comprir com aconselhamento.*

**RP-PH3**: *Deve seguir bem o aconselhamento e seu tratamento.*

**RP-PH4**: *Deve seguir a hora de toma de tratamento.*

***RP-PM2:*** *O aconselhamento deve ser feito com carinho. Os enfermeiros devem incentivar os pacientes, dizendo que as reções são passageiras e que os pacientes devem cumprir com o tratamento . Pelo que vejo muitos abandonam por falta de comida, uma vez que os comprimidos provocam muita fome. Os enfermeiros devem encorajar os pacientes a terem paciência ao administrarem o tratamento aos pacientes.*

***RP-PH4:*** *Fortificar o aconselhamento*

***RP-PH1:*** *O enfermeiro dá-nos muito aconselhamento, muitos desistem quando sentem-se melhor, antes mesmo de o tratamento terminar.*

***RP-PH6:*** *Os ativistas devem assistir os pacientes a tomarem os medicamentos em casa. Fazer uma surpresa, isto é, as ativistas fazerem uma surpresa ao paciente para controlar quantas lâminas de comprimidos foram consumidas.*

***RP-PM5:*** *As pessoas não devem ser ditas para tomarem os comprimidos, cada um é responsável por si mesmo.*

***RP-PH1:*** *Se os enfermeiros pedem-nos para vir ao hospital, é mais para fazer o controlo, isto é, pesar, aconselhar e mais. Muitos quando se sentem bem abandonam o tratamento, até vão beber Nipa. Deve aconselhar-se a comer bem, não fazer serviço forçado e aconselhar a vir ao hospital.*

***RP-PM2:*** *É importante aconselhar a vir sempre ao hospital, dar palestras, encorajar os pacientes dizendo que essa doença tem cura. Fazer visitas em casa e envolver a família no tratamento, isto é, a família fazer acompanhamento da toma dos comprimidos por parte do seu familiar doente.*

**SECÇÃO C: ADESÃO AOS SERVIÇOS TB**

***(Geralmente é difícil para muitos pacientes aderirem ao tratamento TB,TB-MR e TB/ HIV).***

1. **Quais são os problemas que os doentes enfrentam para iniciar o tratamento com:**
2. **TB?**

**RP-PH4: *É*** *quando um doente que tem TB e vai fazer teste acusa sempre HIV.*

***RP-PH6:*** *Muitos não iniciam o tratamento por má concepção, porque confiam no curandeiro, não confiam no tratamento, outros invocam espíritos e outros é por negligência.*

***RP-PH1:*** *Algumas pessoas não iniciam o tratamento porque acham que o hospital vai interditar muitas coisas sobretudo fumar. Outros é pelo hábito de frequentarem no curandeiro, sempre são aldrabados pelos curandeiros. Por vezes são ditos que estão doentes por conta de sua família que morreu, outros porque não fizeste boa cerimónia.*

***RP-PM2:*** *Muitos porque não têm confiança com o tratamento hospitalar. Muitos deles chegam tarde porque primeiro vão ao curandeiro, só depois é que decidem vir ao hospital.*

***RP-PM3:*** *Muitos não iniciam o tratamento por causa de espíritos, e por causa de preguiça de vir ao hospital todos os dias.*

1. **TB-MR?**

*n/a*

1. **TB- HIV?**

*n/a*

1. **Quais são os aspetos que foram mais difíceis para continuar a fazer o tratamento?**

**RP-PM2:** *Aspecto que foram mais dificeis para continuar a fazer o tratamento são reações do medicamento que são forte e provoca dor nos membros.*

**RP-PH3**: *Aspecto mais dificeis para continuar a fazer tratamento, são medicamento que são fortes provocam dor nos joelhos.*

**RP-PM1**: *Dificil de continuar a fazer tratamento por causa de reações desse medicamento que são muito forte, mas essas reações, depende de organismo de cada pessoa, há vezes em que fica escura, inchasso nos pés,comichão são reações e estas dependem de organismo de cada um*.

***RP-PH6:*** *Muitos não continuam por causa das distâncias dos doentes. Eles não conseguem chegar ao hospital, outros é pela reação dos medicamentos, porque estes são muito fortes. Os remédios provocam dores, principalmente dores das articulações, essas dores não são para desafiar com elas. Por exemplo aquele senhor que voltou aqui recebe os comprimidos, mas por vezes não toma porque sua sequência alimentar é muito fraca.*

***RP-PH1:*** *Muitos começam a ver a interdição de seus vícios e/ou hábitos. Muitos começam a ver o enfermeiro a proibir a beber, a manter relações sexuais, a comer peixe seco, feijão com muito sal. Isto dificulta a continuidade do tratamento.*

***RP-PM5:*** *É por causa da fome que outros pacientes não continuam com o tratamento, muitos não têm nada para comer em casa.*

**SECÇÃO D: MELHORAR O LABORATÓRIO E PNCT**

1. **Existe algo que poderia ser melhorado nos serviços de PNCT?**

**RP-PM1:** *Nos serviços de PNCT deve melhorar o horário de atendiemento.*

**RP-PH5**: *Deve melhorar pelo menos trabalhar 2 enfermeiro para melhorar o atendimento, ter activista para distribuir medicamentos para doente de TB. O enfermeiro demora muit atender porque chega filha dele primeiro atende filhos dele e depois é quando atende a nós também, agora isso não da para doente.*

**RP-PH4**: As *activista chegam aqui na porta de PNCT, entram só e nós ficamos a espera de ser atendidos e enfermeiro primeiro atende esses activistas e depois é quando atende a nós doente, agora isso cria problema para um doente.*

***RP-PH1:*** *Neste momento não há problema, tudo está bem. Só gostaríamos que dessem comida aos doentes porque este tratamento é muito forte. Muitas vezes os doentes com TB não vão a machamba e não fazem serviço forte.*

***RP-PM3:*** *Gostaríamos que o setor de TB distribuísse comida para os doentes porque nos não fazemos nada e a doença nos obriga a comer muito.*

***RP-PH4:*** *Os enfermeiros estão a atender muito bem, sem nenhum problema.*

***RP-PM5:*** *Os enfermeiros estão a atender bem e sem problemas, mas se você for confuso, o enfermeiro também acaba sendo confuso.*

***RP-PH6:*** *Estamos a pedir apoio alimentar, palestras na comunidade, enviar ativistas para conversar com os pacientes, e fazer controlo da doença mensalmente.*

- 1. **O que deve ser feito pela US na seleção ao tratamento e sua continuidade?**

**RP-PM2:** *O hospital na selecção de tratamento pelo menos dar medicamentos de uma semana para ir tomar em casa.*

**RP-PH3**: *A pessoa que atende nesse sector de PNCT tem razão de não dar medicamentos para ir tomar em casa, porque há doentes que quando chega em casa ja não toma o medicamento.*

***RP-PH1:*** *A seleção do tratamento é boa, porque estamos a tomar os medicamentos como deve ser, e eles devem continuar assim.*

***RP-PH4:*** *A seleção é boa, porque até estão a trazer os comprimidos em nossa casa.*

***RP-PM2:*** *O tratamento está bem, devem continuar a dar o tratamento em casa e fazer o seguimento dos pacientes.*

***RP-PH1:*** *A minha filha que fez o tratamento em 2012, quando tinha 15 anos, já está bem melhor.*

***RP-PM5:*** *A seleção dos comprimidos vão consoante o seu peso. Isto esta bem, porque nos estamos bem.*

- 1. **O que o trabalhador de saúde poderia fazer para melhorar aderência ao tratamento?**

**RP-PH3**: Para melhorar a aderência *o trabalhador de saúde deve moralizar ao doente, encoranjar ao doente que olha não podes abandonar medicão. no caso que tenha doentes graves deve dar prioridade a esse doentes graves. São esses doentes que devem ser atendido em primeoro lugar.*

***RP-PH1:*** *O enfermeiro deve saber receber as pessoas da melhor maneira, com respeito e paciência. O enfermeiro deve evitar estar sempre ao celular enquanto tem pacientes para atender. Se o paciente for bem atendido não vai desistir.*

***RP-PH6:*** *Os enfermeiros devem ser passivos, rápidos e respeitosos. O enfermeiro deve ter boa imagem para motivar os pacientes a regressarem.*

***RP-PM2:*** *Os enfermeiros devem atender cedo os doentes, para regressarmos cedo as nossas casas.*

***RP-PM2:*** *O enfermeiro deve ser paciente, tratar de uma boa maneira, e não com rugas. O paciente deve ser atendido rápido.*

1. **Acha que fazer o diagnóstico e tratamento imediato da tuberculose melhoraria o estado de saúde do paciente? *(Sondar: como? Ou de que maneira?*)**

**RP-PM2:** *Acho que é melhor fazer tratamento a tempo e hora em vez de esperar ficar muito doente, logo que sentir que está doente é melhor fazer tratamento imediato.*

**RP-PH4**:*Acho é melhor cedo fazer diagnóstico e logo iniciar com tratamento agora se você ir tarde custa para melhorar a doença.e melhor enquanto e cedof azer diagnostico e iniciar com tratamento.*

***RP-PH6:*** *Sim melhoraria, porque não agrava o estado de saúde do paciente. O paciente recupera rápido, e em pouco tempo o paciente pode curar.*

***RP-PH1:*** *Sim melhoraria. Fazer consulta, análise e ter logo o resultado e iniciar imediatamente o tratamento é muito importante, ao invés de ficar15 dias ou um mês só a andar, isto não é bom, desgasta o corpo*

***RP-PM3:*** *Sim melhoraria, porque outros chegam aqui sem conseguir andar, isto é muito difícil e feio para o hospital, assim como para o doente, e tem sido muito difícil recuperar o seu corpo.*

***RP-PH4:*** *Sim melhoraria, porque iniciar o tratamento rápido não desgasta o corpo do paciente.*

**Acha que fazer o teste de HIV e iniciar o TARV melhoraria o estado da vida do paciente? Explique?**

**RP-PM2:***Sim melhoria o estado de vida do paciente se iniciar cedo com tratamento é bom.*

**RP-PM1***: E muito bom saber sobre seu estado de saúde e logo iniciar com tratamento de TARV, melhoraria a saúde .*

***RP-PM3:*** *Sim melhoraria porque reduz a manifestação dos sintomas da doença.*

***RP-PH4:*** *Sim melhoraria porque o vírus que está no seu corpo acaba destruindo o seu corpo, então, quanto mais cedo for melhor, não gasta o seu corpo.*

***RP-PH1:*** *Sim melhoraria porque começa logo a fazer o tratamento, e o organismo melhora logo, não emagrece, não manifesta muitas doenças, e você recupera rápido.*

***RP-PM2:*** *Sim melhoraria porque o seu corpo não vai gastar-se.*

***RP-PH6:*** *Sim melhoraria, porque fazendo o teste e iniciar o tratamento, os bichinhos reduzem a sua reação.*

1. **Tem mais alguma coisa a acrescentar sobre o que já discutimos?**

**RP-PH4**: *Gostaria de acrescentar na parte do TB essa doença, o doente começa de fazer analise no laboratorio, depois passa para o PNCT e depois vai a farmacia .Mas aquele enfermeiro de PNCT, tem deixado doentes da prioridade primeiro coisas deles*.

**RP-PM2**: *Acto de fala, sai vai converssar com outros colegas, ora esta atender telefone com amigos deles nós não gostamos disso.*

**RP-PH3**: *Há vezes em que quando chega uma pessoa que tem dienheiro é o primeiro a ser atendido*

***RP-PH1:*** *Pedimos aos enfermeiros que continuem a trabalhar bem.*

***RP-PH6:*** *Pedidos que eu tenho, é que no setor de TB, deve procurar um mecanismo para o diagnóstico de TB. Eu vivo com uma criança e estou com receio de contamina-la. Já a trouxe para fazer análise, mas ela não consegue tirar escarro.*

***RP-PH1:*** *Gostaria que os enfermeiros nos atendessem bem.*

***RP-PM5:*** *Gostaria que as ativistas trouxessem análises em nossas casas, para nós não virmos ao hospital.*

**MUITO OBRIGADO (A) Hora do fim da entrevista__11H:46__**
